# *ADAMTSL2* mutations determine the phenotypic severity in geleophysic dysplasia

**DOI:** 10.1172/jci.insight.174417

**Published:** 2024-02-01

**Authors:** Vladimir Camarena, Monique M. Williams, Alejo A. Morales, Mohammad F. Zafeer, Okan V. Kilic, Ali Kamiar, Clemer Abad, Monica A. Rasmussen, Laurence M. Briski, LéShon Peart, Guney Bademci, Deborah S. Barbouth, Sarah Smithson, Gaofeng Wang, Lina A. Shehadeh, Katherina Walz, Mustafa Tekin

**Affiliations:** 1 Dr. John T. Macdonald Foundation Department of Human Genetics;; 2 Department of Medicine, Division of Cardiology;; 3 Interdisciplinary Stem Cell Institute;; 4 John P. Hussmann Institute for Human Genomics;; 5 Department of Medical Education; and; 6 Department of Pathology and Laboratory Medicine, University of Miami Leonard M. Miller School of Medicine Miami, Florida, USA.; 7 Department of Clinical Genetics, University Hospitals Bristol and Weston NHS Foundation Trust, Bristol, United Kingdom.; 8 Sylvester Comprehensive Cancer Center, University of Miami Leonard M. Miller School of Medicine, Miami, Florida, USA.; 9 IQUIBICEN - CONICET, Faculty of Exact and Natural Sciences, University of Buenos Aires, Argentina.; 10 Department of Otolaryngology and; 11 Department of Ophthalmology, University of Miami Leonard M. Miller School of Medicine, Miami, Florida, USA.

**Keywords:** Genetics, Extracellular matrix, Genetic diseases, Molecular genetics

## Abstract

Geleophysic dysplasia-1 (GD1) is an autosomal recessive disorder caused by ADAMTS-like 2 (*ADAMTSL2*) variants. It is characterized by distinctive facial features, limited joint mobility, short stature, brachydactyly, and life-threatening cardiorespiratory complications. The clinical spectrum spans from perinatal lethality to milder adult phenotypes. We developed and characterized cellular and mouse models, to replicate the genetic profile of a patient who is compound heterozygous for 2 ADAMTSL2 variants, namely p.R61H and p.A165T. The impairment of ADAMTSL2 secretion was observed in both variants, but p.A165T exhibited a more severe impact. Mice carrying different allelic combinations revealed a spectrum of phenotypic severity, from lethality in knockout homozygotes to mild growth impairment observed in adult p.R61H homozygotes. Homozygous and hemizygous p.A165T mice survived but displayed severe respiratory and cardiac dysfunction. The respiratory dysfunction mainly affected the expiration phase, and some of these animals had microscopic post-obstructive pneumonia. Echocardiograms and MRI studies revealed a significant systolic dysfunction, accompanied by a reduction of the aortic root size. Histology verified the presence of hypertrophic cardiomyopathy with myocyte hypertrophy, chondroid metaplasia, and mild interstitial fibrosis. This study revealed a substantial correlation between the degree of impaired ADAMTSL2 secretion and the severity of the observed phenotype in GD1.

## Abstract 2

ADAMTSL2 variant-espressing models&nbsp;replicate the phenotypic heterogeneity of geleophysic dysplasia in mice, with severity linked to impaired ADAMTSL2 secretion.

## Introduction

Geleophysic dysplasia (GD, OMIM 231050) is a rare genetic disorder that principally affects bones, joints, heart, lungs, and skin. Affected patients are characterized by short stature, short hands and feet, joint contractures, and a distinct facial appearance, as well as cardiac and respiratory dysfunction that can increase early mortality (1–4). Geleophysic dysplasia type 1 (GD1) is an autosomal recessive condition that is caused by biallelic variants in the *ADAMTSL2* (encoding ADAMTS-like 2) gene, on chromosome 9 at q34 (5). The same phenotype can be caused by monoallelic variants in *FBN1* (encoding fibrillin 1) or *LTBP3* (encoding latent transforming growth factor-beta-binding protein 3), which are referred to as geleophysic dysplasia type 2 (GD2) and type 3 (GD3), respectively (6, 7).

ADAMTSL2 is a member of a large family of secreted proteins with a function in the extracellular space (8–10). ADAMTS-like proteins do not have enzymatic activity because of the lack of a zinc metalloendopeptidases domain (9). ADAMTSL2 is secreted and variants in the protein may affect its secretion to the extracellular space (4, 10, 11). This protein can associate with other proteins that are part of the extracellular matrix and modulate their functions. For instance, ADAMTSL2 has been reported to bind to FBN1 and LTBP1 and might affect the microfibril assembly or the availability of TGF-β1 (5, 6, 12–17). An association of mutated *ADAMTSL2* with an increased TGF-β signaling has been reported in humans and mouse models, suggesting that the mechanism underpinning the disease is through the dysregulation of the TGF-β pathway (5, 12, 18, 19). However, other reports indicated that TGF-β increase exists in some but not all GD cases (20, 21).

An *Adamtsl2*-knockout (*Adamtsl2*-KO) mouse was the first attempt to generate a GD animal model (12, 19, 22). However, these animals do not survive after birth, limiting their use for further characterization. The KO model likely represents the most severe end of the phenotypic spectrum caused by *ADAMTSL2* mutations (21). Therefore, there is a need to generate animal models that reproduce the phenotype as characterized in most patients. Such an animal model would allow us to study the individual contribution of mutated alleles. Furthermore, future pharmacological and genetic interventions could be investigated in this animal model.

Herein, we present an individualized approach to systematically model the genotype and phenotype based on a patient clinically diagnosed with GD1. We aimed to better understand the pathology of the condition and the contribution of each allele to the phenotype. Specifically, we explored the role of each mutated *ADAMTSL2* allele in its secretion. The contribution of each allele to the phenotype was further determined with functional analyses and in vivo phenotyping of several mouse lines. We found a reduction of survival and growth, abnormalities in craniofacial and skeletal anatomy, respiratory insufficiency, and cardiac abnormalities that correlate with the impairment of mutated Adamtsl2 secretion.

## Results

### A patient diagnosed with GD1.

Retrospective data indicate that the phenotypic spectrum of GD1 is variable, ranging from very severe cases incompatible with life to mild cases of patients diagnosed as adults (2, 21, 23–27). GD001 is a 4-year-old boy with a prenatal history of polyhydramnios, delivered at term with short stature (40.7 cm, 0.1th centile) at birth. After delivery, he was diagnosed with a ventricular septal defect along with mild pulmonary valve stenosis and patent ductus arteriosus, which subsequently evolved to mild pulmonary stenosis only. Clinical examination at 1 year old showed short stature, brachydactyly, and bilateral fifth finger clinodactyly. At age 4, his height, weight, and head circumference read 98 cm (6th centile), 15.6 kg (24th centile), and 49 cm (19th centile), respectively. His continuing findings are tip-toe walking with short fingers and toes. He has thickened ear helices and slightly underdeveloped alae nasi. A cardiac echo shows mild pulmonary stenosis. Exome sequencing revealed that he was heterozygous for the c.182G>A (p.R61H) and c.493G>A (p.A165T) variants in *ADAMTSL2* (Supplemental Table 1; supplemental material available online with this article; https://doi.org/10.1172/jci.insight.174417DS1). Both variants were interpreted as variants of uncertain significance according to American College of Medical Genetics (ACMG) guidelines (28). The mother is asymptomatic and heterozygous for c.182G>A. The father is asymptomatic and wild-type (WT) for *ADAMTSL2*. Long-read genome sequencing via Oxford Nanopore PromethION 24 sequencing system showed that the c.493G>A variant was on the paternal allele, verifying compound heterozygosity of the proband.

### ADAMTSL2 mutants impair protein secretion.

Previous reports have shown that some *ADAMTSL2* variants may impair the secretion of the protein (5, 10, 11). This reduced secretion might be key to understanding the disease mechanism.

**Figure 1:**
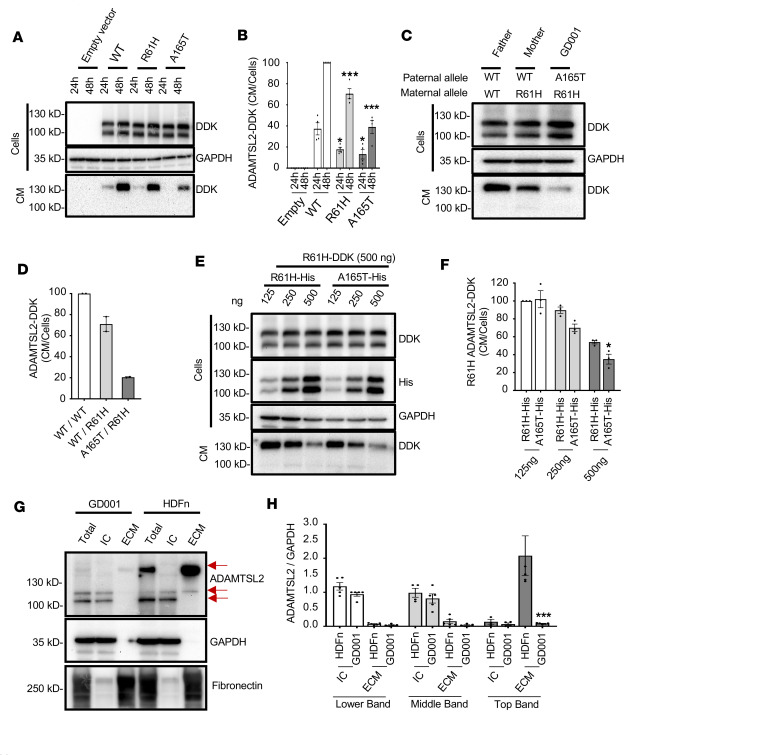
Mutant ADAMTSL2 secretion is impaired, and p.A165T causes a more severe impairment than p.R61H. (**A**) ADAMTSL2 is detected in cell lysates and the conditioned media (CM) of HEK293T cells at 24–48 hours after transfection with p.R61H, p.A165T, or wild-type (WT) ADAMTSL2-DDK. (**B**) Densitometry analysis of the CM/cells ratio shows a reduction of ADAMTSL2-DDK in the extracellular space when it is mutated. (**C**) Secretion of ADAMTSL2 in the CM is further reduced when 2 mutations are cotransfected in HEK293T cells 48 hours after transfection. (**D**) Densitometry analysis of the CM/cells ratio shows a further reduction of ADAMTSL2-DDK in the extracellular space when 2 mutants are cotransfected. (**E**) ADAMTSL2 is detected in the CM of HEK293T cells 48 hours after transfection with p.R61H-ADAMTSL2-DDK cotransfected with increased doses of either p.R61H- or p.A165T-ADAMTSL2-His. (**F**) Densitometry analysis of the CM/cells ratio shows a further reduction of p.R61HADAMTSL2-DDK in the extracellular space when cotransfected with p.A165T-ADAMTSL2-His. (**G**) ADAMTSL2 secretion is impaired in GD1 fibroblasts. Red arrows show ADAMTSL2 in the total lysate and intracellular and extracellular compartment. Glycosylation and fucosylation of ADAMTSL2 likely cause the higher molecular weight bands detected in the ECM. GAPDH and fibronectin were used as cytoplasmic and ECM markers, respectively. (**H**) Densitometry analysis of the ADAMTSL2/GAPDH ratio shows a significant reduction of secreted ADAMTSL2 in the extracellular space of GD1 dermal fibroblasts compared with human dermal control fibroblasts. (**A**–**H**) The ECM/intracellular (IC) ratio was normalized with the intensity of the CM from WT sample 48 hours after transfection. Data shown as mean ± SEM. (*, *P* < 0.05; ***, *P* < 0.001, 2-tailed *t* test.) All densitometry analyses were done with at least 3 independent experiments.

To study if p.R61H or p.A165T ADAMTSL2 decreases the secretion of the mutated protein, we constructed *ADAMTSL2* plasmid vectors with fusion tag DYKDDDDK (DDK). Transient expression experiments in HEK293T cells were used to quantify the relative amount of ADAMTSL2-DDK secreted into the medium relative to the total amount expressed within the cell (Figure 1, A and B). Results showed that ADAMTSL2-DDK was secreted in the conditioned medium, but mutated p.R61H ADAMTSL2 or p.A165T ADAMTSL2 showed a reduced secretion despite similar levels of intracellular expression as the WT protein. Overall, p.R61H ADAMTSL2 secretion was around 70% of WT secretion. In contrast, p.A165T ADAMTSL2 secretion was merely approximately 40% of WT secretion.

Because our proband has both mutated proteins (p.R61H and p.A165T) (Figure 1, C and D), we then examined if expressing both mutant proteins simultaneously affects the secretion of the individual mutants. For this purpose, DDK-tagged p.R61H ADAMTSL2 was cotransfected with different amounts of His-tagged p.R61H ADAMTSL2 or His-tagged p.A165T ADAMTSL2. The results showed a reduced secretion of p.R61H-DDK when cotransfected with p.A165T ADAMTSL2-His compared with cotransfection of p.R61H ADAMTSL2-His (Figure 1, E and F). This suggests that the presence of p.A165T further impairs the secretion of p.R61H ADAMTSL2. Taken together, we show that variants can have different effects on secretion and that a mutant protein can negatively impact the secretion of another mutant protein. This negative impact could be the result of aberrant dimer formation of mutant proteins. In a previous study for the p.R221C ADAMTSL2 variant in beagles, it has been reported that the mutation causes an aberrant dimerization of ADAMTSL2 (29). With the p.R221C variant, the dimerization was expected because the thiol group in cysteine can facilitate dimerization. While the p.R61H and p.A165T ADAMTSL2 variants are not expected to facilitate dimerization, we examined the cell lysates and conditioned medium of HEK293T cells after transient transfection. The cells were transfected with p. A165T ADAMTSL2-DDK and/or with p.R61H ADAMTSL2-DDK and compared with WT ADAMTSL2-DDK transfections in reduced and nonreduced conditions, for both intracellular and secreted proteins (Supplemental Figure 1S1, A and B). We do not detect abnormal dimerization when the images of mutant samples are compared to those of control.

To further study the molecular consequences of mutant ADAMTSL2, primary dermal fibroblasts from the proband were cultured. We compared these cells with primary dermal fibroblasts from asymptomatic individuals (Supplemental Table 1). Primary fibroblasts were cultured for 10 days, and cell lysates and extracellular matrix were collected separately, quantified, and visualized with immunoblot. Results show that the extracellular matrix of GD1 contains a reduced amount of ADAMTSL2 when compared with the control fibroblasts (Figure 1, G and H). Three bands were detected above 100 kDa by a polyclonal antibody against the C-terminus of ADAMTSL2. Multiple bands were expected since the protein is glycosylated for its secretion (8, 10, 12). The lower band is attributed to be the unmodified and nonsecreted protein; the middle and top bands are likely to be the result of posttranslational modifications in the secreted form. Within the extracellular matrix, ADAMTSL2 has been reported to interact with other extracellular proteins, such as FBN1, LOX, fibronectin, other ADAMTS, and LTBP1 (5, 12, 15, 30). In proband fibroblasts we found not only a reduction of ADAMTSL2 accumulation in the extracellular matrix but also a reduction in the expression of extracellular matrix proteins FBN1 and collagen type IIIα1 (Supplemental Figure 1S2, A and B). In conclusion, if reduced secretion is the mechanism of the disease, the p.A165T variant is expected to result in a more severe phenotype than p.R61H, since the latter has more secretion.

Previous studies have shown that ADAMTSL2 dysfunction could be involved in upregulating TGF-β bioavailability (5, 6, 11, 12, 19, 31). In all these reports TGF-β was quantified with an enzyme-linked immunosorbent assay (ELISA) specific for TGF-β1. TGF-β1 was the first member identified and the prototype of a large family of growth factors encoded by 33 genes in mammals (32, 33). We used ELISA to determine if p.A165T or p.R61H ADAMTSL2 increases TGF-β1 bioavailability after transient expression experiments in HEK293T cells. We also determined the phosphorylation of Smad2, which is a downstream target of TGF-β1, TGF-β2, TGF-β3, and activins. Results showed that neither p.A165T nor p.R61H ADAMTSL2 increased the amount of TGF-β1 or the phosphorylation of TGF-β downstream target Smad2 (Supplemental Figure 1S3, A–C). In addition, in the probandʼs primary fibroblasts, total TGF-β1 was reduced without any significant difference in the active TGF-β1 or in the phosphorylation of TGF-β downstream targets Smad2 and Smad3 (Supplemental Figure 1S3, D–F). The total amounts of Smad2 and Smad3 were not different, but Smad4 was reduced in the probandʼs fibroblasts compared with control cells (Supplemental Figure 1S3, E and F). To validate the integrity of the pathway in these cells, we added TGF-β1 and verified its activation with a significant increase in Smad2 phosphorylation (Supplemental Figure 1S3G).

Recently, ADAMTSL2 has been shown to be involved in the increase of Wnt signaling in myoblasts (34). To determine if the probandʼs fibroblasts have reduced Wnt signaling, we analyzed components of this pathway via immunoblots. We expected that canonical Wnt signaling would result in phosphorylation and inactivation of glycogen synthase kinase-3β (GSK3β) and accumulation of β-catenin in the probandʼs fibroblasts. The experiments showed that the probandʼs cells did not have significant changes in the target proteins when compared to control cells (Supplemental Figure 1S4, A and B). In the noncanonical Wnt/calcium signaling pathway, we would expect an increase in calmodulin-dependent protein kinase II (CaMKII) phosphorylation in the patient’s fibroblasts. Our results showed no difference in CaMKII phosphorylation when compared to control primary fibroblasts (Supplemental Figure 1S4, A and B). We further examined the basal activities of MERK/ERK and PI3K/AKT pathways in the probandʼs fibroblasts, which did not show a difference in the phosphorylation of ERK1/2 but showed a significant reduction in serine 473 AKT phosphorylation when compared with control fibroblasts (Supplemental Figure 1S4, A and B).

### Degrees of phenotypic severity in mice carrying different mutated alleles of ADAMTSL2.

To have a better understanding of the GD1 phenotype, we developed 2 knockin mouse models carrying the *Adamtsl2* missense variants p.R61H and p.A165T (Supplemental Figure 2S1A). Different crossing schemes were set up between the 2 knockin mice and with the *Adamtsl2*-KO mice (Supplemental Figure 2S1, A–C). We confirmed with real-time PCR that expression of *Adamtsl2* in KO mice was abrogated after the gene trapping cassette as previously reported (Supplemental Figure 2S1C) (12). We also determined the LacZ expression under *Adamtsl2* promoter in several tissues in newborn mice (Supplemental Figure 2S2). With these crossings, we obtained compound-heterozygous mice to model the patient GD001 with the 2 *ADAMTSL2* missense variants (p.R61H and p.A165T). In addition, homozygous, hemizygous, or heterozygous mice for the different alleles provided a unique set of combinations. This animal model collection was used to determine the specific contribution of the individual alleles to the disease and to better dissect the genotype-phenotype outcomes. We systematically characterized the mutant animals, focusing on the patient’s clinical presentation. Survival and growth curves (from 1 to 126 days old), anatomical measurements (2 months old), echocardiogram (at 3 and 5 months old), whole-body plethysmography (3 and 5 months old), micro-computed tomography (6 months old), and MRI (6 months old) were performed. p.R61H and p.A165T compound-heterozygous knockin mice survived after birth and recapitulated the distinct phenotype observed in patients with GD1, including specific craniofacial features and skeletal abnormalities. Phenotypic analysis of mice with different allelic combinations in *Adamtsl2* clearly showed that the severity depended on the mutations and correlated with the severity of secretion impairment observed in vitro.

**Figure 2:**
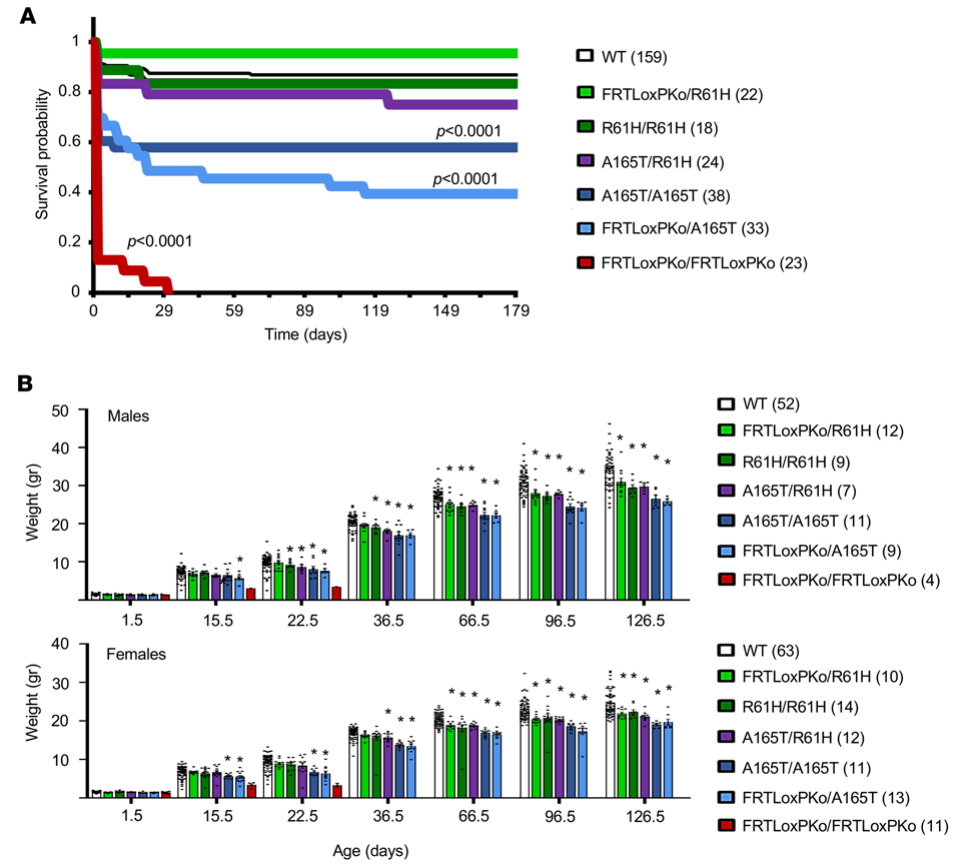
Survival and growth of mutant mice associated with *Adamtsl2* allelic combinations. KO animals die shortly after birth. The p.A165T allele has a more severe impact on survival and growth than the p.R61H allele. (**A**) Kaplan-Meier survival curve of mice with different allelic combinations in *Adamtsl2* during the first 6 months. The curves were compared with the log-rank (Mantel-Cox) test to determine significance. A significant reduction of survival (*P* < 0.0001) during the first 6 months was found in FRTLoxPKo/A165T, A165T/A165T, and KO animals. (*N* = number of animals.) (**B**) Growth curve male (top) and female (bottom). Average ± SEM. (*, *P* < 0.05, 2-tailed *t* test.)

We determined survival during the first 6 months (Figure 2A and Supplemental Figure 2S3A). Knockout mice, as expected, did not survive after birth except for a couple of outliers (12). Mice that were homozygous or hemizygous for p.A165T showed compromised survival compared with those that were homozygous or hemizygous for p.R61H or compound heterozygous for p.R61H and p.A165T variants. Hemizygous mice for p.A165T showed decreased survival when compared with homozygous p.A165T. In summary, a spectrum of severity was observed starting with an increased newborn lethality for the KO, to the medium severity of the p.A165T allele producing a significant phenotype but with better survival than KO, and the mild p.R61H that gave rise to a less severe form of the disease (Figure 2A and Supplemental Figure 2S3A).

We also monitored the growth of the mice by measuring their weight gain (Figure 2B and Supplemental Figure 2S3B). On the first day after birth, there was no significant weight difference among all mice. One KO male mouse survived for 30 days and was significantly smaller and underweight at 3 weeks of age. Mice that were homozygous or hemizygous for p.A165T showed reduced weight gain starting from 3 to 4 weeks of age when compared with WT. Mice homozygous or hemizygous for p.R61H showed reduced weight gain only after 2 to 3 months of age. Compound p.R61H and p.A165T heterozygous mice were also significantly underweight after 1 month of age. All heterozygous mice had similar weight gains relative to WT.

### The severity of craniofacial and skeletal abnormalities is correlated with allelic combination.

**Figure 3:**
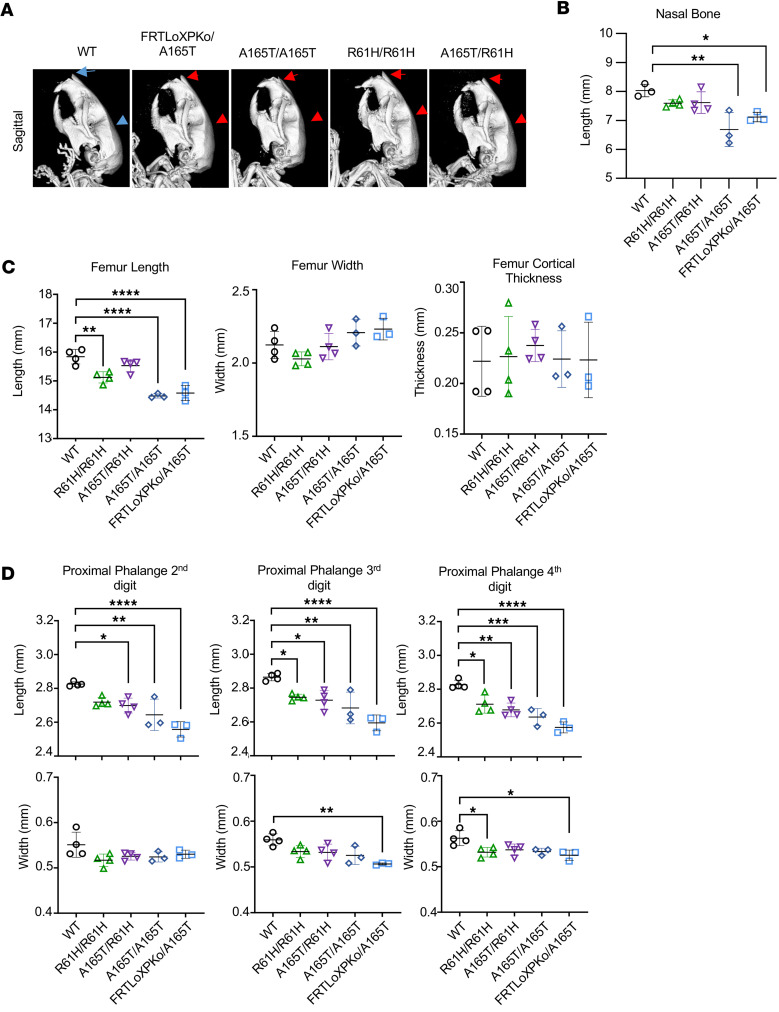
Skeletal abnormalities in *Adamtsl2*-mutant mice detected with CT scans. Bone morphology differences in mice with the p.A165T mutation were more severe than in mice with the p.R61H variant. (**A**) Cranial morphology in animals with mutated *Adamtsl2*. A more rounded skull and shorter nasal bone are observed in 3D reconstruction of CT scans from mice with mutated *Adamtsl2*. (**B**) Shorter nasal bone, (**C**) shorter femur, and (**D**) shorter proximal phalanges are found in animals with mutated *Adamtsl2*. Data shown as mean ± SD. (*, *P* < 0.05; **, *P* < 0.01; ***, *P* < 0.001; ****, *P* < 0.0001, 1-way ANOVA.) CT scan done in 4 WT, 4 R61H/R61H, 4 A165T/R61H, 3 A165T/A65T, and 3 FRTLoxPKo/A165T. All animals analyzed were males at 6 months old.

Patients with GD have distinctive facial features giving the impression of a “happy” face (3, 4). The name geleophysic is a combination of the Greek words *Gelios*, which means “happy,” and *Physis*, which means “nature.” These patients have full cheeks, shortened nose, hypertelorism, long and flat philtrum, and thin upper lip. While these characteristics are described in humans, our initial observations of the adult animals suggested that there were head shape differences between distinct allelic combinations. Specifically, the snout looked shorter in hemizygous and homozygous p.A165T (Supplemental Figure 3S1A). To determine if there are morphological differences, we performed CT scans that showed that the cranium was more rounded in mice with either hemizygous or homozygous *Adamtsl2* p.A165T or p.R61H (Figure 3A and Supplemental Figure 3S1A). We found that the nasal bone was significantly shorter in animals that were homozygous or hemizygous for p.A165T when compared with age-matched controls (Figure 3B and Supplemental Figure 3S1B). Furthermore, patients with GD are characterized by short stature and short hands and feet (3, 4). In mice, we found that the femur length was shortened in animals with p.A165T and p.R61H variants, but no difference was found in width or cortical thickness (Figure 3C and Supplemental Figure 3S1B). Radiographic images of patientsʼ hands have shown short and wide phalanges (1, 4, 5, 25, 35). In mice, we saw similar findings in measurements of proximal phalanges of second, third, and fourth digits from front paws (Figure 3D and Supplemental Figure 3S1B). Proximal phalanges were the shortest in the p.A165T hemizygous mice followed by p.A165T homozygotes, compound heterozygous p.A165T and p.R61H, as well as p.R61H hemizygous and homozygous mice (Figure 3D). The mice did not show a width increase of the phalanges. There was a width reduction in the third and fourth proximal phalanges in p.A165T hemizygotes.

### The spectrum of respiratory insufficiency and cardiac abnormalities correlates with specific allelic combinations.

**Figure 4:**
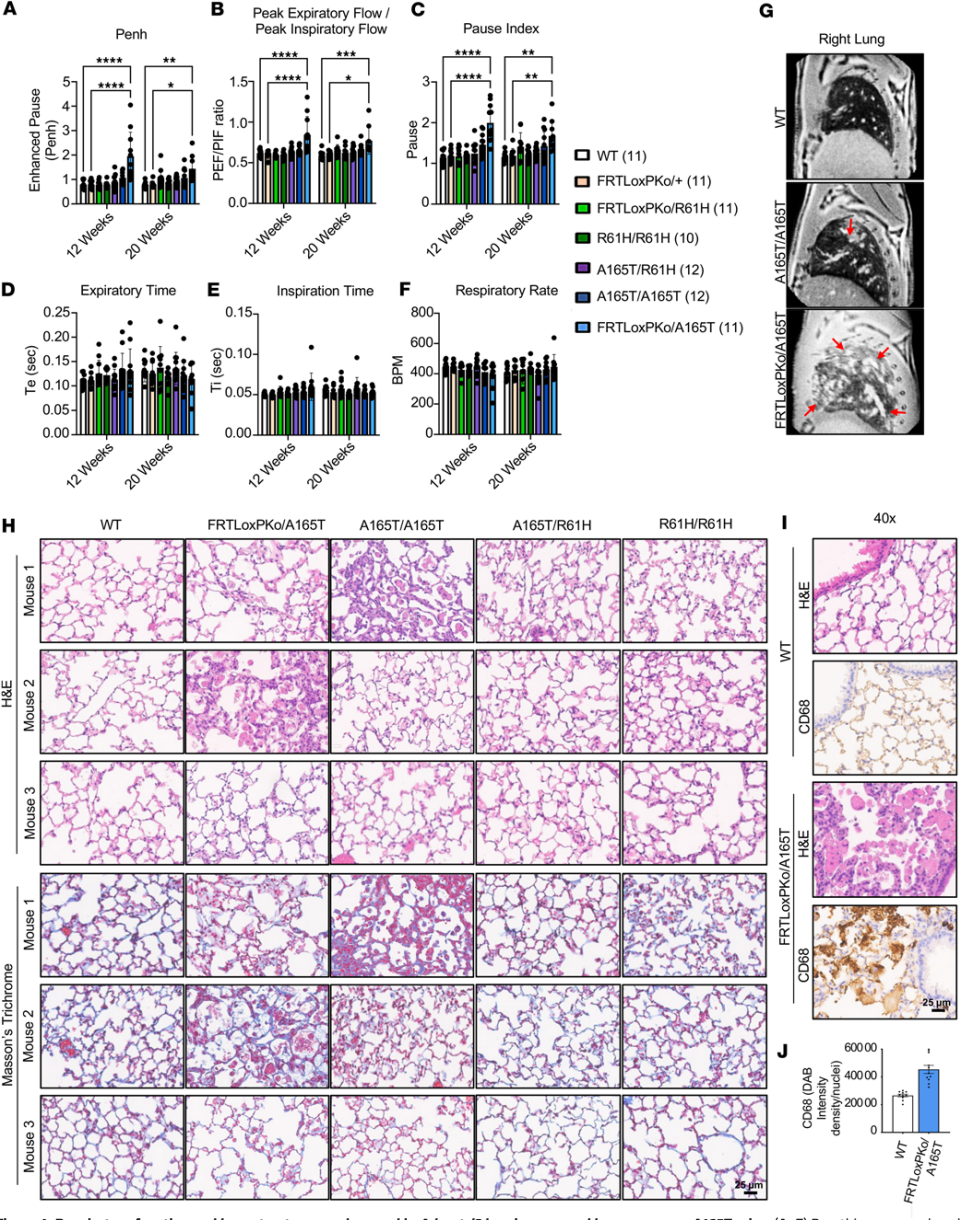
Respiratory function and lung structure are abnormal in *Adamtsl2* hemizygous and homozygous p.A165T mice. (**A**–**F**) Breathing was analyzed with whole-body plethysmography at 12 and 20 weeks of age in male and female mice. Indicators of expiration are significantly affected in mice with the p.A165T mutation. Data shown as mean ± SD (*N* = number of animals) (*, *P* < 0.05, **, *P* < 0.01, ***, *P* < 0.001, ****, *P* < 0.0001 2-way ANOVA). (**G**) MRI scans show ground glass opacifications and consolidations (arrows) in the lungs of A165T/A165T (mouse 1) and FRTLoxPKo/A165T (mouse 2). These animals had the most severe respiratory abnormalities in the plethysmography. MRI was performed in 4 WT, 4 R61H/R61H, 4 R61H/A165T, 5 A165T/A165T, and 3 FRTLoxPKo/A165T in 6-month male mice. (**H** and **I**) Histology from 3 WT, 3 FRTLoxPKo/A165T, 3 A165T/A165T, 3 A165T/R61H, and 3 R61H/R61H in 7-month male mice. (**H**) Microscopic post-obstructive pneumonia was found in areas of the lungs of FRTLoxPKo/A165T mouse 2 and of the lungs of A165T/A165T mouse 1. (**I** and **J**) Staining with CD68 macrophage marker shows large foamy macrophages filling alveolar airspaces, indicating microscopic post-obstructive pneumonia in the mouse whose MRI is shown in **G**. BPM, breaths per minute.

Respiratory insufficiency is another finding in some patients with GD (2, 23). Respiratory function analysis of mice with different allelic combinations in *Adamtsl2* was conducted using whole-body plethysmography (WBP) at 12 weeks and 20 weeks of age. This test revealed dysfunction in the expiration phase in some animals (Figure 4, A–F and Supplemental Figure 4S1). Indicators of the expiration phase, including enhanced pause (Penh), pause index, and the peak expiratory flow/peak inspiratory flow ratio (PEF/PIF), were significantly affected in p.A165T hemizygous and homozygous mice (Figure 4, A–C). The inspiration phase and other parameters of respiration, including respiratory rate and tidal volume, were not significantly altered (Figure 4, E and F, and Supplemental Figure 4S1). The severity of respiratory insufficiency appeared to vary among animals with the same mutation, suggesting that additional factors could contribute to the severity of respiratory dysfunction. It is noteworthy that male mice had a more severe respiratory dysfunction than female mice. Additionally, we did not observe a significant progression of respiratory dysfunction between 12 and 20 weeks. The expiratory dysfunction was observed only in some animals harboring the p.A165T allele (homozygous and hemizygous). Hemizygous mice showed a more severe dysfunction when compared with homozygous mice. Some of these animals tested at 12 weeks did not survive long enough for the 20-week test. An MRI scan was performed in 3 animals of each group to better characterize the respiratory dysfunction detected via WBP (Figure 4G and Supplemental Figure 4S2). Bilateral ground glass opacification with multifocal areas of poorly defined consolidation were observed in the lungs of 2 of the most severe cases (p.A165T hemizygous and p.A165T homozygous). Masson’s trichrome staining is particularly useful in visualizing connective tissue as well as pathologic collagen fibrosis. We did not see significant pulmonary fibrosis in any of the animal models (Figure 4H). We detected a slight increase in fibrous connective tissue around some airways but not beyond normal histologic limits. We noted evidence of microscopic post-obstructive pneumonia, characterized by the accumulation of foamy macrophages (i.e., endogenous lipid-laden macrophages) within alveolar airspaces, in the most severely affected animals (p.A165T hemizygote and p.A165T homozygote), which correlated with the respiratory dysfunction detected in the MRI and WBP tests (Figure 4, G–J). Microscopic post-obstructive pneumonia is indicative of airway obstruction, either physical or functional, limiting the rate at which alveolar macrophages are cleared from air spaces.

**Figure 5:**
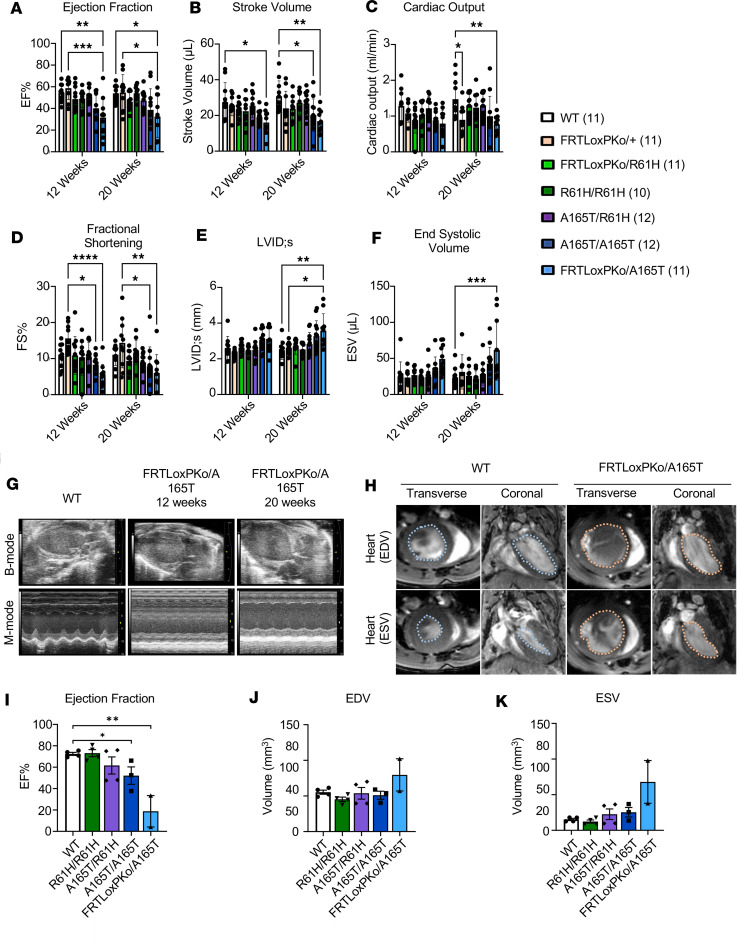
Heart function and structure are abnormal in *Adamtsl2* hemizygous and homozygous p.A165T mice. (**A**–**F**) Cardiac function was analyzed with an echocardiogram at 12 and 20 weeks of age in male and female mice. Indicators of systolic function are significantly affected in mice with the p.A165T mutation. (LVID;s = left ventricular end-systolic internal diameter) (*N* = number of animals) (*, *P* < 0.05; **, *P* < 0.01; ***, *P* < 0.001; ****, *P* < 0.0001, 2-way ANOVA). (**G**) Prominent narrowing of the aortic root is observed in the echocardiogram of all FRTLoxPKo/A165T mice. (**H**) MRI scans show a deficit in the systolic function and EF. (**I**–**K**) Transverse sections were used to calculate the EF, end-diastolic volume (EDV), and ESV. MRI scan was done in 6-month male mice. 4 WT, 4 R61H/R61H, 4 A165T/R61H, 3 A165T/A65T, and 2 FRTLoxPKo/A165T. (*, *P* < 0.05*;* **, *P* < 0.01, 1-way ANOVA.)

**Figure 6:**
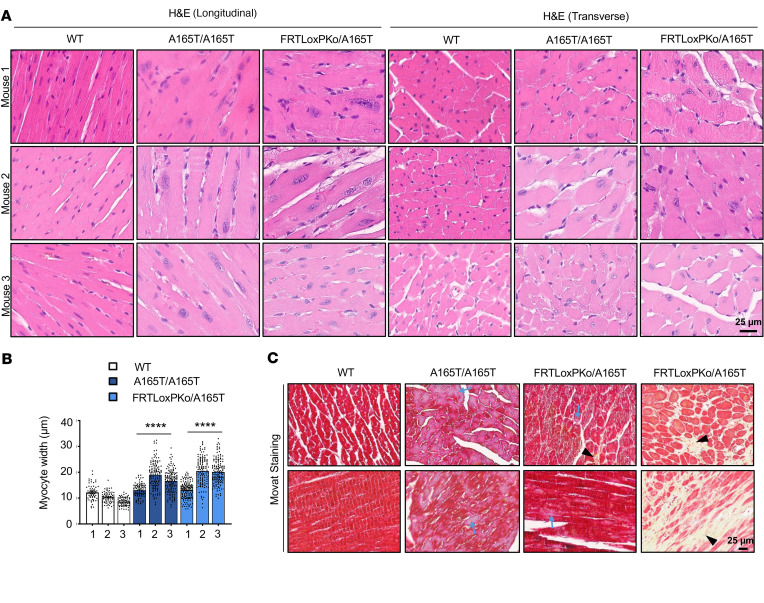
Heart histology reveals hypertrophic cardiomyopathy, myocyte hypertrophy, and small areas of fibrosis in mice with *Adamtsl2* p.A165T mutation. Serial histology staining of the whole heart of 3 WT, 3 A165T/A165T, and 3 FRTLoxPKo/A165T. (**A**) The hearts with p.A165T homozygous or hemizygous mutation had hypertrophic cardiomyopathy with myocyte hypertrophy in H&E staining. (**B**) Myocyte hypertrophy in p.A165T homozygous or hemizygous mice was validated with myocyte width quantification when compared with WT mice. (****, *P* < 0.0001, 2-way ANOVA.) (**C**) Movat staining validates that in *Adamtsl2* hemizygous and homozygous p.A165T mice with a severe phenotype, the muscle tissue in the ventricles has areas of mild interstitial fibrosis. In Movat, areas with yellow staining indicate mild interstitial fibrosis due to the high collagen content (black arrowhead). Some cardiomyocytes in the ventricles also show areas with patchy blue staining (blue arrows). All the animals were 7 months of age and male.

**Figure 7:**
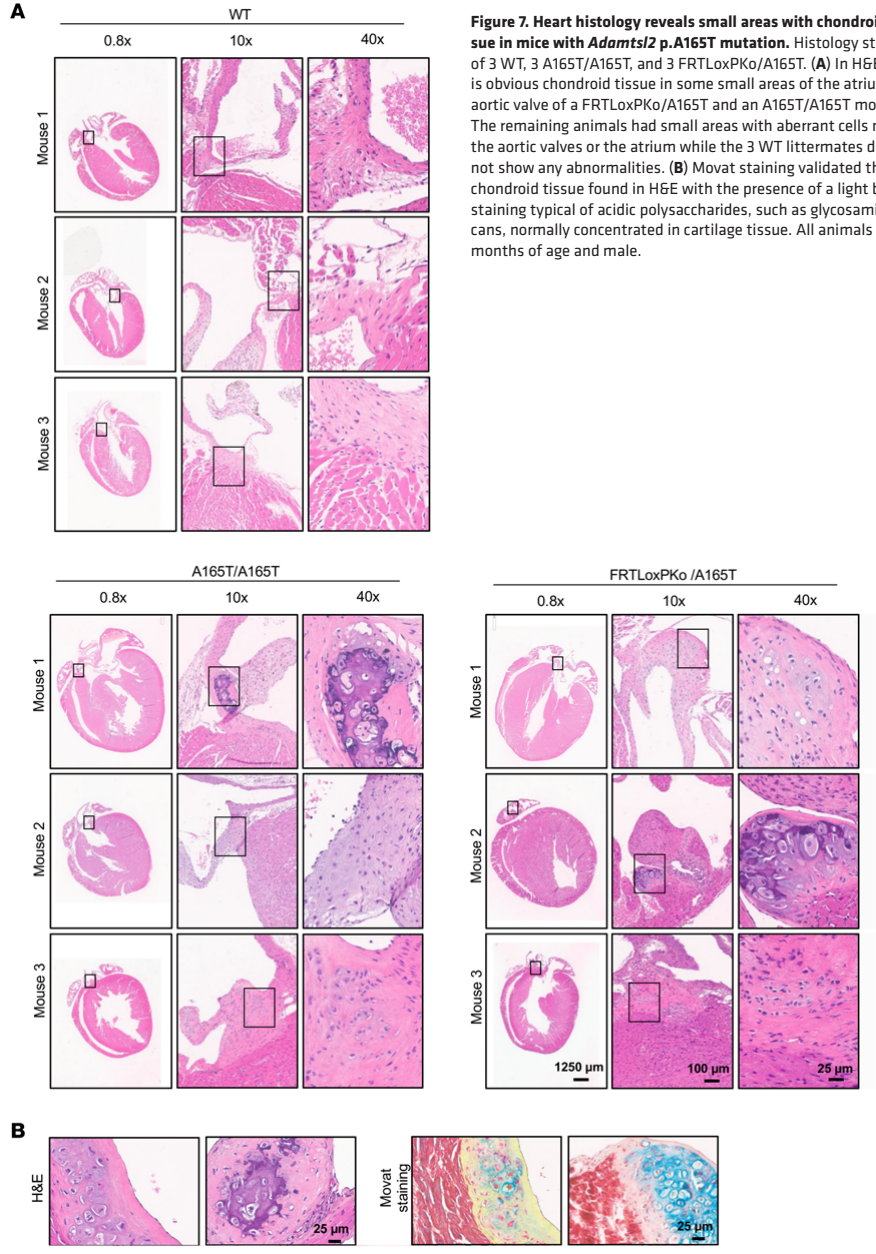
Heart histology reveals small areas with chondroid tissue in mice with *Adamtsl2* p.A165T mutation. Histology staining of 3 WT, 3 A165T/A165T, and 3 FRTLoxPKo/A165T. (**A**) In H&E there is obvious chondroid tissue in some small areas of the atrium and aortic valve of a FRTLoxPKo/A165T and an A165T/A165T mouse. The remaining animals had small areas with aberrant cells near the aortic valves or the atrium while the 3 WT littermates did not show any abnormalities. (**B**) Movat staining validated the chondroid tissue found in H&E with the presence of a light blue staining typical of acidic polysaccharides, such as glycosaminoglycans, normally concentrated in cartilage tissue. All animals were 7 months of age and male.

In GD, cardiac valvular dysfunction is a major concern impacting morbidity and mortality (2, 23, 25, 36). Echocardiograms of the *Adamtsl2* p.A165T hemizygote or homozygotes showed significantly reduced ejection fraction (EF%), reduced stroke volume, reduced cardiac output, reduced fractional shortening (FS%), increased left ventricular end-systolic internal diameter (LVID;s) and increased end-systolic volume (ESV) (Figure 5, A–F). The findings in the echocardiograms suggest profound systolic dysfunction, in hemizygous and homozygous p.A165T mice, indicative of inadequate ejection of blood from the heart chambers following each systolic contraction. Also, this systolic dysfunction was progressive as the LVID and ESV were significantly increased at 20 weeks. The ratio of peak mitral inflow velocity during early diastole and mitral annular velocity was altered, indicating increased ventricular filling pressure in these animals (Supplemental Figure 5S1, A–F). However, other parameters that indicate diastolic dysfunction, such as isovolumic relaxation time and mitral ratio of the early to late ventricular filling velocities**,** were not significantly altered. There was an increase in the left ventricular end-systolic anterior wall thickness at 12 weeks of age when compared with WT animals (Supplemental Figure 5S1, G–L). The pulmonary ejection time is significantly reduced at 20 weeks in hemizygous p.A165T mice indicating that there is increased afterload in the right ventricle (Supplemental Figure 5S2, A–C). Nevertheless, pulmonary acceleration time (PAT) and PAT/pulmonary ejection time, additional parameters that correlate with pulmonary artery systolic pressure, were not significantly altered. The mean pulmonary arterial pressure was not significantly changed in all the animals, but the pulmonary valve peak pressure was increased in hemizygous p.A165T mice (Supplemental Figure 5S2, D–F). When the aortic function was evaluated, there was large variability of the aortic valve peak pressure and the aortic ejection time (Supplemental Figure 5S2, G–I). A prominent narrowing of the aortic root was also observed in all the hemizygous p.A165T mice (Figure 5G). We quantified the aortic root opening by calculating the aortic valve area, which showed a significant reduction in hemizygous p.A165T mice (Supplemental Figure 5S3, A and B). These data indicate that the mutant mice had cardiac insufficiency and narrowing of the aortic and possibly pulmonary valve openings (Supplemental Table 2). MRI scans showed that the hearts of hemizygous and homozygous p.A165T mice were unable to pump all the blood (Figure 5, H–K; Supplemental Figure 5S2J; and Supplemental Video 1). End-systolic images showed that end-systolic ventricles were still full of blood (Figure 5, H and K; Supplemental Figure 5S2J; and Supplemental Video 1). Like the echocardiogram results, the EF calculated from the MRI indicated that p.A165T hemizygous mice had a more severe cardiac phenotype followed by the homozygous p.A165T mice. Hemizygous p.R61H and homozygous p.R61H mice and compound-heterozygous p.A165T and p.R61H mice did not show any significant cardiac pathology when compared to the WT mice. To determine the nature of damage to the heart and rule out valve fibrosis, serial histology sections of the whole heart were performed in 3 WT, 3 p.A165T homozygous, and 3 p.A165T hemizygous animals (Figure 6, A–C; Figure 7, A and B; Supplemental Figure 6S1; and Supplemental Figure 7S1). Routine H&E stains showed that p.A165T hemizygous and homozygous hearts had hypertrophic cardiomyopathy along with myocyte hypertrophy by different degrees of severity among animals with the same mutation, suggesting that additional factors contributed to the severity of cardiac dysfunction (Figure 6, A and B). With Movat staining there was patchy mild interstitial fibrosis in the most severe p.A165T hemizygous and homozygous animals according to the cardiovascular dysfunction found via the echocardiogram and the MRI (Figure 6C). The fibrosis was verified with Picrosirius Red staining Supplemental Figure 6S2). Upon H&E staining, some of these animals had histologic evidence of small areas with obvious chondroid metaplasia localized mainly in the aortic valve and the atrium (Figure 7, A and B; Supplemental Figure 6S1; and Supplemental Figure 7S1). Close examination of the valves showed that all p.A165T hemizygous and homozygous mice had small areas with abnormal cells in the aortic valves and atrium. These cells showed chondroid differentiation characteristics and were different from other areas of the same heart and WT animals (Figure 7, A and B, and Supplemental Figure 7S1). Movat stains verified the chondroid tissue found in H&E with a light blue staining typical of glycosaminoglycans normally concentrated in cartilage (Figure 7B). In the ventricles, some cardiomyocytes in *Adamtsl2* hemizygous and homozygous p.A165T mice showed a distinct patchy blue staining (Figure 6C).

A summary of the cardiac dysfunction is provided in Supplemental Table 2.

### Phenotypic severity in mutant mice correlates with a reduced amount of mutated Adamtsl2.

**Figure 8:**
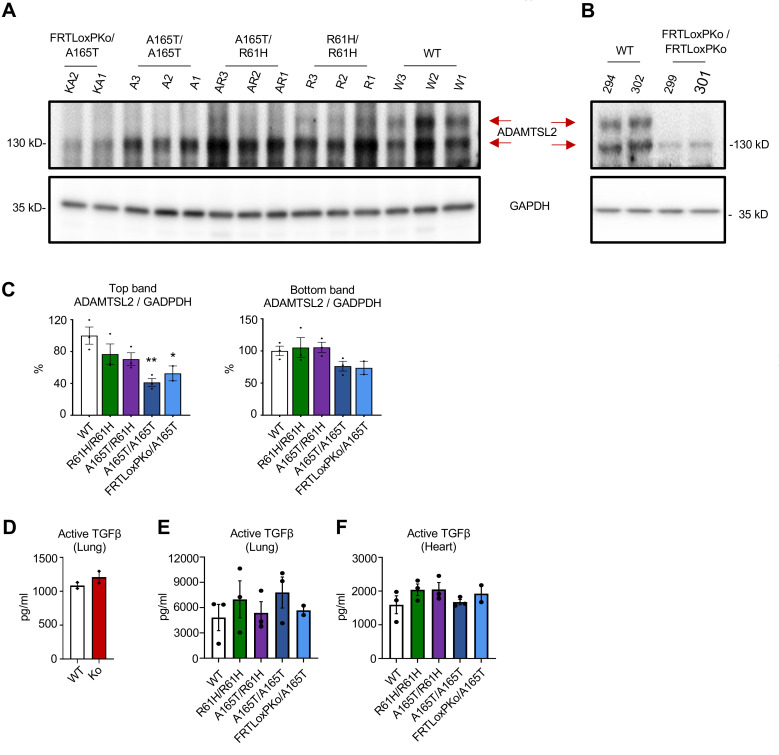
Mutant ADAMTSL2 secretion is impaired in vivo. (**A**) ADAMTSL2 is detected in the total lysate from 8-month-old female mouse lungs. A band above 130 kDa is reduced in mutant ADAMTSL2. (**B**) Lung tissue from newborn KO mice (FRTLoxPKo / FRTLoxPKo) does not have a top band above 130 kDa. There is a bottom band of around 130 kDa that is substantially reduced in these animals. Red arrows mark the bands that correspond to the bands absent in KO mouse immunoblots and appear to be ADAMTSL2. Posttranslational modification of ADAMTSL2 can result in higher molecular weight. (**C**) Densitometry analysis of the top and bottom bands of ADAMTSL2/GAPDH ratio shows a reduction of ADAMTSL2 in mutant animals. (*, *P* < 0.05; **, *P* < 0.01, 1-way ANOVA.) (**D**–**F**) Active TGF-β measurement by ELISA in mouse tissues. (**D**) There is no significant increase of active TGF-β in the lung tissue of newborn KO animals compared to littermate WT. A 2-tailed *t* test was used to test significance. There is no significant increase of active TGF-β in adult mutant animals when compared to WT in the lung (**E**) or heart tissues (**F**). Data shown as mean ± SEM. One-way ANOVA was used to test significance.

Via in vitro experiments, we found that p.A165T or p.R61H ADAMTSL2 had decreased secretion. This finding predicts that there could be a reduced abundance of mutated protein in vivo. To quantify the mutated Adamtsl2, we collected and compared total protein lysates from the lungs of adult WT, homozygous p.R61H, compound heterozygous p.A165T/p.R61H, homozygous p.A165T, and hemizygous p.A165T mice. Consistent with previous publications and our work (Figure 1, A–H), we found that Adamtsl2 immunoblots of WT animals had multiple bands (Figure 8, A–C). Previous work has suggested that a secreted glycosylated Adamtsl2 results in a band above 130 kDa (8, 10, 12). KO mice did not express this protein as expected (Figure 8B). We found a significant reduction in the Adamtsl2 band above 130 kDa in p.R61H homozygotes and that this reduction was further diminished in p.A165T/p.R61H, p.A165T homozygotes, and p.A165T hemizygotes (Figure 8, A–C). The graded decrease of Adamtsl2 correlated with the degrees of severity found in the phenotype of these mice.

ELISA quantification was performed to determine if the amount of mouse active TGF-β1 was affected in mutant Adamtsl2 animals. In previous studies, whereas TGF-β signaling has been found to be increased in primary dermal fibroblasts from patients and in the lung of embryonic E17 knockout mice, in the lung of newborn mice it is found to be at the same level as WT (12). Consistent with those results, we did not find a significant elevation of active TGF-β1 in KO newborn mice when compared to WT littermates (Figure 8D). In addition, we determined the level of TGF-β1 in the lung and heart of adult WT, homozygous p.R61H, compound-heterozygous p.A165T/p.R61H, homozygous p.A165T, and hemizygous p.A165T mice. The results showed no difference among the samples (Figure 8, E and F). Moreover, phosphorylation of TGF-β downstream target Smad2 was low in the heart and lung tissue of mutants (Supplemental Figures 8S1 and 8S2). Exploration of the signaling pathway activity in the lung tissue from adult animals showed a large variability in total protein levels and phosphorylation markers between heart samples (Supplemental Figure 8S1, A and B). Lung samples showed less variability in phosphorylation or total proteins, but no significant difference was found in TGF-β or Wnt signaling (Supplemental Figure 8S2, A and B).

## Discussion

Our study highlights the critical role of ADAMTSL2 in the extracellular matrix, a dynamic network crucial for cellular and tissue functions. Similar to some other *ADAMTSL2* variants, we showed that p.A165T and p.R61H variants reduce protein secretion, limiting their presence in the extracellular matrix (5, 11). While this impairment in secretion may lead to intracellular alterations, resembling lysosomal storage disorders (3, 4), it is the lack of functional ADAMTSL2 in the extracellular matrix that appears to be the primary mechanism underlying GD1.

Our in vitro experiments demonstrate that different *ADAMTSL2* variants exhibit varying levels of secretion impairment, with p.A165T causing more severe effects than p.R61H. Interestingly, the presence of 1 mutant allele can interfere with the secretion of another, leading to a variable phenotype when combinations of mutant alleles are present. The exact mechanism behind this interference remains unknown.

Human and animal model data indicate that the total absence of *ADAMTSL2* is incompatible with life, as seen in KO mice resembling the lethal phenotype observed in Al-Gazali skeletal dysplasia, the most severe form of GD1 (12, 21). In humans with GD, most of the described variants are missense, and no reported patient was homozygous or compound heterozygous for complete loss-of-function variants (e.g., nonsense or frameshift indel). This suggests that the variants retain some function, preventing a more severe outcome. It also likely explains the broad range of phenotypes observed, with the level of secretion playing a crucial role.

In our study, heterozygous mice, carrying 1 WT allele, did not exhibit any differences compared to WT mice, indicating that a single functional WT allele was sufficient to prevent the abnormal phenotype. However, in the absence of a WT allele, we observed a gradual increase in phenotypic severity among the combinations of variants. Mice homozygous for p.R61H, hemizygous for p.R61H, and heterozygous for p.R61H/p.A165T exhibited less severity compared with mice that were hemizygous or homozygous for p.A165T. This suggests that p.R61H still produces a functional mutated protein capable of mitigating the disease severity. Overall, our findings shed light on the different *ADAMTSL2* variants and their impact on diverse clinical manifestations of GD.

GD1 is categorized within the acromicric dysplasias, characterized by hypoplasia of the limbs, digits, nose, and jaws (2, 37). In our study, all mice with variant combinations exhibited some degree of skeletal abnormalities and growth deficit, which are consistent with the skeletal phenotypes observed in the acromicric dysplasia family of disorders. Similar skeletal abnormalities, such as brachydactyly and broad facial features, have been observed in dogs with an *ADAMTSL2* mutation and in the KO mouse model (19, 22, 38). The role ofADAMTSL2 in regulating chondrocyte function, proliferation, and differentiation has been reported, and specific deletion in these cells has been shown to cause dwarfism (19). Moreover, ADAMTSL2 function is vital in the composition of tendon microfibrils, and specific deletion in tendons can lead to skeletal growth impairment (22). Patients with GD also experience skeletal abnormalities, including early joint limitations, hip dysplasia, osteochondritis, and carpal tunnel syndrome, which significantly impact their well-being (2). The mouse models presented in our study offer a valuable platform to explore potential treatments for the skeletal morbidities observed in patients with GD1.

Our study reveals a notable association between the severity of *ADAMTSL2* allelic variants and respiratory insufficiency. Specifically, the most severe p.A165T variant exhibits clear dysfunction during the expiration phase. This finding is consistent with the clinical manifestations observed in patients with GD, who often experience recurrent respiratory infections and bronchopulmonary insufficiency (23). One possible explanation for the damage in the lungs is that the mutation in *ADAMTSL2* causes an increase in fibrosis. ADAMTSL2 has been linked to fibrosis in various contexts, including liver fibrosis in nonalcoholic fatty liver disease, cardiac fibrosis, and fibrosis in several organs in Musladin-Lueke syndrome (18, 29, 38, 39). However, we did not detect significant lung fibrosis in our mouse models. Instead, we identified microscopic post-obstructive pneumonia in the most severely affected animals. This finding aligns with previous observations in newborn mice lacking Adamtsl2, where obstruction-related changes, such as bronchial occlusion and bronchial dysplastic epithelium, were noted. These animals also exhibited glycogen accumulation in the bronchial epithelium and increased microfibrils in the bronchial wall starting at E16 (12).

Some patients with GD develop progressive and life-threatening cardiac disease, which can lead to early mortality (2). GD1 individuals may be affected by progressive cardiac valvular disease, involving thickening of the pulmonary, aortic, and mitral valves (5, 25). Our study shows a progressive cardiac dysfunction associated with the p.A165T variant, especially in p.A165T hemizygous mice, which exhibit a severe reduction in EF, stroke volume, and cardiac output as observed in echocardiograms. Parameters of systolic function, such as LVID and ESV, also indicated progressive systolic dysfunction. The narrowing of the aortic root is evident in these mice, and cardiac dysfunction is further verified through MRI. Histological analysis reveals hypertrophic cardiomyopathy and myocyte hypertrophy in severely affected animals. We observed interstitial fibrosis in some animals, but the absence of an increase in heart fibrosis in all Adamtsl2-mutant mice suggests that the fibrosis might be secondary to severe cardiac dysfunction rather than being the initial trigger. In addition, we observed intracellular accumulation of proteoglycans in individual myocytes in homozygous and hemizygous p.A165T mice. Notably, abnormal cells were found in areas of the atrium and aortic valve of all the p.A165T hemizygous and homozygous animals. Some of these cells exhibit chondrogenic characteristics, and the presence of chondroid nodules has been validated in the hearts of animals with the most severe cases, specifically those that are hemizygous or homozygous for the p.A165T mutation. A reduction in Wnt/β-catenin signaling has been linked to the formation of aberrant chondrogenic nodules in the heart. While ADAMTSL2 has been shown to promote myoblast differentiation through the Wnt signaling pathway (20, 40), we did not find a decrease in Wnt signaling in the heart tissue of mutant animals. Cartilaginous metaplasia has been observed in older patients with severe valve disease, suggesting a shared regulatory mechanism in the development of the heart, cartilage, and bone, where ADAMTSL2 may play a crucial role (41–45).

One interesting finding is that p.A165T and p.R61H mutations do not result in an increased TGF-β signaling in vitro or in vivo. Previous reports have shown an increased TGF-β signaling in patients with a few *ADAMTSL2* mutations (5, 6, 11, 12, 19, 31). We did not see an increased production of TGF-β1 or increased downstream signaling in primary dermal fibroblasts or the lung and heart tissues of mutant mice. There is a previous report indicating that newborn KO mice have the same level of TGF-β signaling as the control mice (12). In myoblast-derived cells with stable knockdown of *ADAMTSL2*, there is no increase in TGF-β activity (34). Finally, no change in TGF-β signaling in dermal fibroblasts from a patient with an *ADAMTSL2* mutation was also reported (21). Taken together, these results suggest either TGF-β signaling is not different in all *ADAMTSL2* mutations or there are other factors or conditions not fully defined to explain the observed discrepancy. The signaling changes observed in the tissue of the animals might be specific to these mutations, and the interpretation of these findings in other *ADAMTSL2* mutations may not be generalizable. Furthermore, future studies will determine the exact spatial location within the tissue and affected cells and if any of these are present early in development. There is a possibility that unique signaling changes are specifically pronounced during a short developmental window in younger animals.

In conclusion, our study offers a comprehensive evaluation of GD1 through a personalized research approach, enabling us to replicate the multisystemic nature of this disorder in an animal model. In addition, *Adamtsl2* allelic combinations closely resemble the full spectrum of phenotypic pathologies seen in patients. These novel animal models provide valuable insights into the underlying mechanisms of GD1 and serve as essential tools for advancing our understanding of the disease and exploring potential therapeutic interventions.

## Methods

### Sex as a biological variable.

Our study examined male and female animals, and sex-dimorphic effects were reported. The histology, CT scan, and MRI study were examined only in male mice to have less variability in phenotype. WBP and echocardiogram were done for male and female animals, and similar findings were reported for both sexes.

### Genome sequencing.

Genome sequencing was performed on both parents and the proband. Reads were mapped to human genome reference (National Center for Biotechnology Information build37/hg 19 version) with Burrows-Wheeler Aligner. The Genome Analysis Toolkit was used for variant calling. ACMG guidelines were used for the variant interpretation (28).

### Cell culture experiments.

Dermal fibroblasts were isolated from the patient’s skin biopsy, following the explant technique. Skin biopsy was cut into small explants and placed in a 6-well plate with complete Dulbecco’s modified Eagle medium (DMEM high glucose, pyruvate, 20% FBS, 100 U/mL of penicillin, 100 μg/mL of streptomycin, and 200 μg/mL of Primocin from InvivoGen). The medium was carefully changed every day until cells migrated from the explant and grew as a monolayer. After 50% confluence, cells were split to a T75 flask, and the medium was changed every 2–3 days until cells reached 70%–90% confluence. Dermal fibroblasts were cryopreserved as passage P2. Thawed fibroblasts for experiments were cultured in T75 flasks using DMEM high glucose, pyruvate, 10% FBS, 100 U/mL of penicillin, and 100 μg/mL of streptomycin.

HEK293T cells were purchased from the American Type Culture Collection. Cells were maintained with DMEM supplemented with 10% heat-inactivated FBS, 100 U/mL penicillin, and 100 μg/mL of streptomycin. Medium was changed every 2–3 days. HEK293T cells were transiently transfected using Lipofectamine 3000 (Invitrogen) following the manufacturer’s instructions. One million cells were cultured in 12-well plates and transfected after 24–48 hours with 70%–80% confluence. WT or mutated ADAMTSL2 constructs (all in pCMV6, Origene) (0.5 μg), P3000 reagent (Invitrogen) (2 μL), and Lipofectamine 3000 (4 μL) were mixed in 300 μL DMEM (no additives) and incubated at room temperature for 30 minutes. Cells were transfected using the DNA/lipid/DMEM mixture for 6 hours (500 μL total), and later 1 mL of DMEM high glucose, pyruvate, 10% FBS, 100 U/mL of penicillin, and 100 μg/mL of streptomycin were added. Cell lysates (RIPA buffer, MilliporeSigma) and conditioned media were obtained at 24, 48, and/or 72 hours for further analysis.

To obtain the total cell lysate (Total), intracellular cell lysates (IC), and extracellular matrix protein lysates (ECM), triplicate plates were cultured. For the collection, all the plates were washed with PBS. The Total sample was collected using 2× Laemmli/2ME. The IC sample was detached using 0.25% trypsin for 5 minutes at 37°C, then washed twice with PBS, and RIPA buffer was used to obtain the IC. The ECM was extracted using 20 mM NH_4_OH in TBS-Triton (0.05%) for 10 minutes at room temperature, and finally, proteins were extracted with 2× Laemmli buffer. Site-directed mutagenesis was performed to introduce specific changes in the ADAMTSL2 (NM_001145320) cDNA sequence initially cloned in the pCMV6 vector obtained from Origene. The mutagenesis was carried out using the Quick-Change Lightning Multi Site-Directed Mutagenesis Kit from Agilent Technologies. Variant ADAMTSL2: c.182G>A (p.R61H) and variant ADAMTSL2: c.493G>A (p.A165T) were generated using primers 5′ CCCCCGCAACTGTGGGAACACGCCG 3′; 5′ CGGCGTGTTCCCACAGTTGCGGGGG 3′ and 5′ GCCGTCGCGGGTGGGGACCATGA 3′; 5′ TCATGGTCCCCACCCGCGACGGC 3′, respectively. Successful incorporation of the variants was confirmed with Sanger sequencing.

### Immunoblotting.

Immunoblot studies were performed to evaluate the levels of ADAMTSL2 in the intracellular and extracellular matrix. Different cell types were lysed using RIPA buffer (MilliporeSigma, 150 mM NaCl, 1.0% IGEPAL CA-630, 0.5% sodium deoxycholate, 0.1% SDS, 50 mM Tris, pH 8.0). Before SDS-PAGE, cell lysates were resuspended in SDS sample buffer (60 mmol/L Tris-HCl, 1% LDS, 10% glycerol, 0.05% bromophenol blue, pH 6.8, with 2% β-mercaptoethanol). Ten to twenty micrograms of protein per sample was run in a 4%–12% SDS-PAGE from Bio-Rad and transferred to PVDF membranes from Bio-Rad. PVDF membranes were incubated with a blocking solution (TBS containing 0.1% Tween 20 and 5% BSA) and were probed with specific antibodies. Primary antibodies used were ADAMTSL2 (GTX102069) from GeneTex, Fibronectin (F0916) from MilliporeSigma, FBN1 (PA5-99225) from Thermo Fisher Scientific, p-Smad1,5,8 S463/S465 (AB3848) from MilliporeSigma, and Col1A1 (NB600-408) and Col3A1 (NB600-594) from Novus Biologicals. Then we used the DDK (catalog 14793), His (catalog 2365), β-catenin (catalog 8480), Smad1 (catalog 6944), p-Smad2 S465/467 (catalog 3108), Smad2 (catalog 5339), p-Smad3 S423/425 (catalog 9520), Smad3 (catalog 9523), Smad4 (catalog 38454), p-GSK3β S9 (catalog 5558), GSK3β (catalog 12456), p-CaMKII T286 (catalog 12716), CaMKII (catalog 4436), p-AKT S473 (catalog 4060), p-AKT T308 (catalog 13038), AKT (catalog 9272), p-ERK1/2 T202/Y204 (catalog 4377), ERK1/2 (catalog 4695), and GAPDH (catalog 5174) from Cell Signaling Technology. Protein bands were detected using the Chemiluminescence Kit from Thermo Fisher Scientific. ImageJ (NIH) was used to quantify immunoblot results (46). Graphing and 2-tailed *t* test were done in Microsoft Excel. All densitometry analyses were done with 3 independent experiments.

### ELISA.

TGF-β1 quantification from dermal fibroblasts was conducted following previous work in the literature (5, 38). In summary, human dermal fibroblasts (0.5 million cells) (GD001, GD016, and GD017 from donors recruited at the University of Miami and HDFn purchased from Thermo Fisher Scientific) were cultured in 12-well plates for 7 days using DMEM high glucose, pyruvate, 10% FBS, 100 U/mL of penicillin, and 100 μg/mL of streptomycin. Media were changed every 2 days. After confluence, 4 days later, cells were washed twice with PBS. Then the same media without FBS were added because serum had a high amount of bovine TGF-β. Cell lysates (RIPA buffer) and conditioned media were obtained 72 hours later for further analysis. Human and Mouse TGFβ1 DuoSet ELISA from R&D Systems was performed according to manufacturer instructions using conditioned media or mouse tissue lysates.

### Animals.

Mice were housed under a 12-hour light/12-hour dark cycle at 20°C–23°C in specific pathogen–free facilities and supplied with food and water ad libitum.

For the generation of 2 *ADAMTSL2* missense variants (p.R61H and p.A165T), we developed 2 independent knockin mouse models carrying the *ADAMTSL2* missense variants p.R61H and p.A165T by conventional embryonic stem cell–mediated knockin technology using the service of Taconic-Cyagen Model Generation Alliance. Briefly, targeting vectors were constructed with corresponding mutation to target homology arms, electroporated into the embryonic stem cells, and drug selected, and clones were screened by PCR, followed by confirmation with Southern blot and generation of F_1_ by crossing chimeras with WT females. ADAMTSL2-KO mice were obtained from Mutant Mouse Resource and Research Center at University of California, Davis [MMRRC stock 046490-UCD, C57BL/6N-Adamtsl2tm1a(KOMP)Wtsi/MbpMmucd]. Different mouse line crossings were set up to obtain the compound-heterozygous mouse, to model the compound-heterozygous patient and also homozygous, hemizygous, or heterozygous for different combinations of alleles, providing a unique animal model collection useful to dissect genotype-phenotype outcomes.

### Survival and growth curves.

The survival until 6 months was recorded for all mice. A Kaplan-Meier survival curve was done to visualize the survival, and to determine the significant differences the curves were analyzed with the log-rank (Mantel-Cox) test. For growth curves, the weights at specific time points were recorded and plotted. Graphing and 2-tailed *t* test were done in Microsoft Excel. The number of animals is indicated in the figure.

### WBP.

The respiratory function was evaluated in unrestrained conscious mice with Buxco small-animal WBP system (Data Science International) as done in previous publications (47, 48). Mice were trained for 3 consecutive days in the test chamber, and the test was performed on day 4. On test day mice were acclimatized for 10 minutes and then the respiratory parameters measured for 20 minutes. The number of animals tested at week 12 is indicated in the figure; at least 10 animals were measured by week 20 since a few animals died before completion of the test. Graphing and 2-way ANOVA test were done in GraphPad Prism.

### Echocardiography.

Echocardiogram was performed using the Vevo2100 imaging system (VisualSonics) with an MS400 linear array transducer as done in previous work (47, 48). Mice were shaved with depilatory cream 1 day before experiments. Briefly, mice were anesthetized with 2.5%–3% isoflurane at 0.8 L/min flow rate and maintained with 1%–1.5% isoflurane. Following anesthesia, mice were fixed in a supine position on a pad with an integrated temperature sensor, heater, and ECG electrodes. Both heart rate and body temperature were monitored constantly (maintained around 37°C and a heart rate above 450 bpm) during measurement. We used parasternal short (at the level of midpapillary muscles) and long-axis view to obtain 2D B-mode, M-mode, pulse-wave Doppler, and tissue Doppler images. All data were analyzed using VevoLab 3.3.3 software (VisualSonics). The number of animals tested at week 12 is indicated in the figure; at least 10 animals were measured by week 20 since a few animals died before completion of the test. Graphing and 2-way ANOVA test were done in GraphPad Prism.

### CT imaging.

All mice were CT imaged on the MILabs Vector 6 PET/SPECT/CT using the following imaging parameters: whole-body scan, accurate scan mode, step angle: 0.500, projections per step: 1, binning: 1 × 1, tube voltage: 50 kV, tube current: 0.21 mA, exposure time: 75 ms, imaging time: 00:02:41 (hh:mm:ss), dose estimate: 69 mGy. Images were subsequently reconstructed using the MILabs reconstruction software. All image analysis was generated using Imalytics Preclinical 2.1 software. All bone measurements were performed with images with a threshold of 7,500 HU and representative 3D images with a threshold of 2,700 HU.

Femur measurements were done along the longest axis, from the greater trochanter to the trochlea. The width of the femur was done using the third trochanter as the reference point (Supplemental Figure 4S1). Proximal phalange lengths from the second, third, and fourth digits were measured. The width of each phalange was measured near the central part of the phalange at the thinnest section of the bone (Supplemental Figure 4S1). The nasal bone was measured from the nasofrontal suture at the median sagittal plane to the frontal tip of the bone (Supplemental Figure 4S1). The number of animals is indicated in the figure. Graphing and 1-way ANOVA test were done in GraphPad Prism.

### MRI imaging.

For the heart, a CINE bright blood intra-gate flow compensated scan was done, with a 2.9 ms echo time, 48 ms repetition time, 35° flip angle, 200 oversampling, 14 movie frames, 25 × 25 mm field of view with a 192 × 192 matrix size, a 130 μm^2^ in-pane resolution, 6 slices, 1 mm thickness. In the CINE movies of the heart, the cardiac cycle is divided into 14 frames. End diastole and end systole frames were selected, and the endocardial borders of the left ventricle were manually defined in the short-axis view, in each of the 6 slices, excluding the papillary muscles. For each animal, the total EDV and ESV were calculated using the ‘-V’ option of the command line tool *fslstats* of FSL (FMRIB Software Library v6.0), a comprehensive library of analysis tools for MRI data.

The EF was calculated as EF (%) = ([EDV – ESV]/EDV) × 100.

For the lungs, ultra-short echo time 3D scan of the lungs in the expiration phase was performed, with 0.007 ms echo time, 4 ms repetition time, 94,872 projections, 5° flip angle, 250 scans of 1,000 ms, a field of view of 27 × 27 × 27, a matrix size of 174 × 174 × 174, a resolution of 155 μm^3^, respiratory gated. The number of animals is indicated in the figure. Graphing and 2-tailed *t* tests were done in GraphPad Prism.

### Histology.

After 7 months, the heart and lungs were collected from female mice. For the collection mice were anesthetized, the heart was harvested by cutting great vessels and immediately immersed in ice-cold PBS to allow a few beats to pump out all the blood, and then the heart was transferred to ice-cold 1 M potassium chloride solution to stop it in diastole. We then placed it in 4% paraformaldehyde (PFA). The lungs were inflated with a cannula into the trachea with 4% PFA with a flow rate no greater than 200 μL/s. The inflated lung was held for a few seconds and then placed in 4% PFA. The lungs and the hearts were embedded in paraffin. Routine H&E, Movat, Picrosirius Red, and Masson’s trichrome stains were performed on paraffin tissue sections. Immunohistochemistry was performed with lung sections with CD68 macrophage marker. An independent pathologist reviewed sections of the lungs and trachea. The number of animals is indicated in the figure.

β-Galactosidase staining was performed in newborn mice using frozen sections. In summary, fresh-frozen sections were fixed with 4% PFA for 10 minutes. Slides were washed 3 times with LacZ wash solution (2 mM MgCl_2_, 0.01% NaDeoxycholate, 0.02% Nonidet P-40, in PBS), then incubated at 37°C with X-gal MilliporeSigma [0.5 mg/mL X-Gal, 5 mM K_3_Fe(CN)_6_, 5 mM K_4_Fe(CN)_6_ in LacZ wash solution] and counterstained with nuclear fast red. Sections were dehydrated with serial wash of ethanol and xylenes and mounted with Eukitt medium from Electron Microscopy Sciences.

### Tissue extraction.

Hearts and lungs of newborns and 8-month-old adult female mice were collected. Animals were perfused with PBS and tissue was immediately collected and snap-frozen in liquid nitrogen. Tissue was stored at –80°C. Samples were pulverized in dry ice with mortar and pestle. For Adamtsl2 immunoblots in adult lung tissue, we followed the extraction procedure from Alexandra Naba Lab (49, 50). We processed 120 mg of material. Tissue homogenization was performed as instructed with Omni homogenizer and with a subcellular protein fractionation kit for tissues from Thermo Fisher Scientific. Homogenized aliquots were diluted with 400 μL buffer extraction solution (8% SDS, 200 mM Tris-HCl pH 7.4 EDTA 2 mM) and used for immunoblots. For other proteins, extraction of 100 μg of tissue was done with RIPA buffer from MilliporeSigma supplemented with Pierce protease inhibitors from Thermo Fisher Scientific and phosphatase inhibitors cocktail 2 and 3 from MilliporeSigma. All samples were sonicated and homogenized with a UP200St ultrasonic processor from Hielscher Ultrasonics. Samples were centrifuged 5,000*g* for 5 minutes at 4°C, and the supernatant was used for protein analysis.

### Statistics.

Graphing, 2-tailed *t* test, 1-way ANOVA, 2-way ANOVA, and log-rank test were done in Microsoft Excel and in GraphPad Prism. The number of animals used is indicated in figures. *P* < 0.05 was considered statistically significant.

### Study approval.

The present studies in animals and humans were reviewed and approved by the University of Miami Institutional Animal Care and Use Committee, according to the National Institutes of Health guidelines, and by the University of Miami Institutional Review Board. Patient GD001 was evaluated by our team of clinical geneticists at the University of Miami. Short- and long-read genome sequencing along with Sanger sequencing were conducted to confirm the diagnosis. The patient’s family gave written informed consent for the testing and sharing of the research data. For the use of primary fibroblasts, written informed consent was obtained from the patient’s family and adult participants.

### Data availability.

Raw data associated with this study are reported in the Supporting Data Values file and unedited blot images file. Any additional information required to analyze the data reported in this paper is available from the corresponding author upon request.

## Author contributions

VC conducted experiments, acquired data, analyzed data, and wrote the manuscript. MMW conducted experiments, acquired data, and analyzed data. AAM conducted experiments, acquired data, analyzed data, and reviewed the manuscript. MFZ conducted experiments. OVK analyzed data. AK acquired data. CA acquired data. MAR analyzed data. LMB analyzed data. LP acquired reagents. GB acquired data and analyzed data. DSB analyzed data. SS analyzed data. GW analyzed data and reviewed the manuscript. LAS analyzed data. KW analyzed data and reviewed the manuscript. MT designed the research study, analyzed data, and reviewed the manuscript.

## Supplementary Material

Supplemental data

Unedited blot and gel images

Supplemental video 1

Supporting data values

## Version 1 02/01/2024

In-Press Preview

## Version 2 03/08/2024

Electronic publication
