# Supplementary material for: ADAMTSL2 mutations determine the phenotypic severity in geleophysic dysplasia
Source: JCI Insight. 2024 Feb 1;9(5):e174417. doi: 10.1172/jci.insight.174417 (PMC10972594; doi:10.1172/jci.insight.174417)
Supplement: Supplemental data [file jciinsight-9-174417-s030.pdf]

Table S1. Clinical diagnosis and mutations identified in patient GD001 and general characteristic of all the primary cells used in the study.

	GD001	HDFn	GD016	GD017
**Diagnosis**	GD1	Control	Control	Control
**Height**	86.4cm<br>(z-score -1.16)	N/A	N/A	N/A
**Cardiac pathology**	PS, AS, PDA, VSD	N/A	N/A	N/A
**Respiratory involvement**	Frequent infections	N/A	N/A	N/A
**Mutation**	ADAMTSL2 c.182G>A, p.R61H,<br>ADAMTSL2 c.493G>A, p.A165T<br>compound heterozygous	N/A	N/A	N/A
**Sex**	male	male	male	male
**Age at tissue collection**	23 months	neonate	3 weeks	7 months
**Isolation procedure**	Explant	Enzymatic digestion	Explant	Explant

Table S1. Clinical diagnosis and mutations identified in patient GD001. This patient was modeled for an individual and personalized medical research approach of Geleophysic Dysplasia type 1 (GD1). General characteristics of control cells used in the study. The height measurement provided was at birth.

Figure 1S1

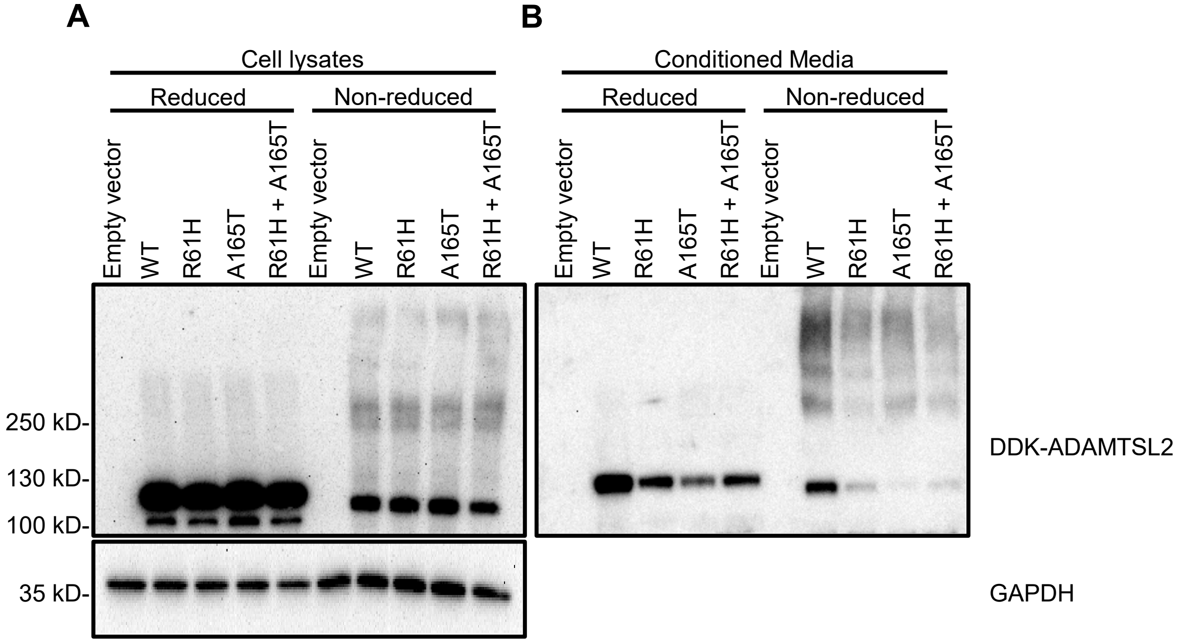

Figure 1S1. The p.R61H or p.A165T mutation does not cause aberrant dimerization of ADAMTSL2. (A) HEK293T cells were transfected with either Empty vector, wild type (WT), p.R61H, p.A165T, or p.R61H + p.A165T ADAMTSL2-DDK. Cell lysates and conditioned media (CM) were collected at 24 and 72 hours respectively. DDK-ADAMTSL2 was determined in reducing or non reducing conditions to visualized the presence of abnormal dimer formation. In each of the conditions tested, the mutants and the WT had the same pattern of migration suggesting that there are not aberrant dimers with p.R61H or p.A165T mutation.

Figure 1S2

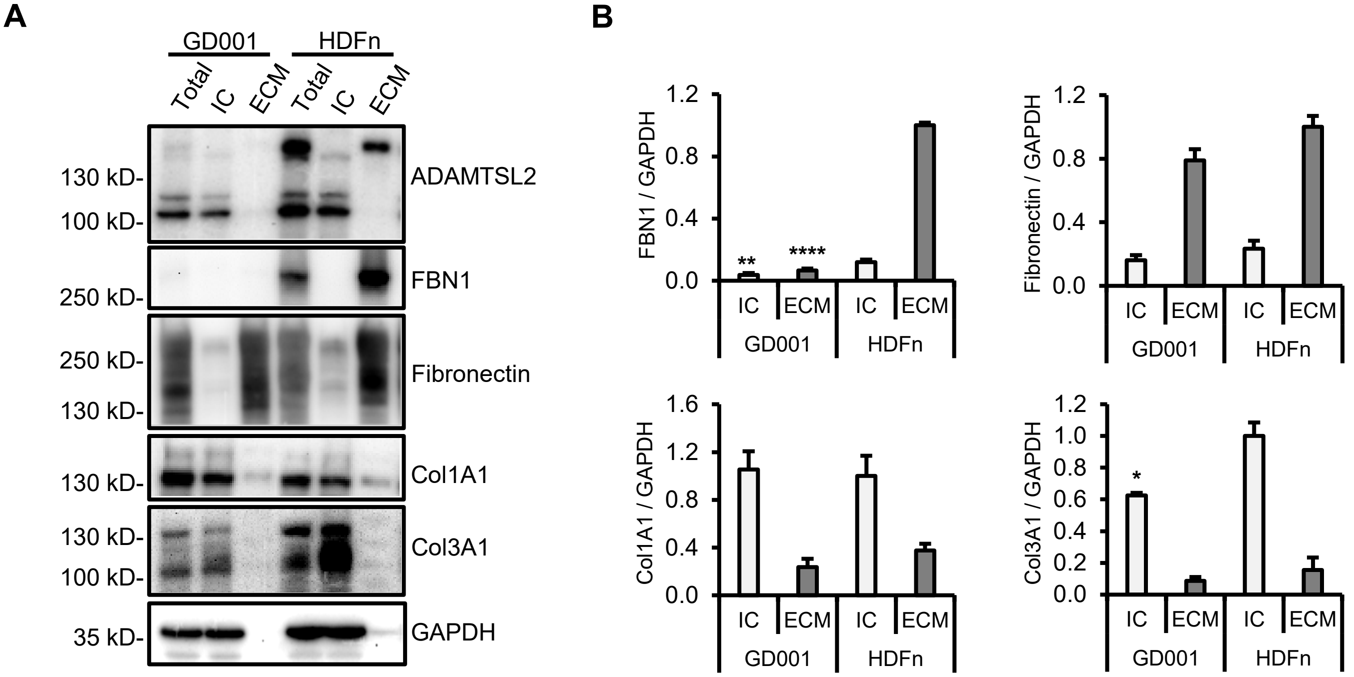

Figure 1S2. Mutant ADAMTSL2 cells have a reduced expression of tested ECM proteins. (A) ADAMTSL2, FBN1 and Collagen type III are reduced in ADAMTSL2-related GD fibroblasts. (B) Densitometry analysis of the FBN1/GAPDH ratio shows a significant reduction of FBN1 in the extracellular and intracellular space and Collagen type III in the intracellular space of GD001 human dermal fibroblasts when compared with human dermal control fibroblasts. (*, p<0.05; **, p<0.01; ****, p<0.0001). Densitometry analysis was done with independent experiments (5 for ADAMTSL2, 4 for FBN1, 6 for Fibronectin, and 2 for Col1A1 and Col1A3).

Figure 1S3

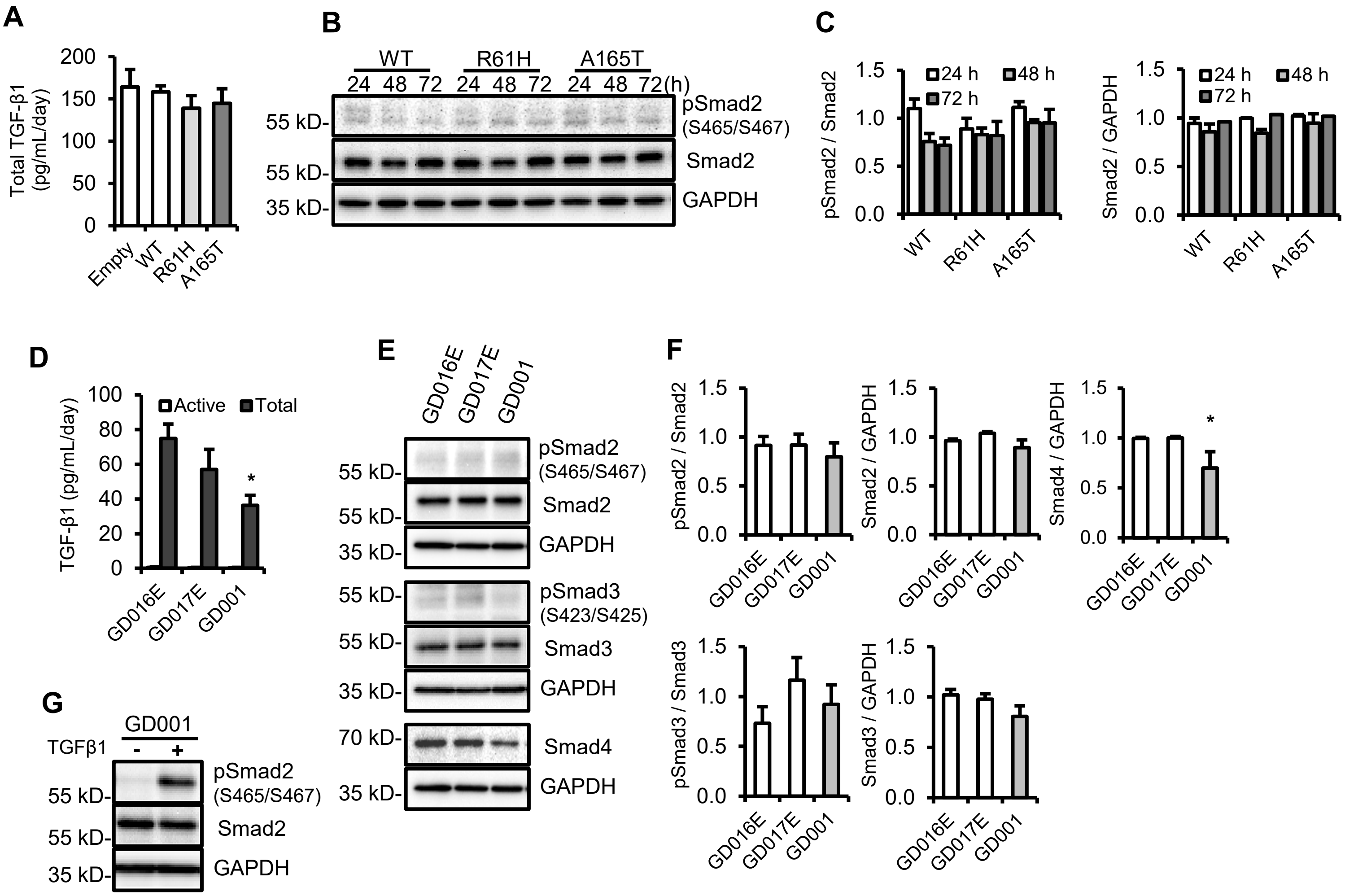

Figure 1S3. TGFβ signaling is not significantly increased in cells with p.R61H or p.A165T ADAMTSL2. (A) ELISA quantification of TGFβ1 (pg/mL/day) in HEK293T conditioned media collected at 72h post transfection with either WT, p.R61H or p.A165T ADAMTLS2. (B) Immunoblot for pSmad2 (S465/S467), Smad2, GAPDH in HEK293T at 24, 48 and 72h post transfection with either WT, p.R61H or p.A165T ADAMTLS2. (C) Densitometry analysis of pSmad2/Smad2 and Smad2/GAPDH ratios of immunoblot in (B). (D) ELISA quantification of TGFβ1 (pg/mL/day) produced in proband primary fibroblasts (GD001) and control primary fibroblast (GD016E and GD017E). (E) Immunoblot for pSmad2 (S465/S467), Smad2, pSmad3 (S423/S425), Smad3, Smad4 and GAPDH of primary fibroblast. (F) Densitometry analysis of pSmad2/Smad2, pSmad3/Smad3, Smad2/GAPDH, Smad3/GAPDH, and Smad4/GAPDH ratios of immunoblot in (E). (G) Immunoblot for pSmad2 (S465/S467), Smad2, and GAPDH 24 hours after TGFβ1 treatment in primary fibroblast showsthat the TGFβ pathway is not impaired. (*, p<0.05). All densitometry analysis were done with 3 independent experiments.

Figure 1S4

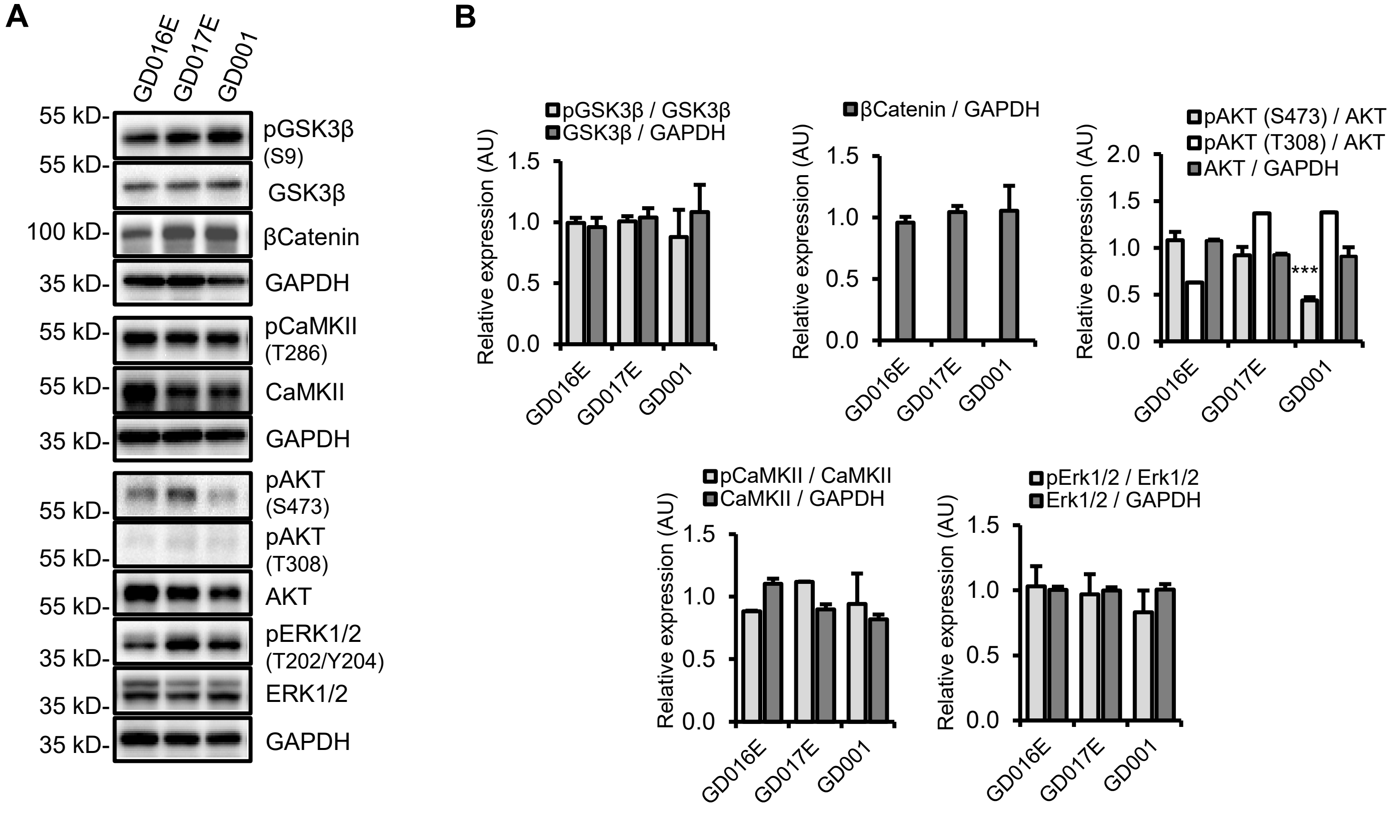

Figure 1S4. Pathway analysis in proband primary fibroblast. (A) Immunoblot for pGS3β (S9), GS3β, βCatenin, pCaMKII (T286), CaMKII, pAKT (S473), pAKT (T308), AKT, pERK1/2 (T202/Y204), ERK1/2 and GAPDH in proband`s primary fibroblasts (GD001) and control primary fibroblasts (GD016E and GD017E). (F) Densitometry analysis of pGSK3β/GSK3, GSK3β/GAPDH, βCatenin/GAPDH, pAKT (S473)/AKT, pAKT (T308)/AKT, AKT/GAPDH, pCaMKII/CaMKII, CaMKII/GAPDH, pERK1/2 (T202/Y204)/ERK1/2 and ERK1/2/GAPDH (*, p<0.05; **, p<0.01; ***, p<0.001) . All densitometry analyses were done with 3 independent experiments.

Figure 2S1

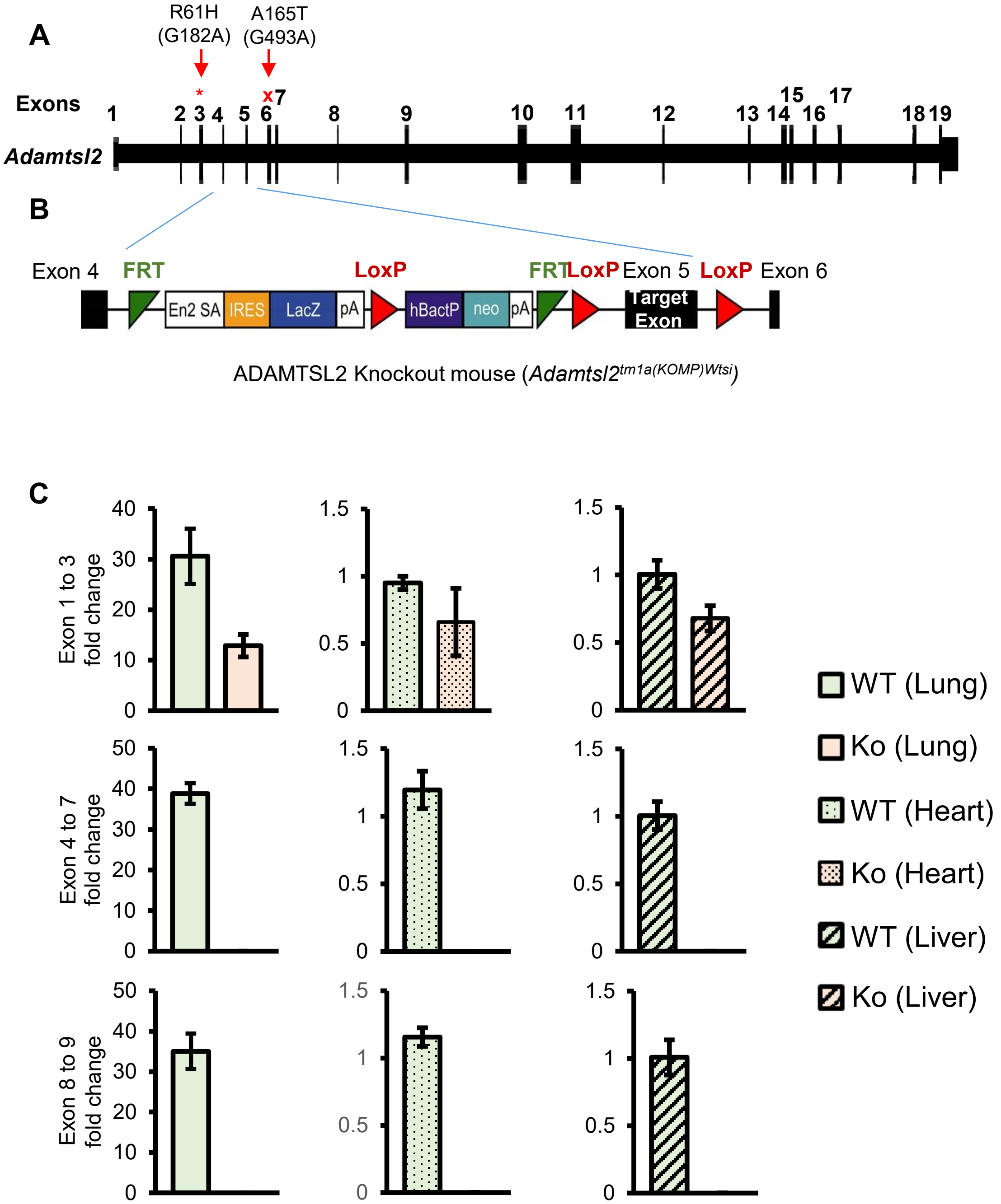

Figure 2S1. Generation of personalized mouse model. Based on patient GD001`s mutations, two independent animal models were generated with conventional embryonic stem cell- mediated knock-in technology. (A) Graphical representation showing the location of each knock-in (KI). *Adamtsl2* c.182G>A, p.R61H (in exon 3) and Adamtsl2 c.493G>A, p.A165T in exon 6 (G493A). (B) Targeted deletion of *Adamtsl2* was obtained by the purchase of *Adamtsl2*tm1c(KOMP)Wtsi mice with a gene trapping cassette inserted downstream of exon 4. (C) Real time PCR from lungs, livers, and hearts of newborn littermate mice showing the disruption of mRNA expression after the gene trapping cassette in FRTLoxPKo/FRTLoxPKo animals. Relative values normalized to liver WT samples. Compound heterozygous, homozygous, hemizygous, and heterozygous mice were created by generating and crossing p.R61H Knock-in (KI) *Adamtsl2*, p.A165T Knock-in (KI) *Adamtsl2*, Knock-out (Ko) *Adamtsl2*, and WT mice.

Figure 2S2

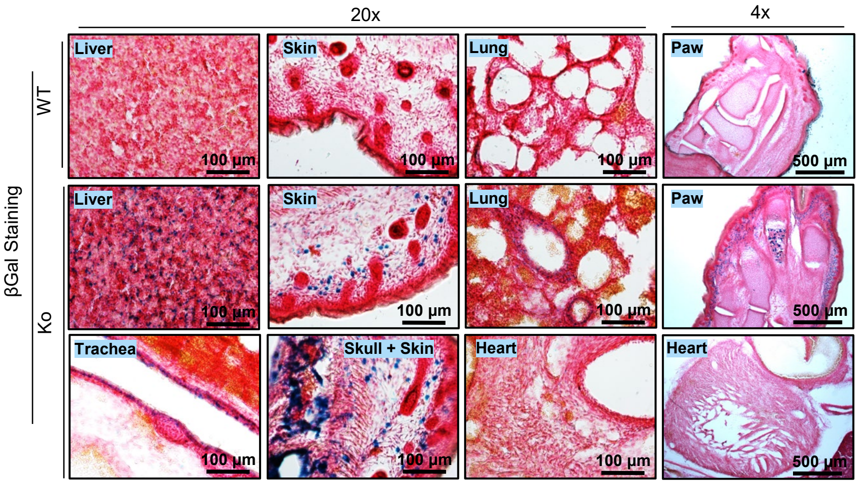

Figure 2S2. βGalactosidase staining of newborn ADAMTSL2 Knockout mouse (*Adamtsl2*$^{tm1a(KOMP)Wtsi}$) compared with WT littermate. The staining shows LacZ expression (blue staining) under the ADAMTSL2 promoter in the liver, skin, lungs, paws, skull, heart, and trachea. Counterstaining was done with Nuclear Fast Red. Histology was done with serial sections from 1 WT and 1 FRTLoxPKo/FRTLoxPKo.

Figure 2S3

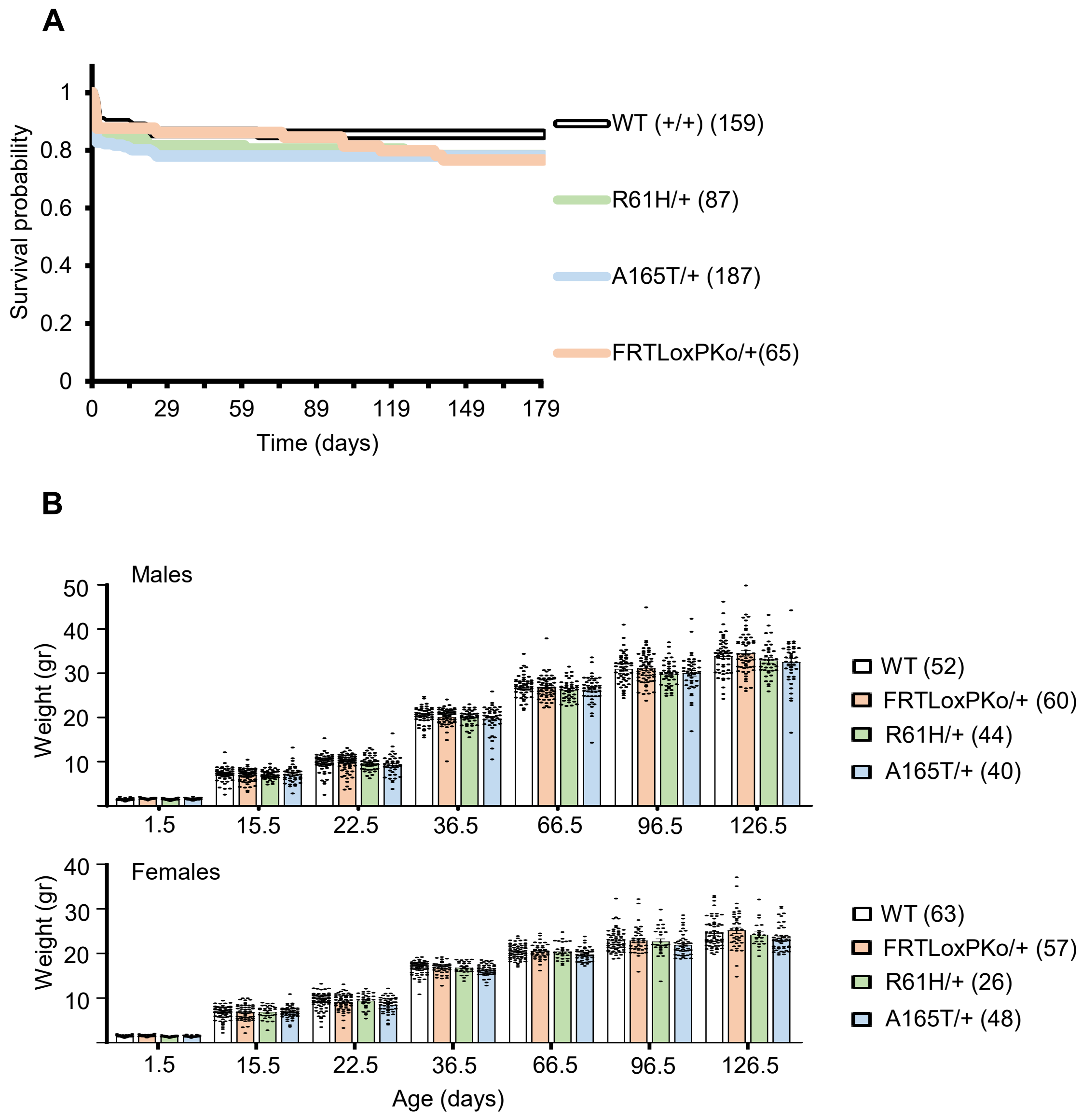

Figure 2S3. Survival and growth of mutant mice associated with *Adamtsl2* allelic combinations. One WT allele is sufficient to prevent any significant reduction in growth or survival during the first months life of the animals. (A) Kaplan-Meier survival curve of mice with different allelic combinations in *Adamtsl2* during the first 6 months. The curves were compared with log-rank (Mantel-Cox) test to determine significance. (N= number of animals). (B) Growth curve of male (top) and female (bottom). Average +/- SEM. (*, $p<0.05$) (N= number of animals).

Figure 3S1

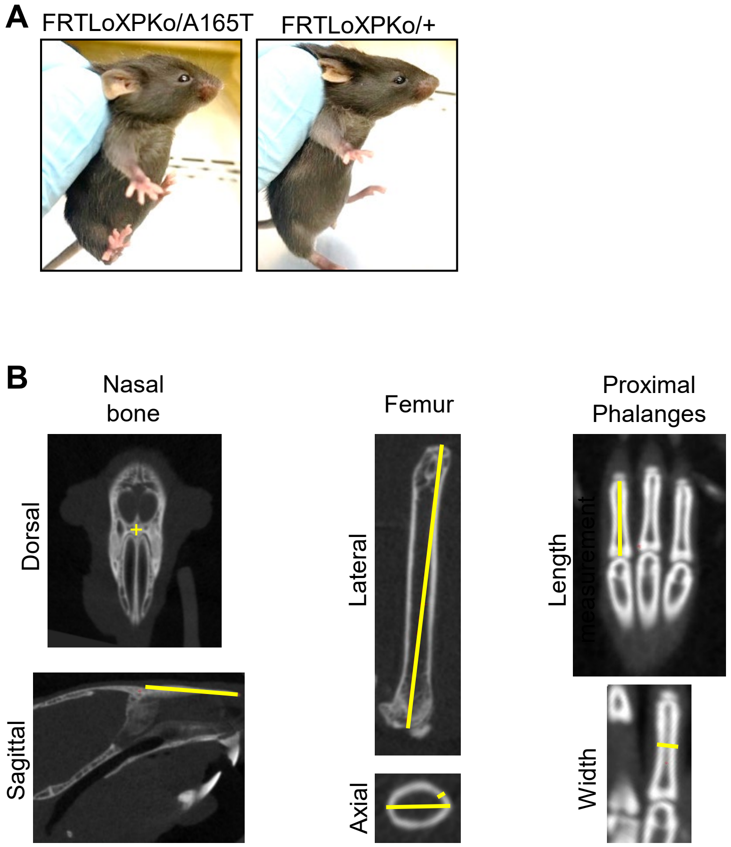

Figure 3S1. Different degrees of skeletal abnormalities were found in *Adamtsl2* mutant mice. (A) Head morphology in FRTLoXPKo/A165T with a reduced snout when compare with FRTLoXPKo/+ littermates. (B) Examples of actual CT scan images and anatomical points used for measurements of the nasal bone, femur, and phalanges. compared

Figure 4S1

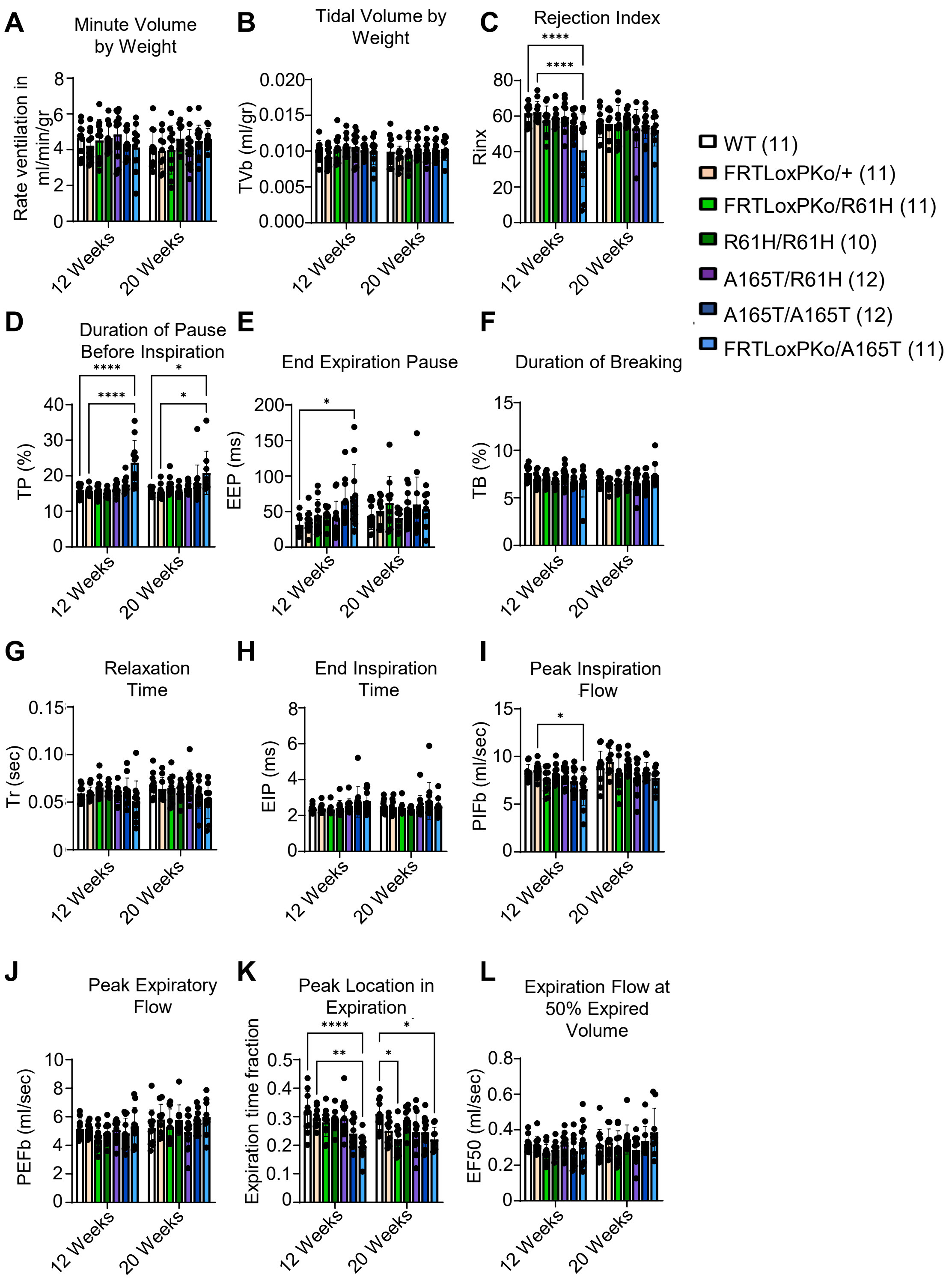

Figure 4S1. Respiratory functions are altered in *Adamtsl2* hemizygous and homozygous p.A165T mice. (A-L) Respiration was analyzed with whole body plethysmography at 12 and 20 weeks of age in male and female mice. Indicators of expiration are significantly affected in mice with p.A165T mutation. (N= number of animals) (*, p<0.05, **, p<0.01, ***, p<0.001, ****, p<0.0001). (TVb = Tidal Volume by Weight, Rinx = Rejection Index, TP = Duration of Pause before insipiration, EEP = End Expiration Pause, TB = Duration of Breaking, Tr = Relaxation Time, EIP = End Inspiration Time, PIFb = Peak Inspiration Flow, PEFb = Peak Expiration Flow, EF50 = Expiration Flow at 50% Expired Volume).

Figure 4S2

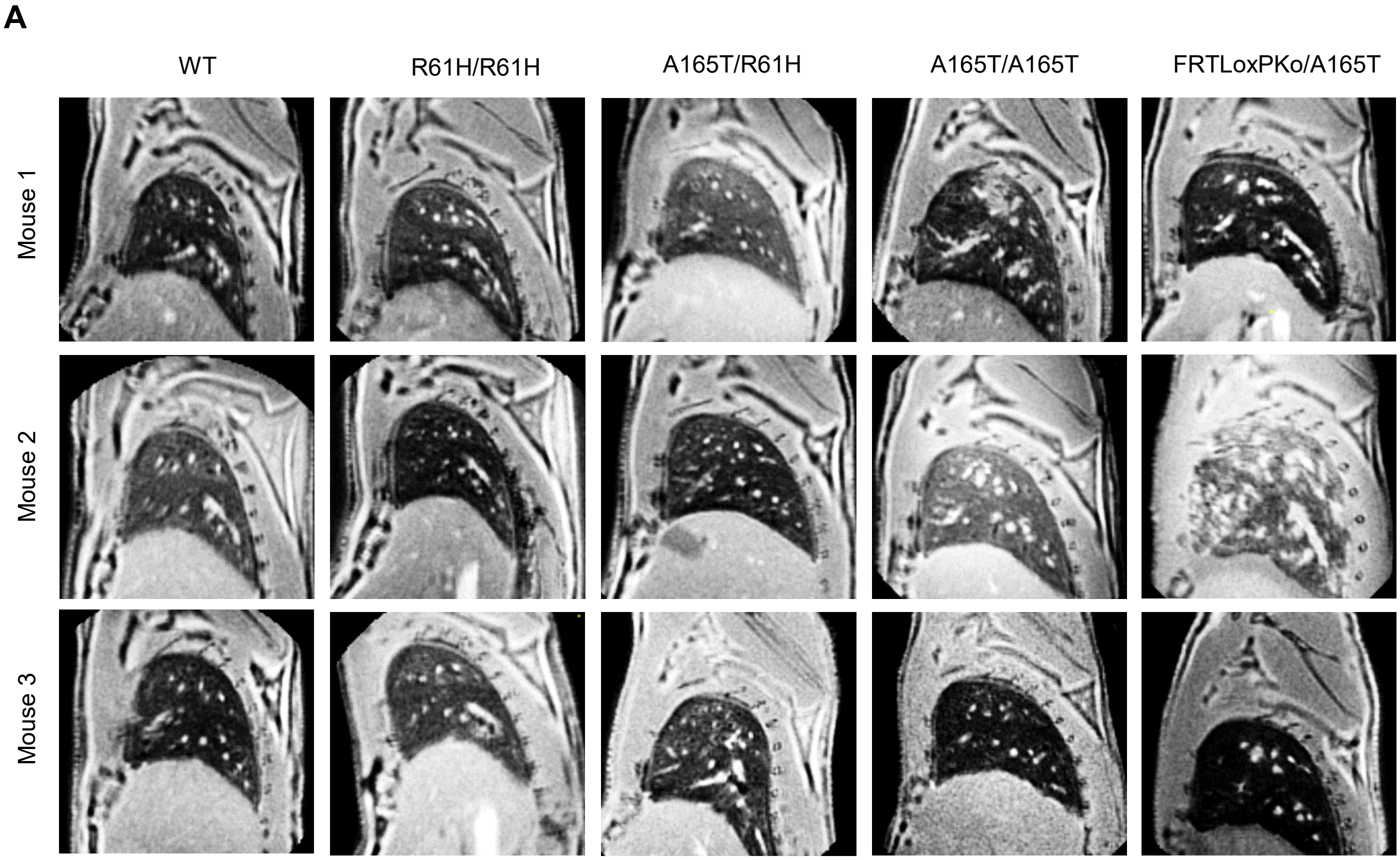

Figure 4S2. MRI images show abnormalities in the lungs of some *Adamtsl2* hemizygous and homozygous p.A165T mice. (A) MRI scan shows ground glass opacifications and/or consolidations in the lungs of some of the mutant animals. MRI was performed in 4 WT, 4 R61H/R61H, 4 R61H/A165T, 5 A165T/A165T, and 3 FRTLoXPKo/A165T in 6 month of age male mice. Images showing the right lung of 3 animals per group. WT mouse 3, A165T/A165T mouse 1, and FRTLoxPKo/A165T mouse 2 images are shown in Figure 4G.

Figure 5S1

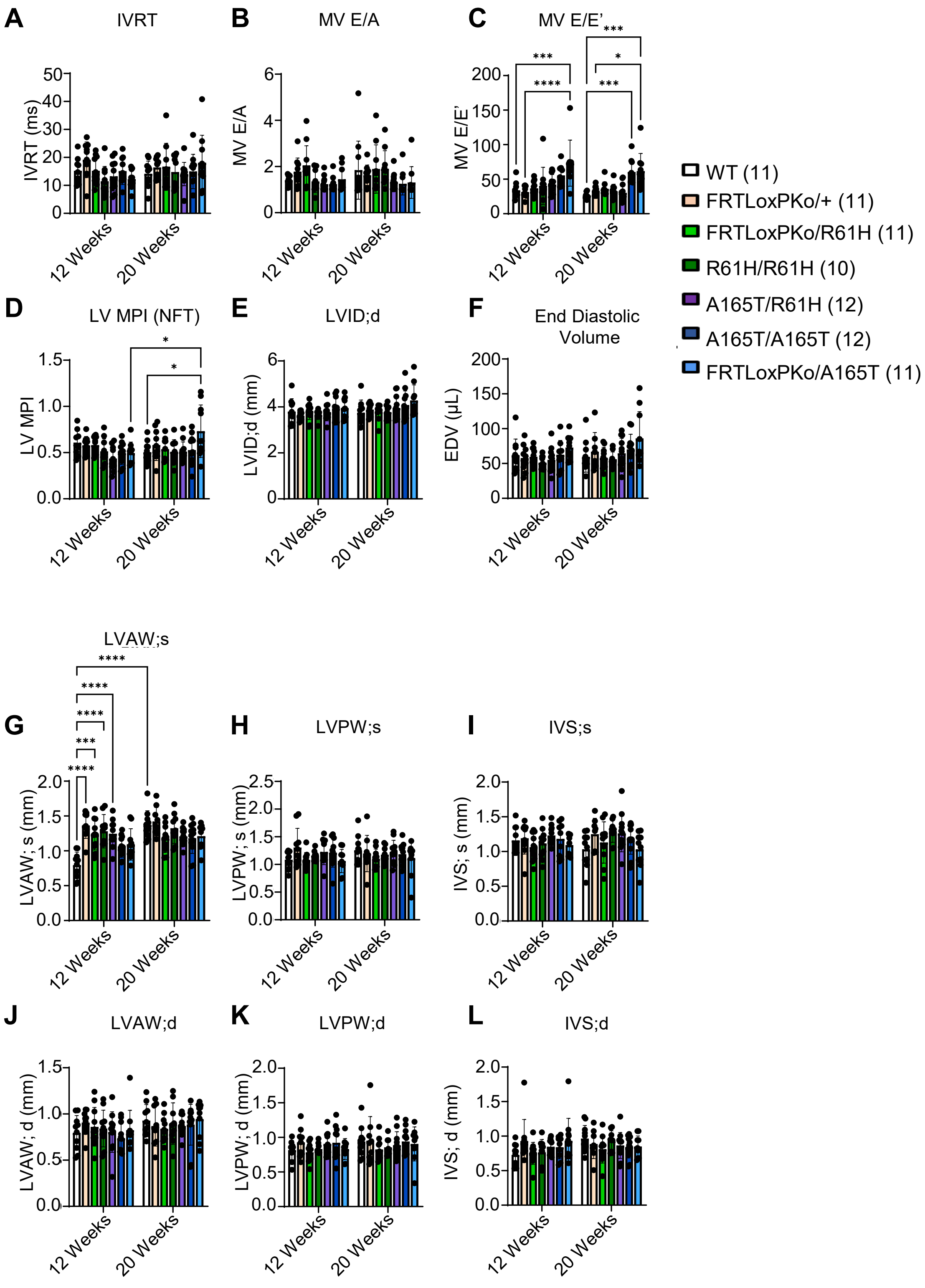

Figure 5S1. Heart function and structure are altered in *Adamtsl2* hemizygous and homozygous p.A165T mice. (A-F) Cardiac diastolic function was analyzed with echocardiogram at 12 and 20 weeks of age in male and female mice. (G-L) Heart wall thickness is altered in mice with *Adamtsl2* mutation. (N= number of animals) (*, *p*<0.05; **, *p*<0.01; ***, *p*<0.001; ****, *p*<0.0001). (IVRT = Isovolumic relaxation time, MV E/A = mitral valve E-wave/A-wave ratio), MV E/E'= mitral valve E-wave/peak e'velocity ratio), LV MPI = Left ventricle myocardial performance index, LVID;d = left ventricular internal diameter end diastole, EDV = End Diastolic Volume, LVAW;s = left ventricular end-systolic anterior wall thickness, LVPW;s = left ventricular end-systolic posterior wall thickness, LVAW;d = left ventricular end-diastolic anterior wall thickness, LVPW;d = left ventricular end-diastolic posterior wall thickness, IVS;s = interventricular end-systolic septal thickness , IVS;d = interventricular end-diastolicc septal thickness).

Figure 5S2

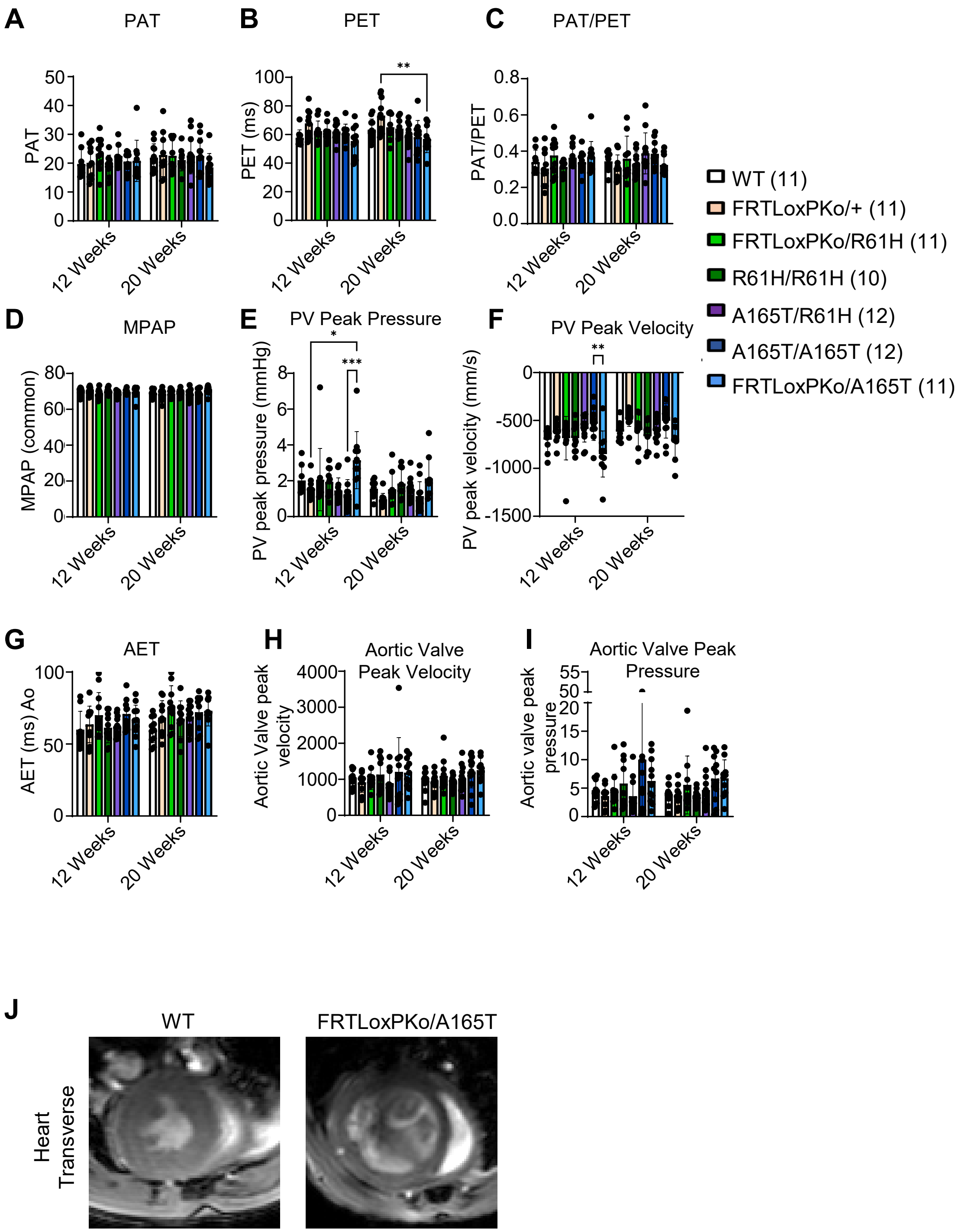

Figure 5S2. Heart functions are altered in *Adamtsl2* hemizygous and homozygous p.A165T mice. (A-I) Heart-pulmonary and heart-aortic functions were analyzed with echocardiogram at 12 and 20 weeks of age in male and female mice. (N= number of animals) (*, $p<0.05$; **, $p<0.01$; ***, $p<0.001$; ****, $p<0.0001$). (J) Dynamic of tMRI scan showed a deficit in the heart systolic function. MRI scan done in 4 WT, 4 R61H/R61H, 4 A165T/R61H, 3 A165T/A65T, and 2 FRTLoXPKo/A165T. MRI done in 6 month of age male mice. (PAT = pulmonary acceleration time, PET = Pulmonary ejection time, PAT/PET= pulmonary acceleration time and ejection time ratio, MPAP = mean pulmonary arterial pressure, PV = pulmonary valve, AET = Aortic Ejection Time).

Figure 5S3

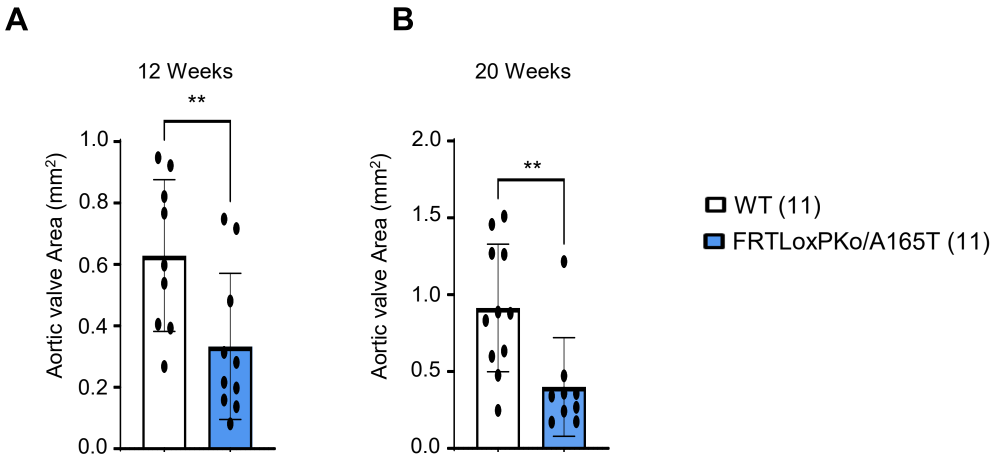

Figure 5S3. Aortic root/valve narrowing was found in *Adamtsl2* hemizygous p.A165T mice. The aortic root/valve narrowing was quantified by determining the aortic valve area (AVA). (A-B) Aortic valve area quantified with echocardiogram at 12 and 20 weeks of age in male and female mice. (N=number of animals) (**, p<0.01).

Figure 6S1

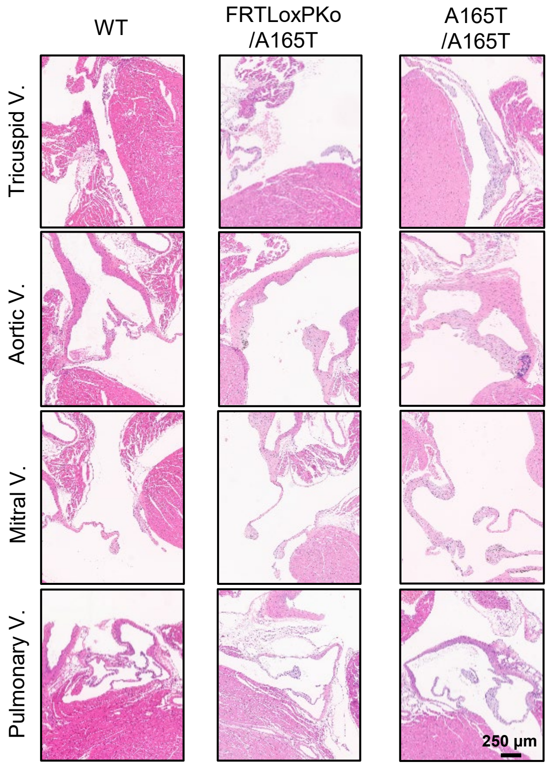

Figure 6S1. Representative heart valve sections of 7 months old male mice with *Adamtsl2* p.A165T mutation. Thickness and length of the valves were not measured due to variability in the 3D orientation of the valves during the sectioning. Histology done with serial section of the heart from 3 WT, 3 A165T/A65T, and 3 FRTLoXPKo/A165T.

Figure 6S2

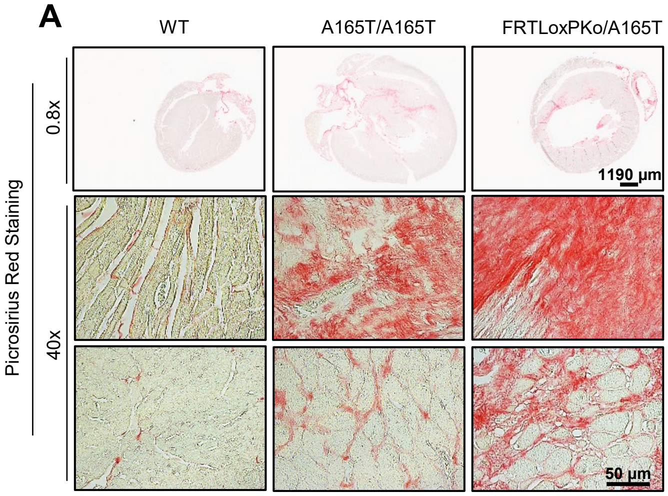

Figure 6S2. Picrosirius red staining confirms that in *Adamtsl2* hemizygous and homozygous p.A165T mice with a severe phenotype, the muscle tissue in the ventricles has areas of mild interstitial fibrosis. Areas with red staining indicate interstitial fibrosis due to the high collagen content. All the animals were 7 months of age male mice. Histology was done with serial heart sections from 2 WT, 2 A165T/A65T, and 3 FRTLoXPKo/A165T.

Figure 7S1

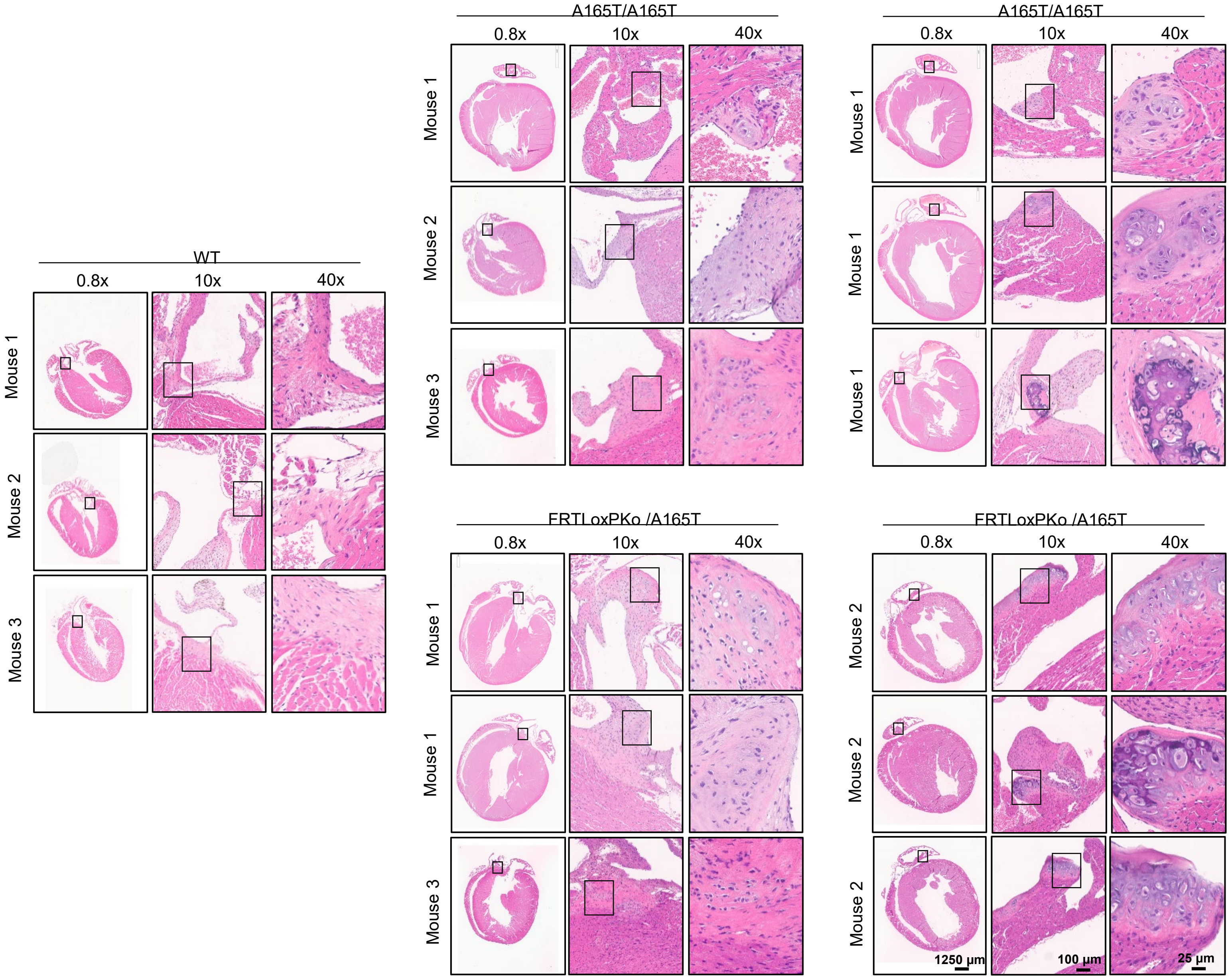

Figure 7S1. Heart histology reveals hypertrophic cardiomyopathy, myocyte hypertrophy, and small areas with chondroid tissue in mice with *Adamtsl2* p.A165T mutation. Serial histology staining of the whole heart of WT, A165T/A165T, and FRTLoxPKo/A165T mice. (A) There is chondroid tissue in some small areas of the atrium and aortic valve of a FRTLoxPKo/A165T and an A165T/A165T mice. The remaining animals had areas with mild chondroid-like cells near the valves or in the atrium while the 3 WT littermates did not show any abnormalities. Histology was done with serial heart sections from 3 WT, 3 A165T/A65T, and 3 FRTLoXPKo/A165T. Histology done in 7-month-old male mice. A subset of these images from WT, A165T/A165T, and FRTLoxPKo/A165T mice that better show the chondroid tissue are also presented in Figure 7.

Table S2. Summary of the main findings supporting a cardiac dysfunction in A165T variant.

Echocardiogram	MRI	Histology
Progressive systolic dysfunction	Systolic dysfunction	Hypertrophic Cardiomyopathy
↓ Ejection Fraction	↓ Ejection Fraction	Myocyte hypertrophy
↓ Stroke Volume	↑ end-systolic volume	Patchy mild interstitial fibrosis
↓ Cardiac Output		chondroid metaplasia in small areas of the aortic valve and atrium
↓ Fractional shortening		
↑ left ventricular end-systolic internal diameter		
↑ end-systolic volume		
↑ ventricular filling pressure		
↑ Left ventricular end-systolic anterior wall thickness		
↓ Pulmonary ejection time		
↑ Pulmonary valve peak pressure		
↓ aortic valve area		
Cardiac insuficiency		

Table S2. Summary of the main findings supporting a cardiac dysfunction in A165T variant.

Figure 8S1

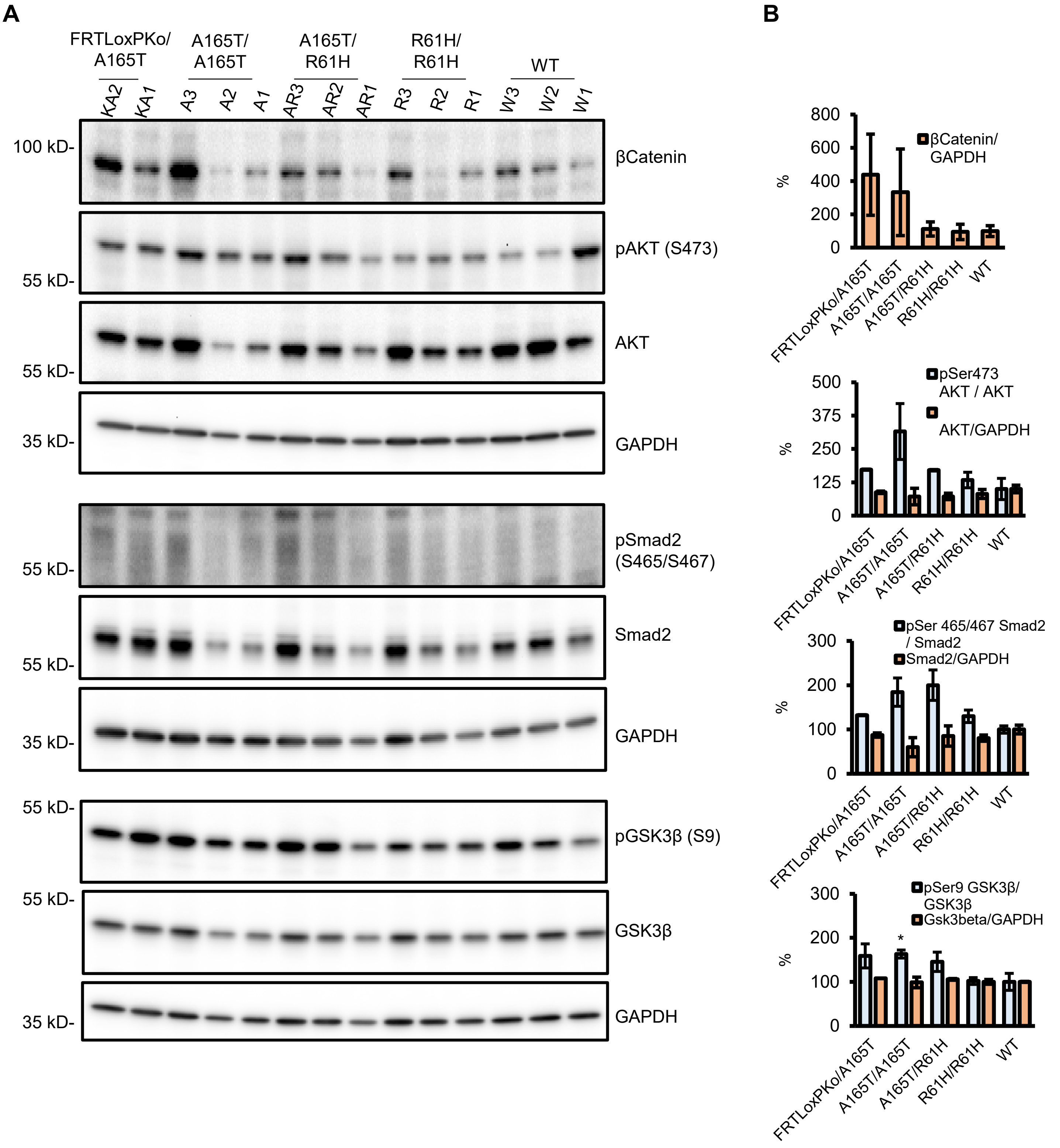

Figure 8S1. Pathway analysis of mutant *Adamtsl2* 8-months-old female mouse hearts. (A) Immunoblot for βCatenin, pAKT (S473). AKT, pSmad2 (S465/S467), Smad2, pGS3β (S9), GS3β, and GAPDH in hemizygous and homozygous p.A165T mice, homozygous p.R61H, compound heterozygous p.A165T/p.R61H and WT mice. (B) Densitometry analysis of βCatenin/GAPDH, pAKT/AKT, AKT/GAPDH, pSmad2/Smad2, Smad2/GAPDH, pGSK3β/GSK3 and GSK3β/GAPDH. (*, $p<0.05$).

Figure 8S2

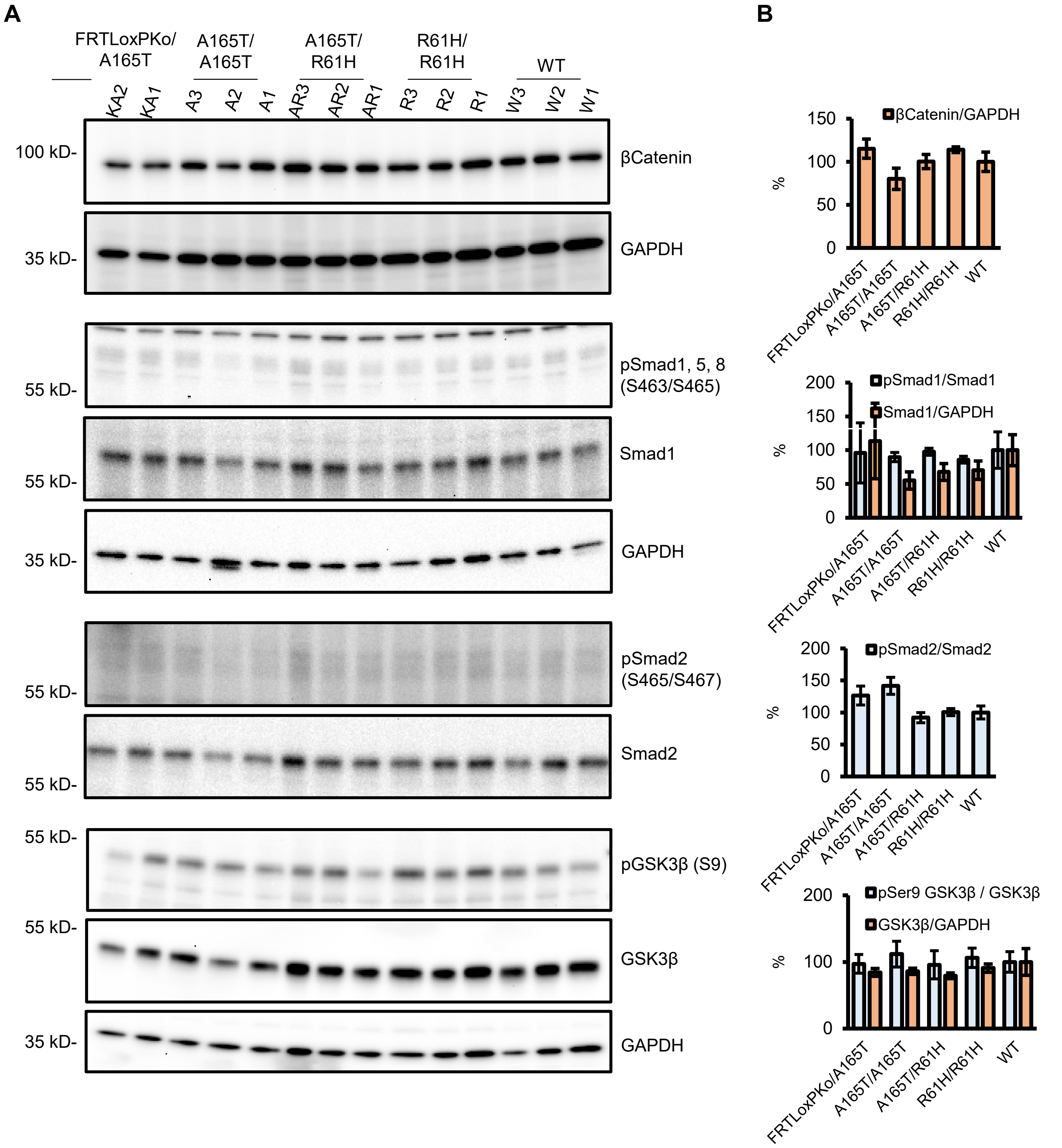

Figure 8S2. Pathway analysis of mutant *Adamtsl2* 8-months-old female mouse lungs. (A) Immunoblot for βCatenin, pSmad1, 5, 8 (S463/S465), Smad1 , pSmad2 (S465/S467), Smad2, pGS3β (S9), GS3β, and GAPDH in hemizygous and homozygous p.A165T mice, homozygous p.R61H, compound heterozygous p.A165T/p.R61H and WT mice. (B) Densitometry analysis of βCatenin/GAPDH, pSmad1,5,8/Smad1, Smad1/GAPDH, pSmad2/Smad2, pGSK3β/GSK3 and GSK3β/GAPDH.
