# Supplementary material for: ADAMTSL2 mutations determine the phenotypic severity in geleophysic dysplasia
Source: JCI Insight. 2024 Feb 1;9(5):e174417. doi: 10.1172/jci.insight.174417 (PMC10972594; doi:10.1172/jci.insight.174417)

1A

DDK-cells

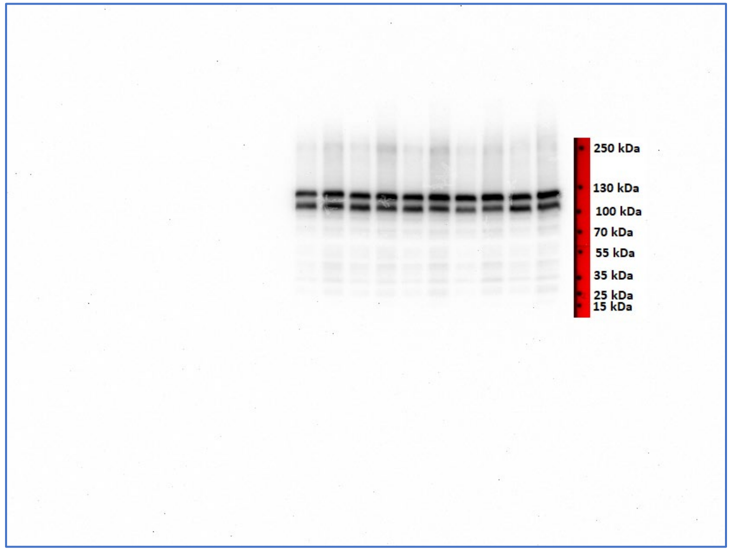

GAPDH-cells

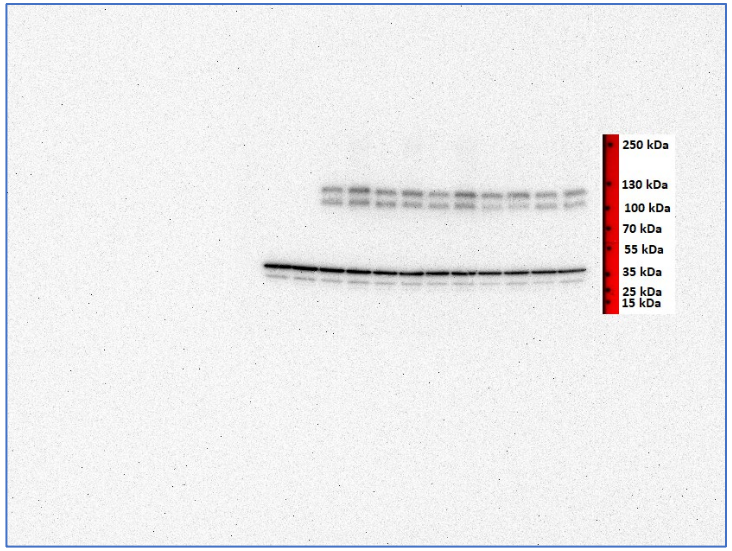

DDK-CM

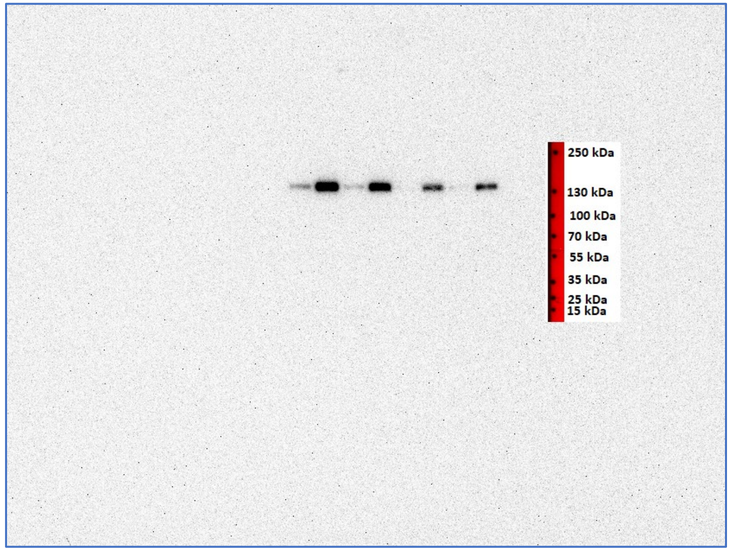

1C

DDK-cells

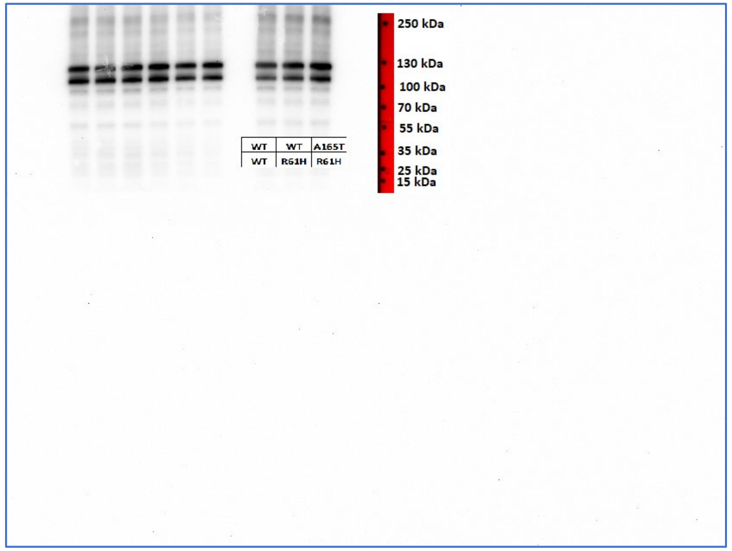

GAPDH-cells

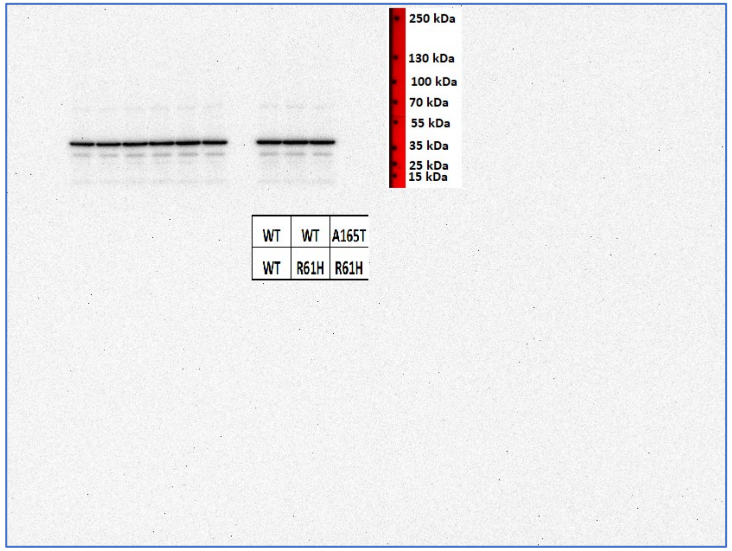

DDK-CM

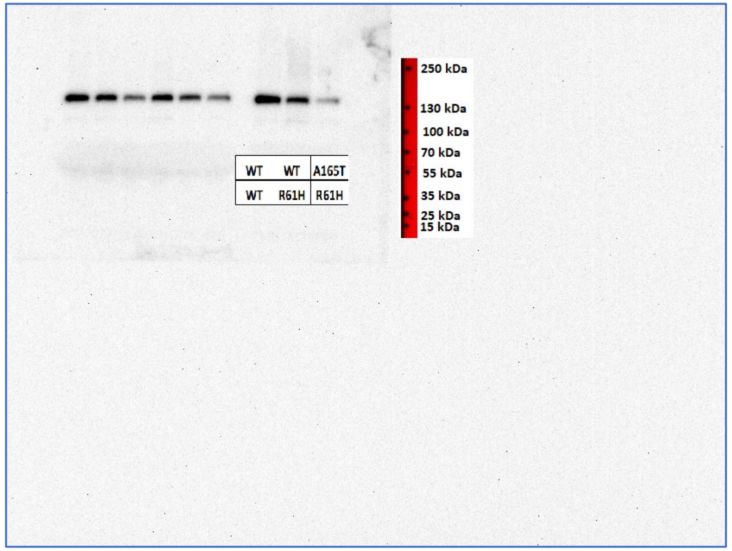

1E

DDK-cells

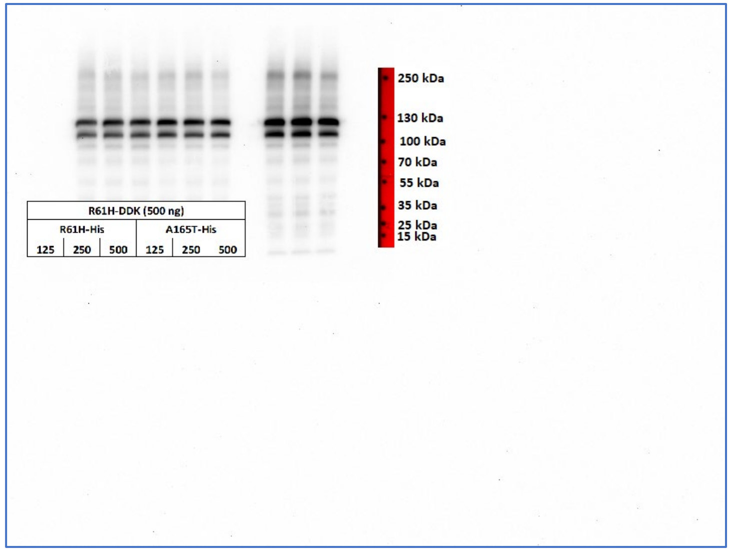

His-cells

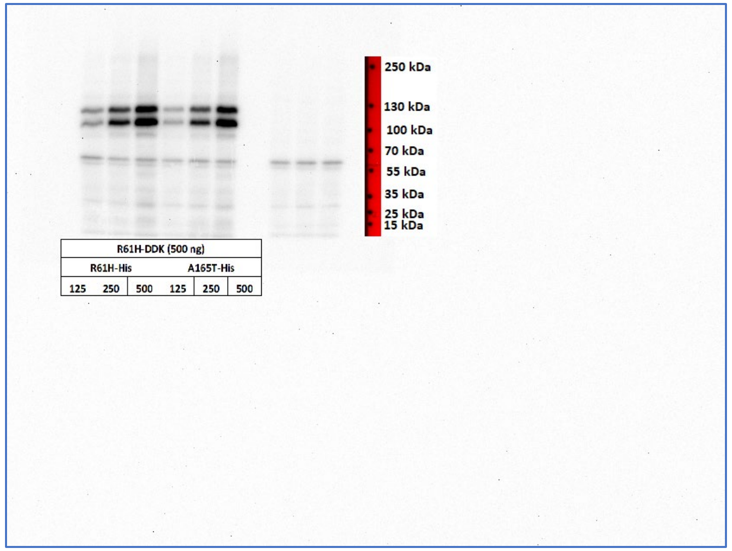

DDK-CM

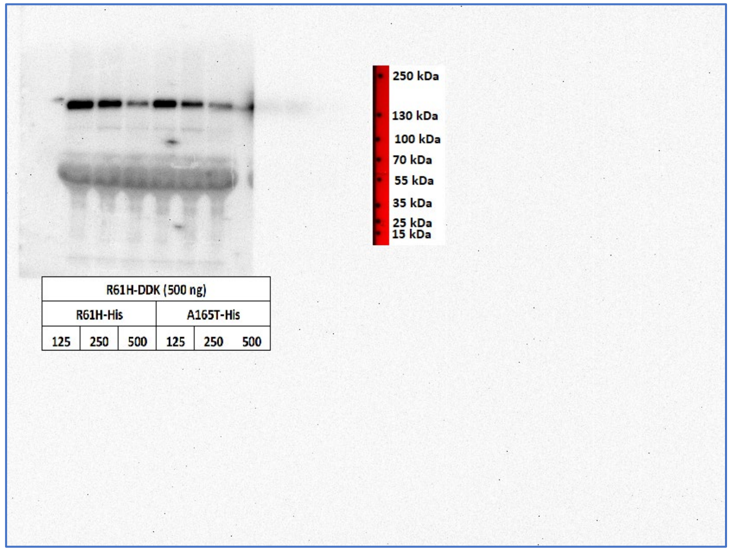

GAPDH-Cells

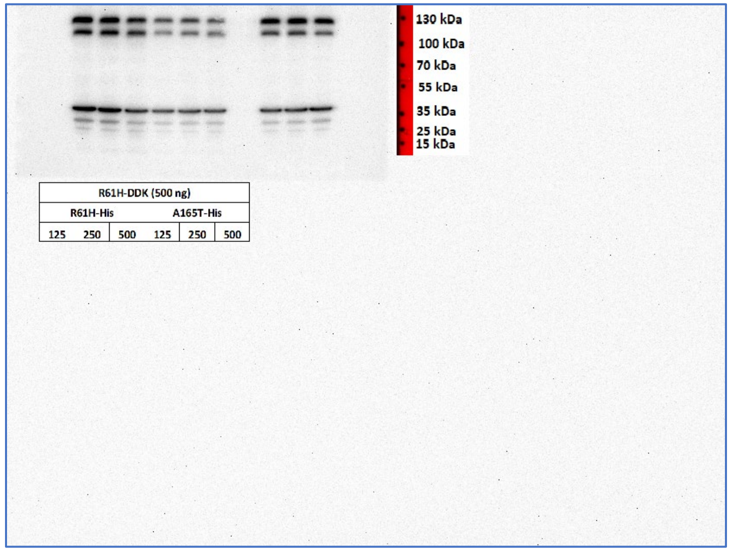

1G

ADAMTSL2

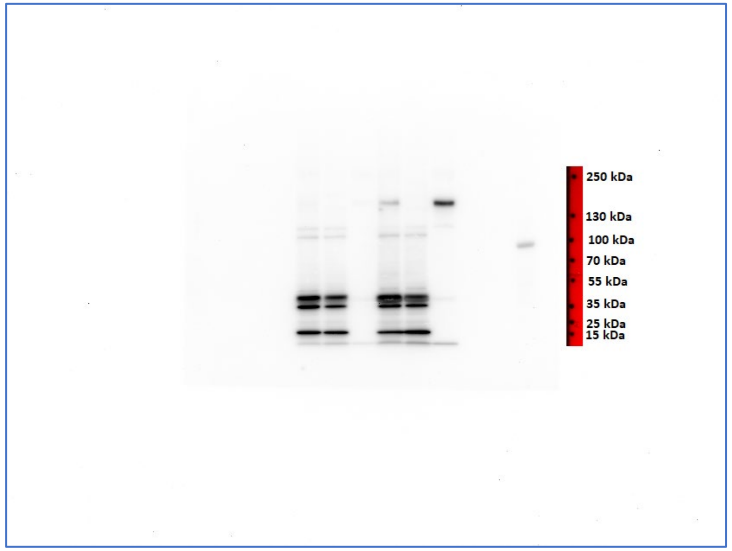

GAPDH

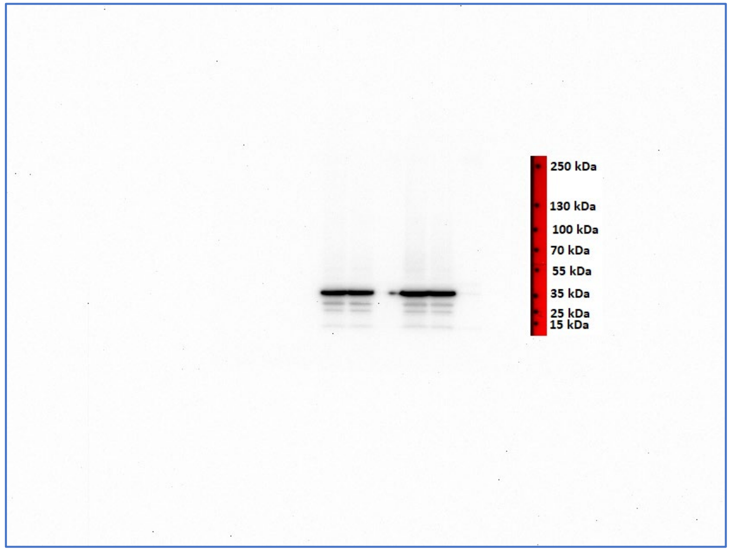

Fibronectin

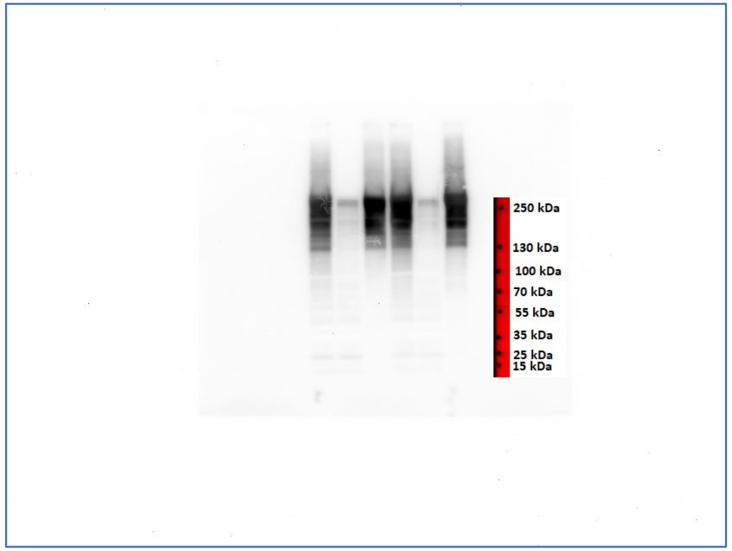

ADAMTSL2 (bottom cover)

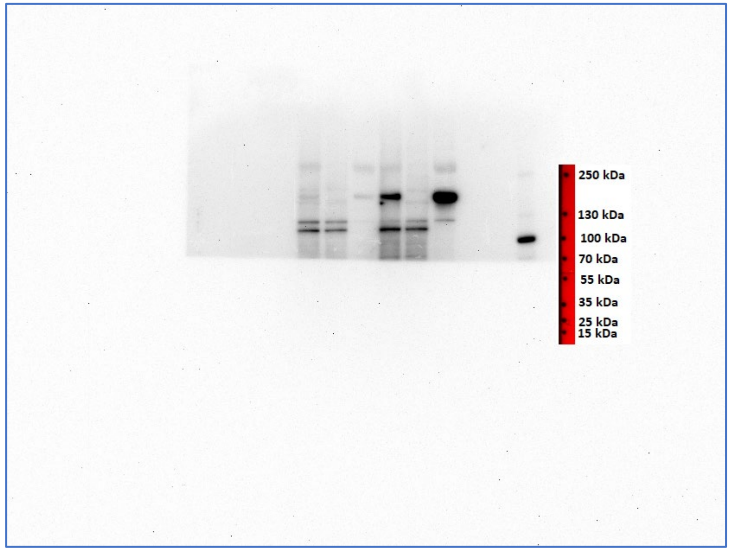

8A

ADAMTSL2

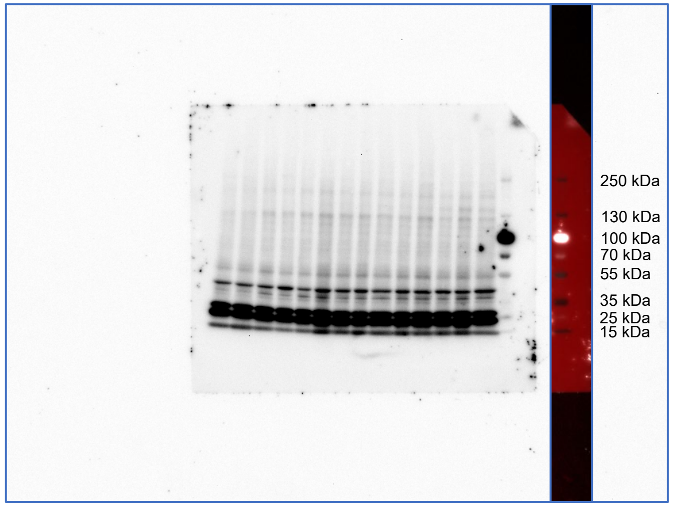

GAPDH

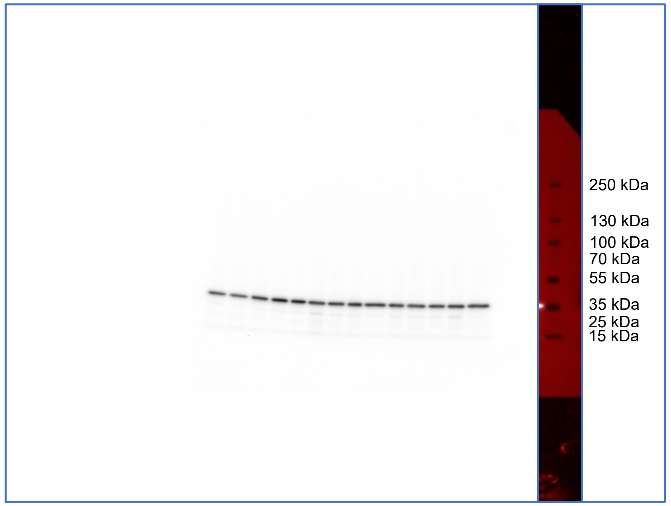

ADAMTSL2 (bottom cover)

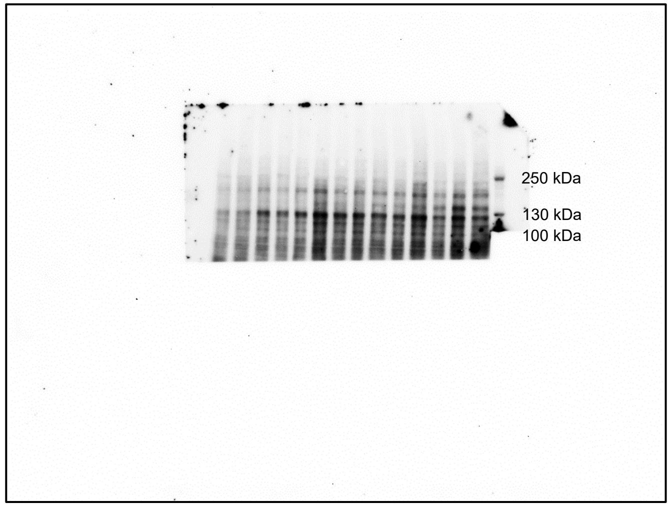

8B

ADAMTSL2

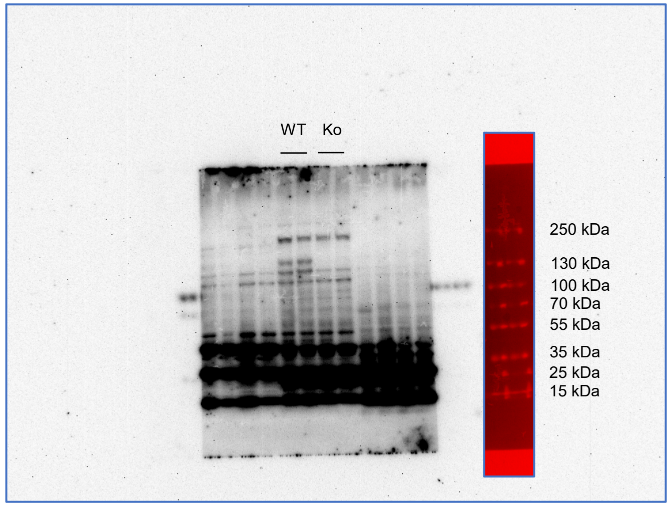

GAPDH

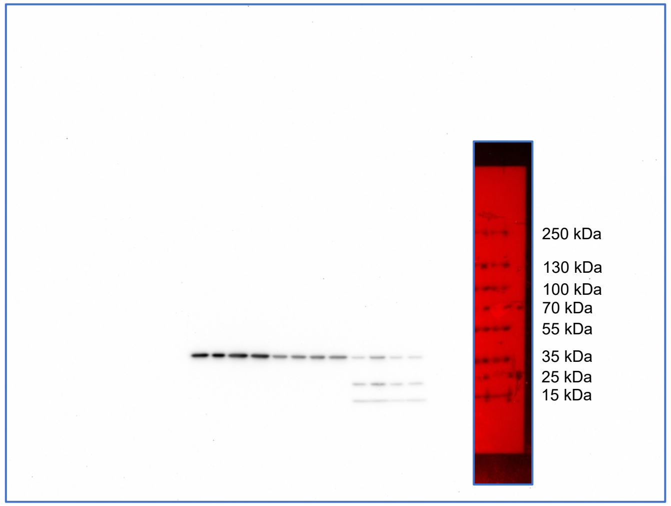

ADAMTSL2 (bottom cover)

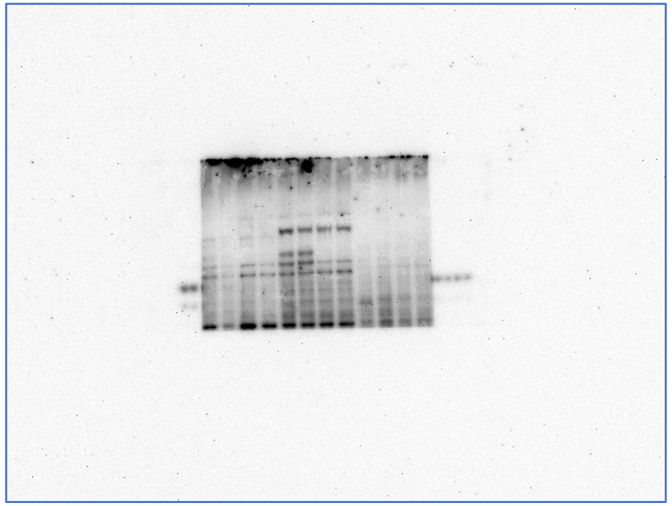

1S1 A

DDK-ADAMTSL2

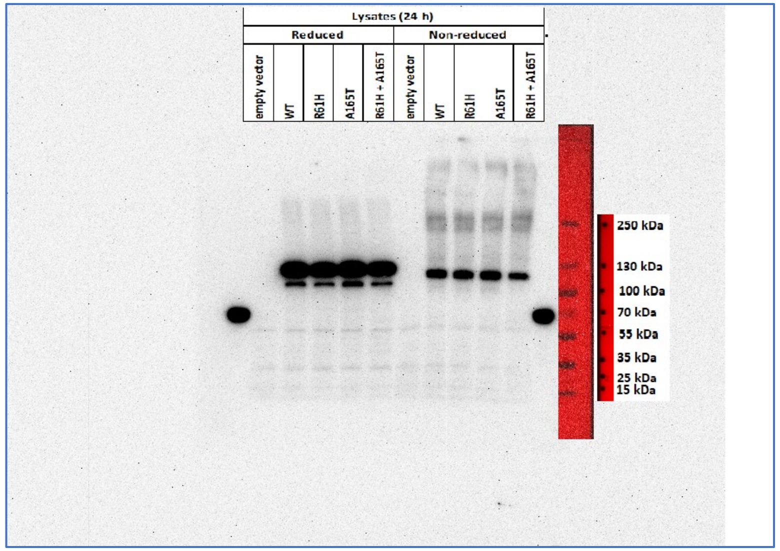

GAPDH

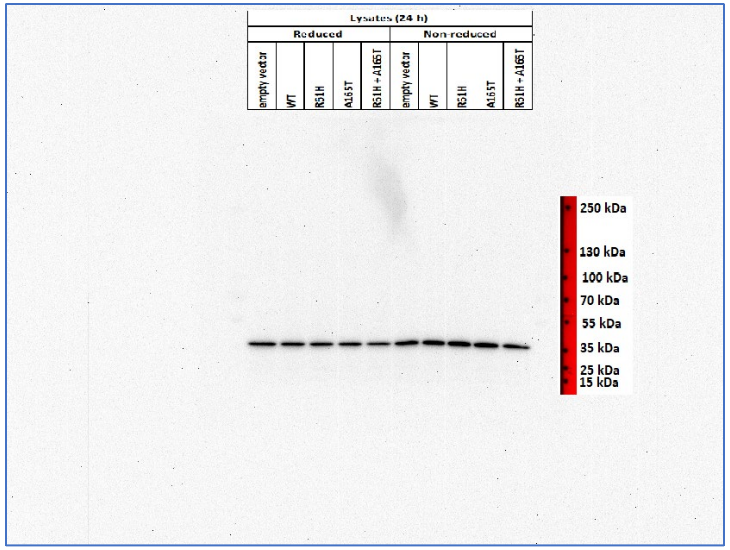

1S1 B

DDK-ADAMTSL2

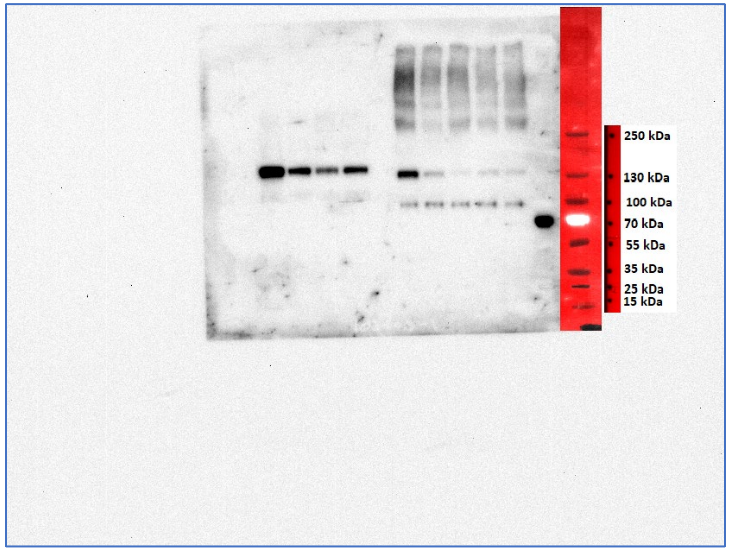

1S2 A

ADAMTSL2

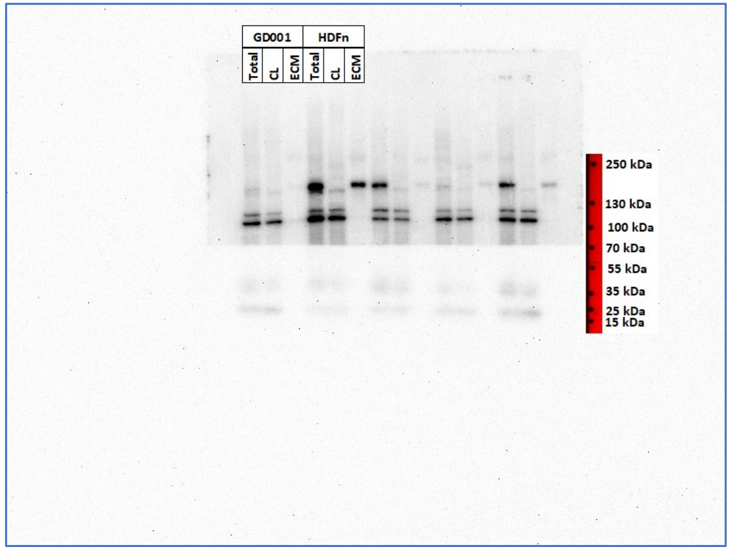

GAPDH

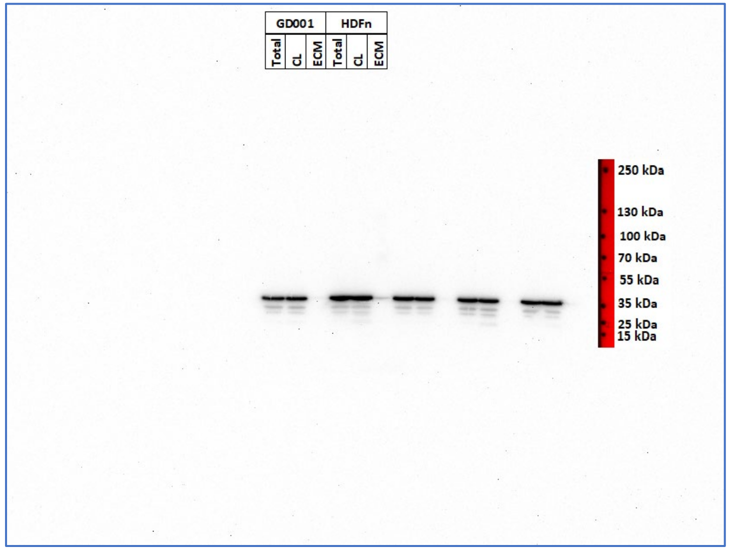

Fibronectin

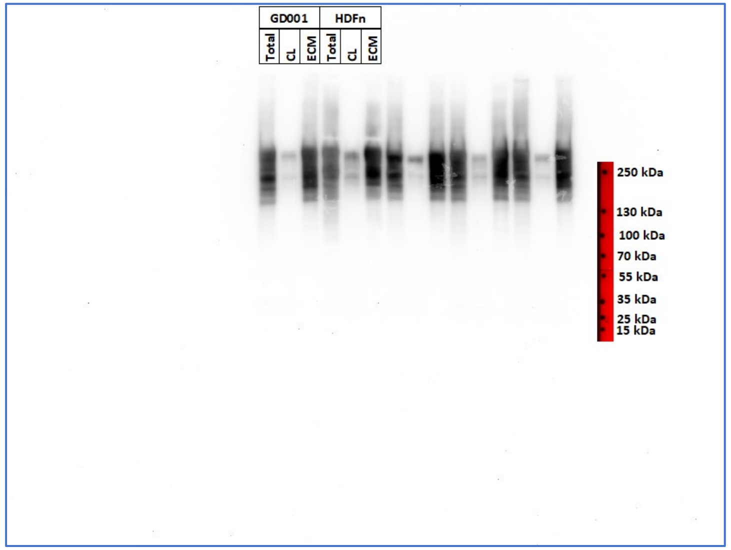

FBN1

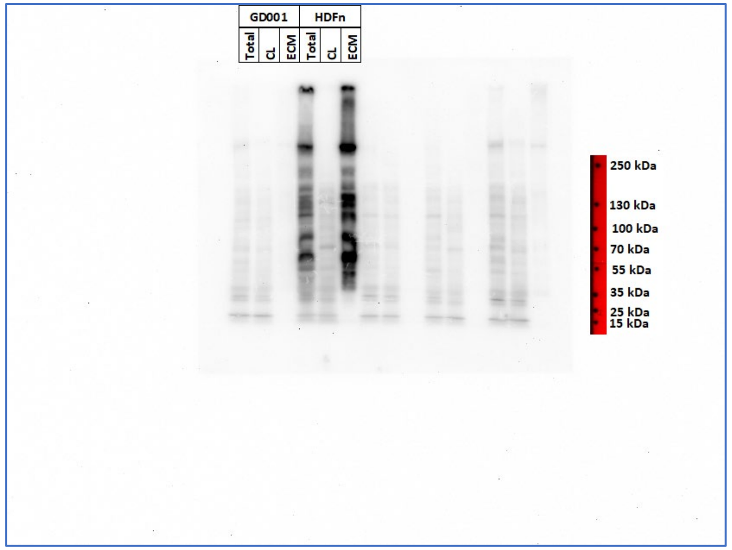

Col1A1

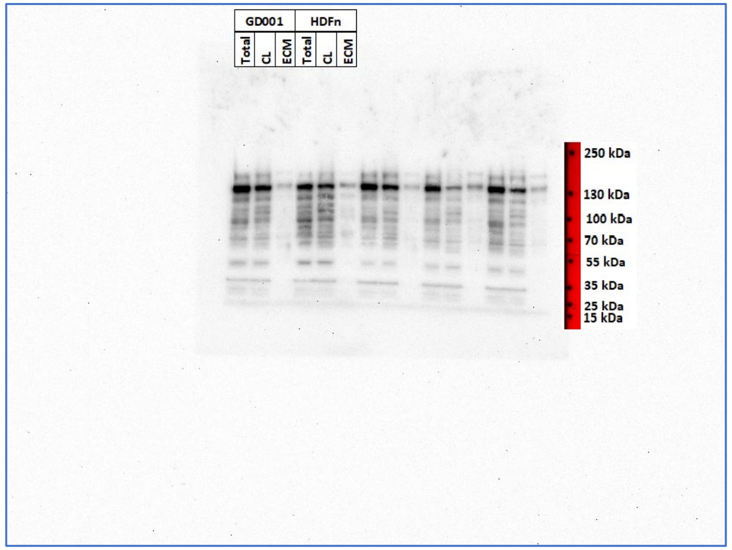

Col3A1

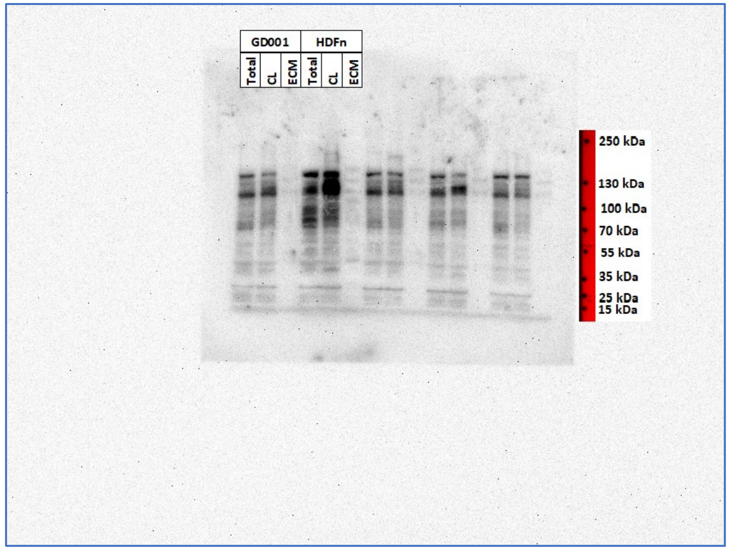

1S3 B

Smad2

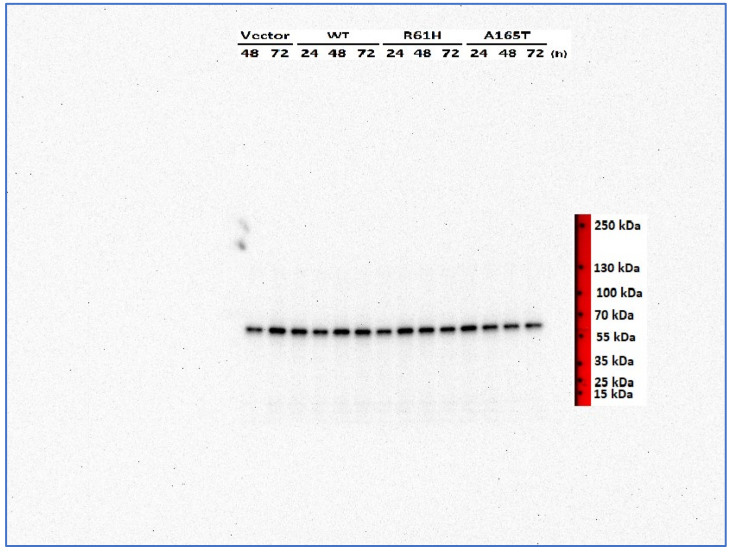

pSmad2

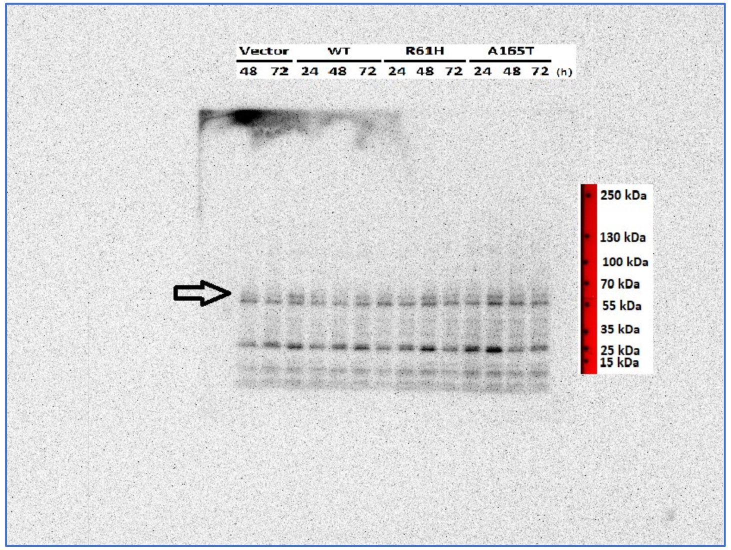

GAPDH

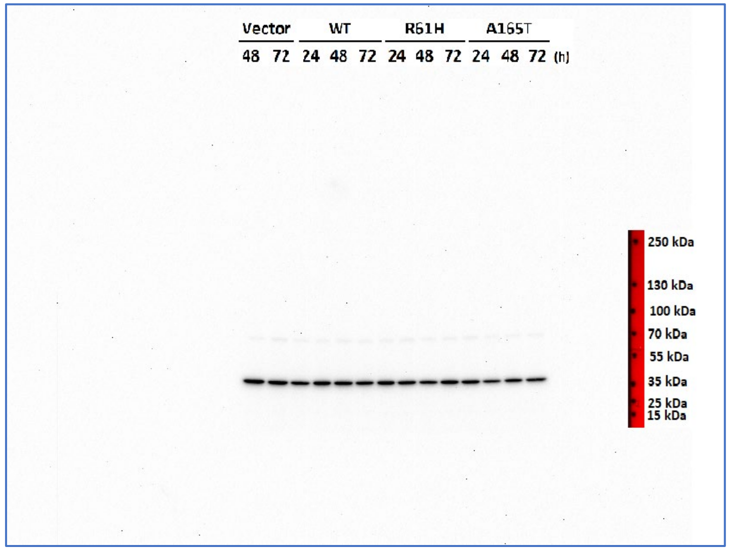

1S3 E

Smad2

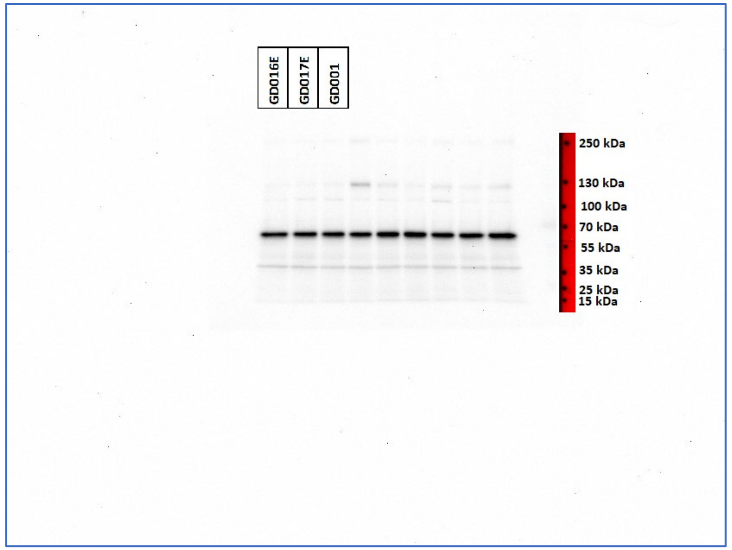

pSmad2

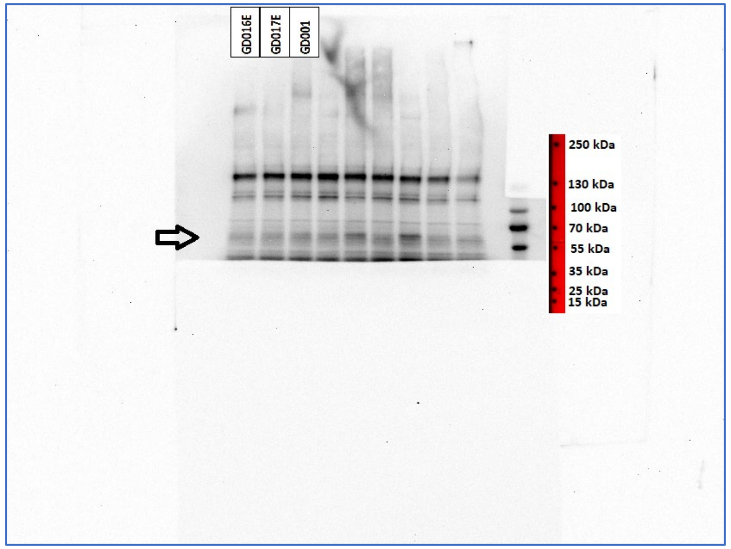

GAPDH

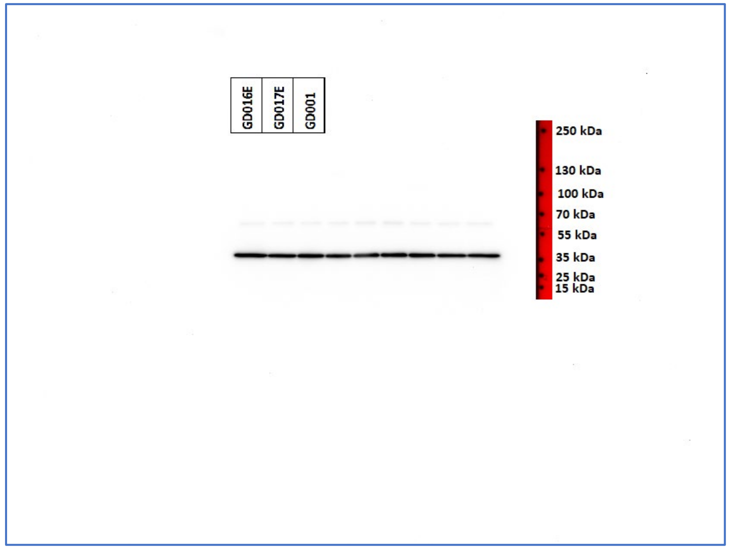

Smad3

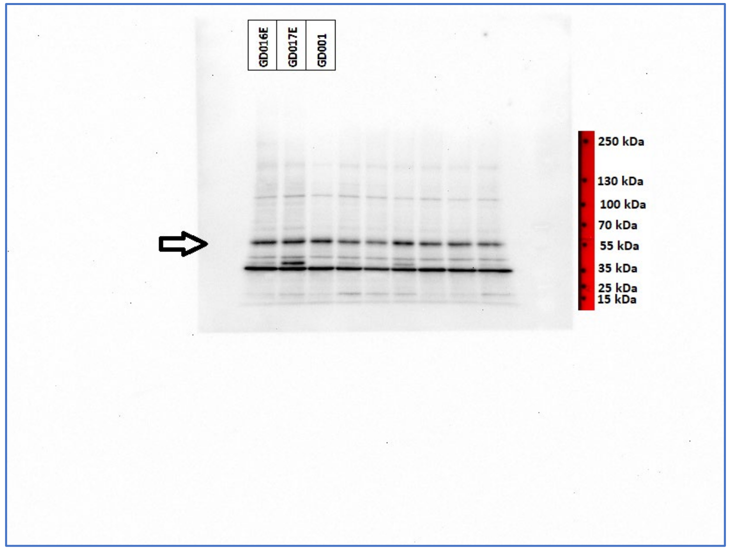

pSmad3

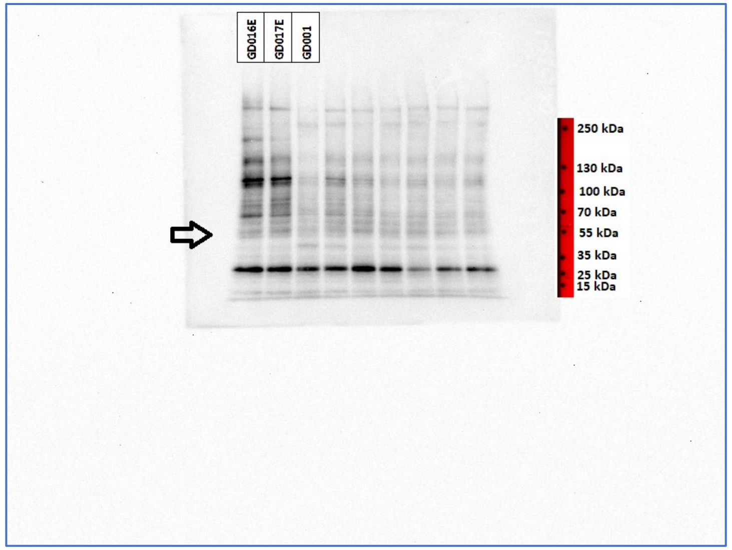

GAPDH

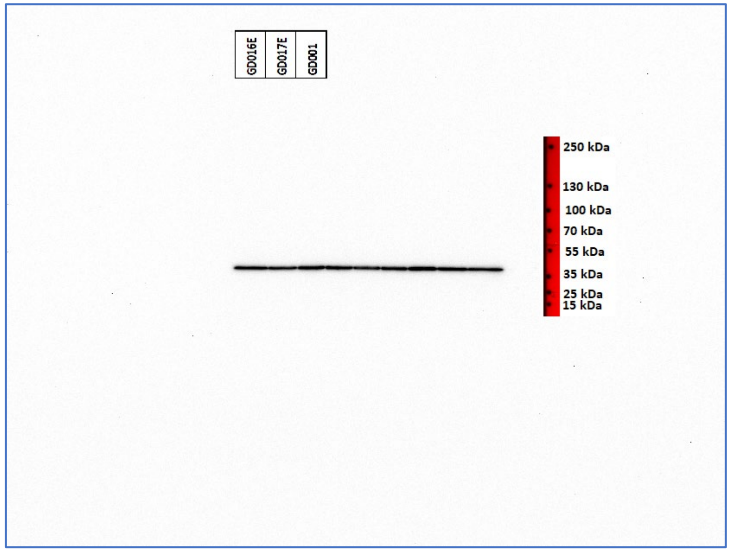

1S3 E

Smad4

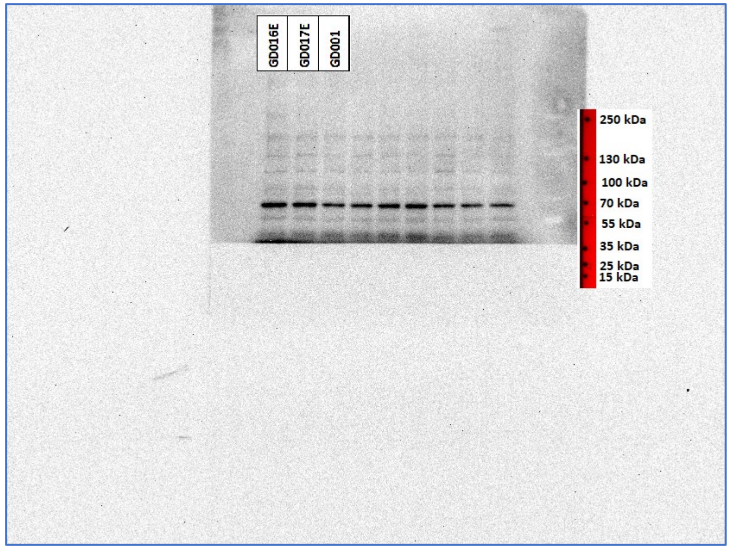

GAPDH

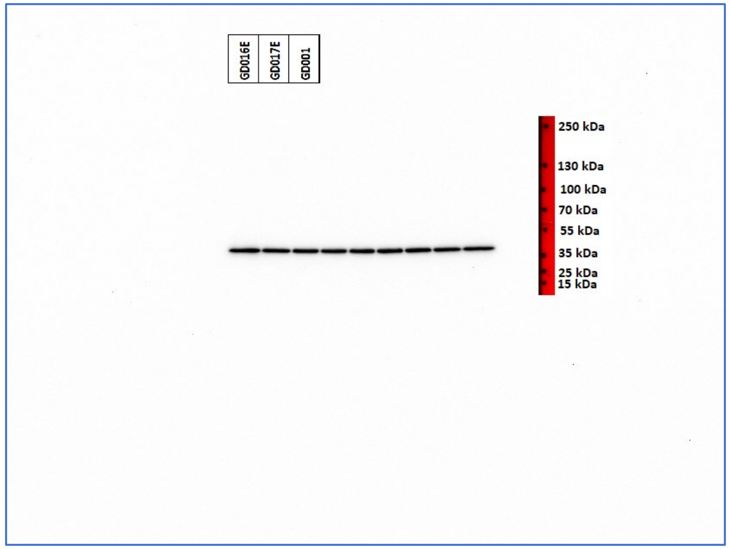

1S3 G

Smad2

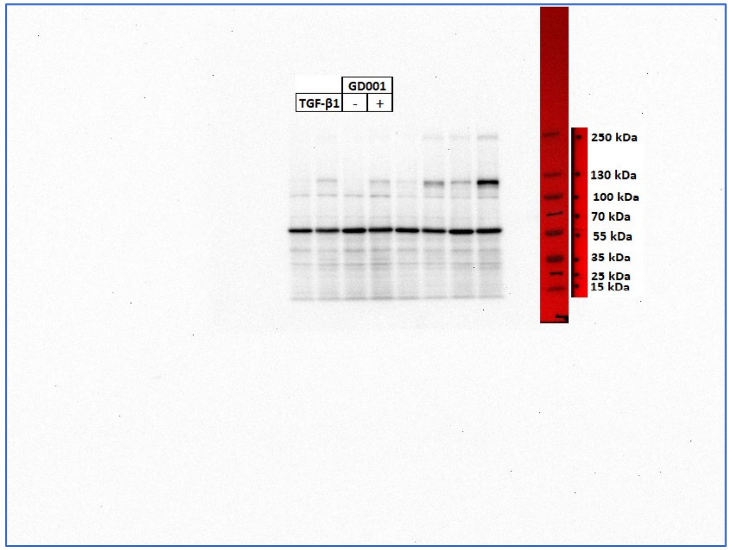

pSmad2

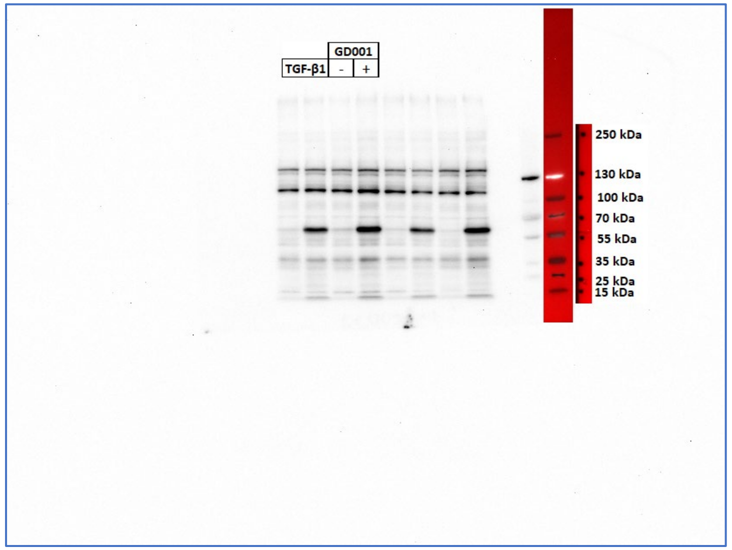

GAPDH

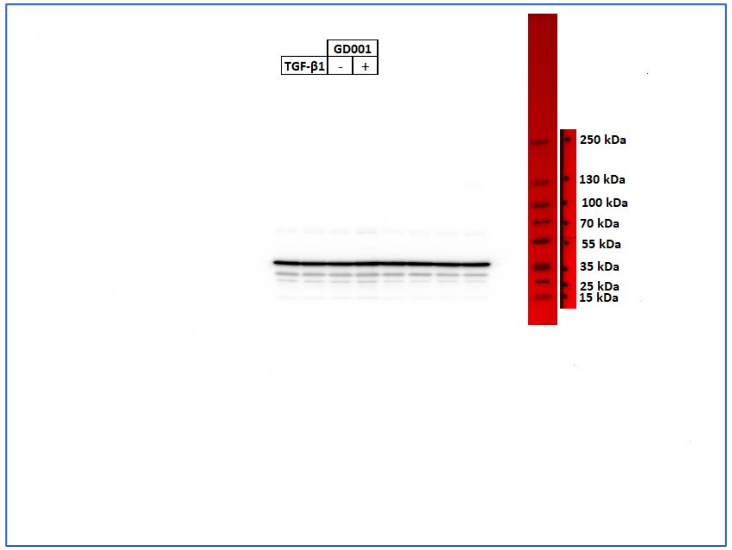

1S4 A

pGSK3β

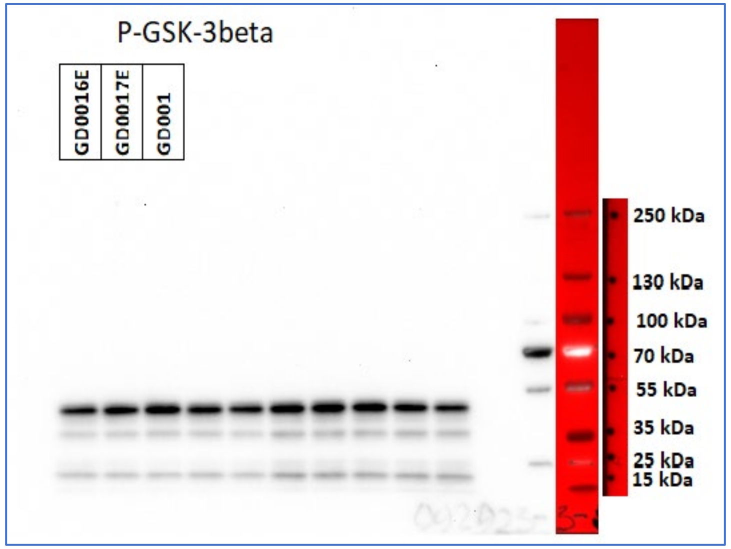

GSK3β

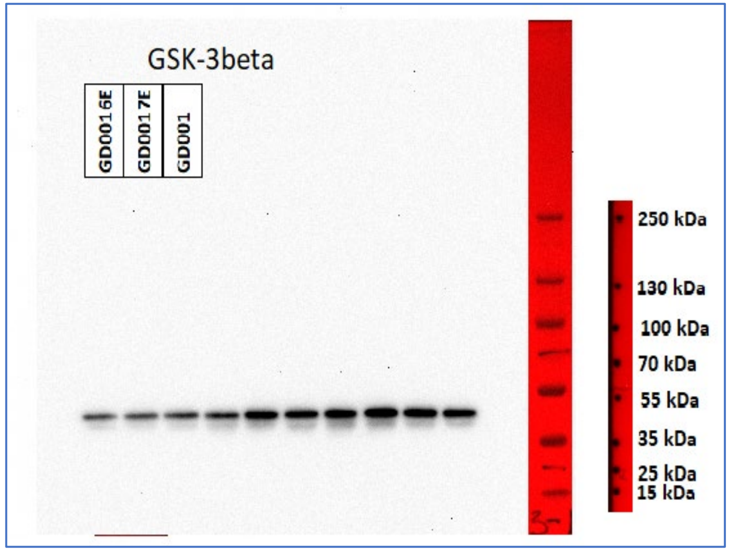

βCatenin

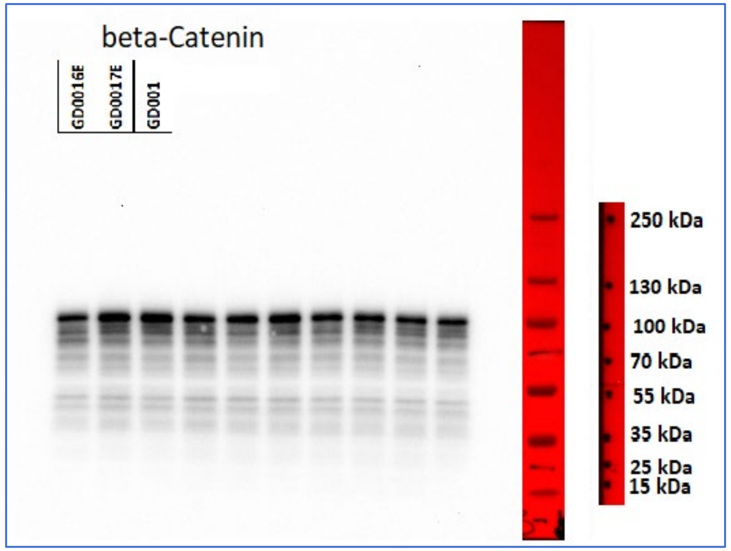

GAPDH

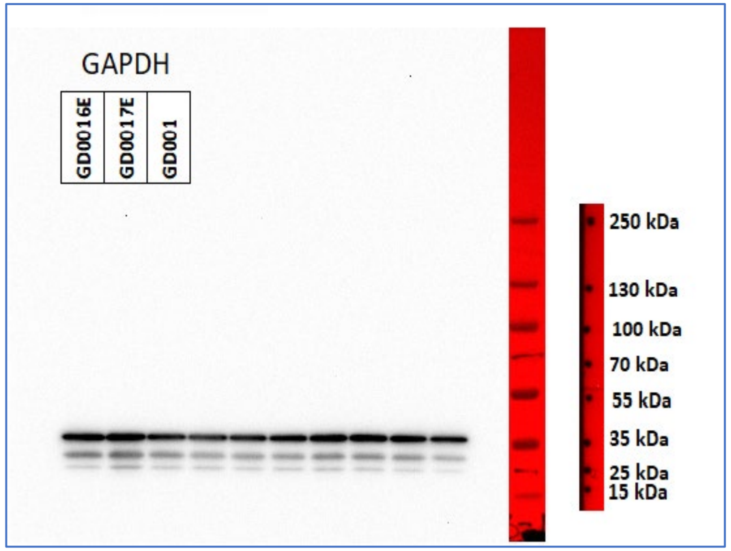

1S4 A

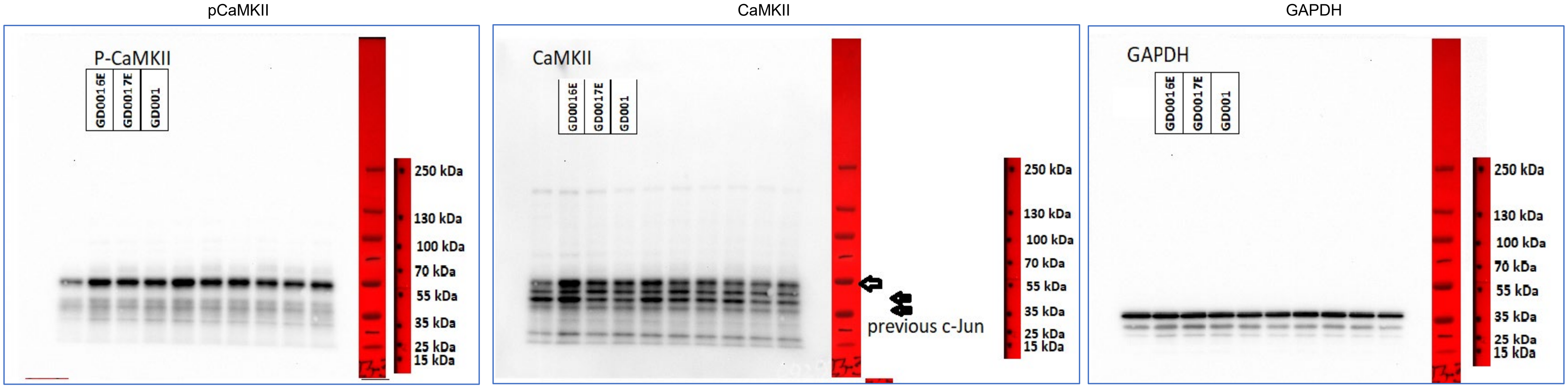
pCaMKII
P-CaMKII
GD0016E
GD0017E
GD001
250 kDa
130 kDa
100 kDa
70 kDa
55 kDa
35 kDa
25 kDa
15 kDa
CaMKII
CaMKII
GD0016E
GD0017E
GD001
previous c-Jun
250 kDa
130 kDa
100 kDa
70 kDa
55 kDa
35 kDa
25 kDa
15 kDa
GAPDH
GAPDH
GD0016E
GD0017E
GD001
250 kDa
130 kDa
100 kDa
70 kDa
55 kDa
35 kDa
25 kDa
15 kDa

1S4 A

pAKT(S473)

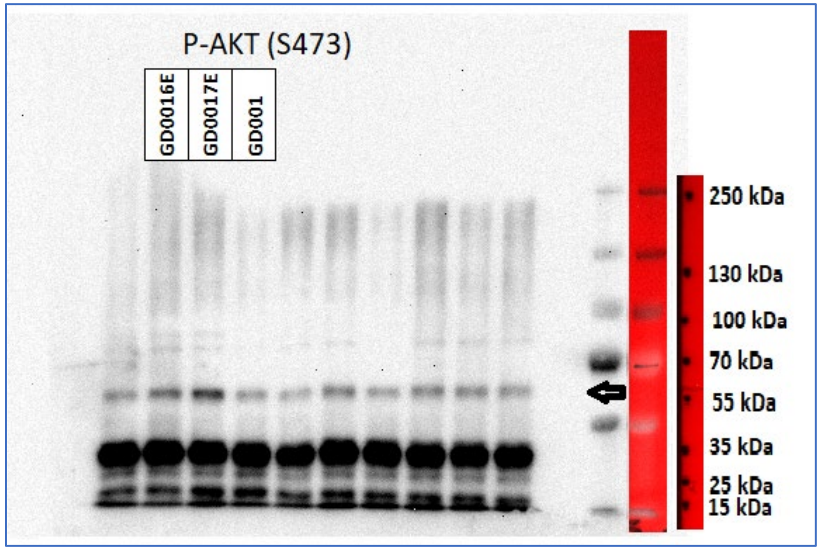

pAKT(T308)

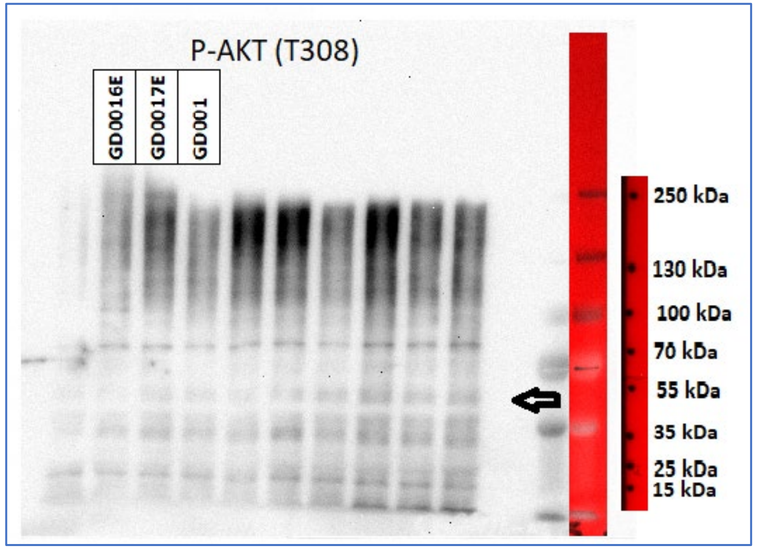

AKT

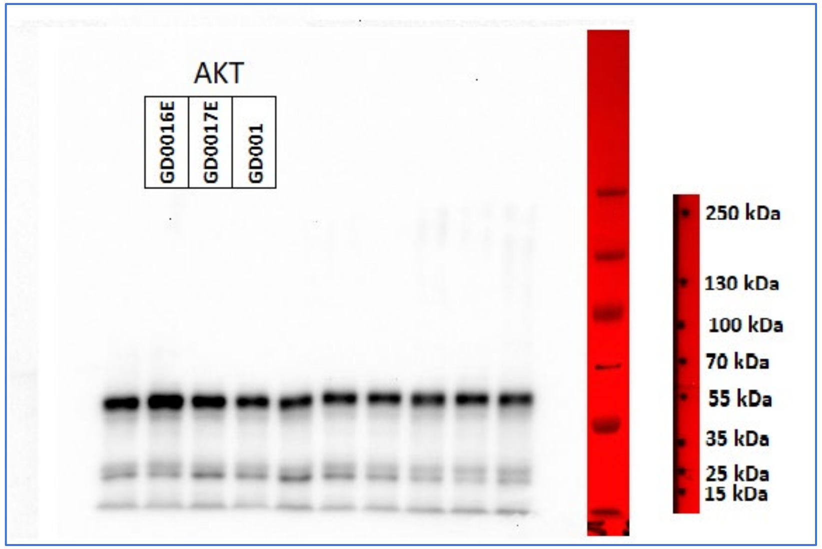

pERK1/2

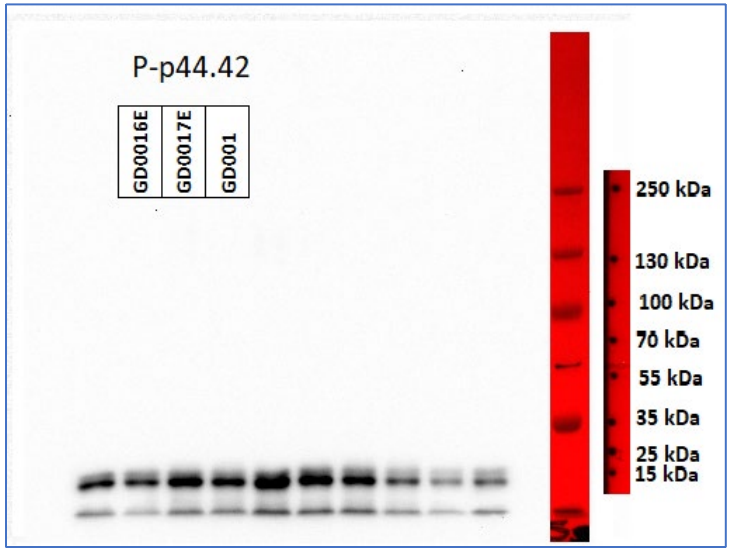

ERK1/2

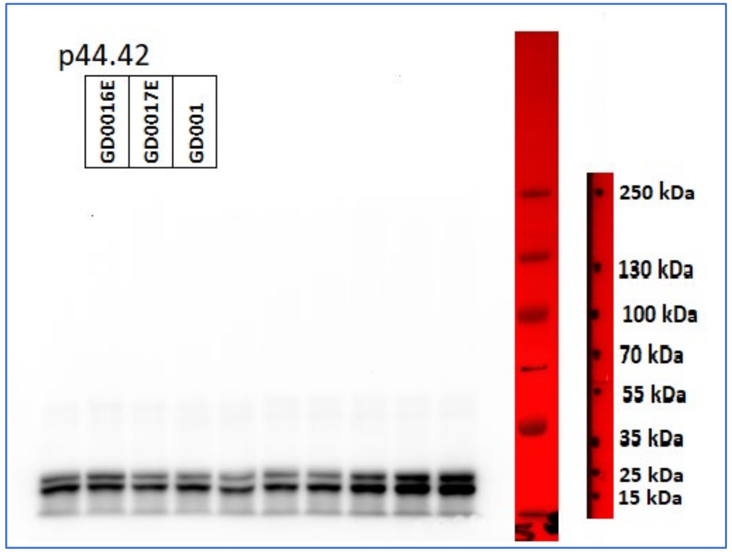

GAPDH

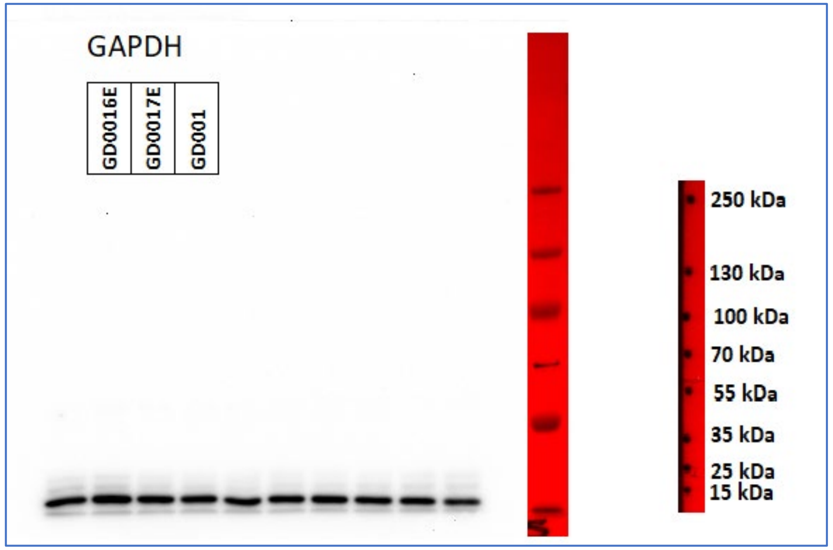

8S1A

βCatenin

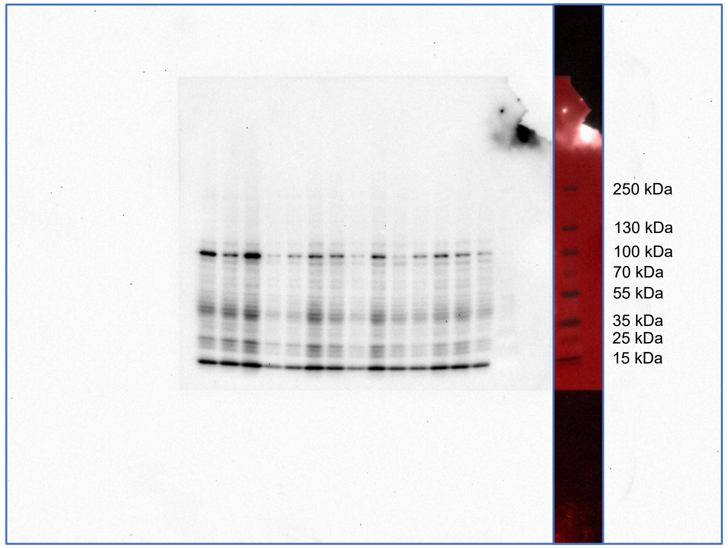

pAKT

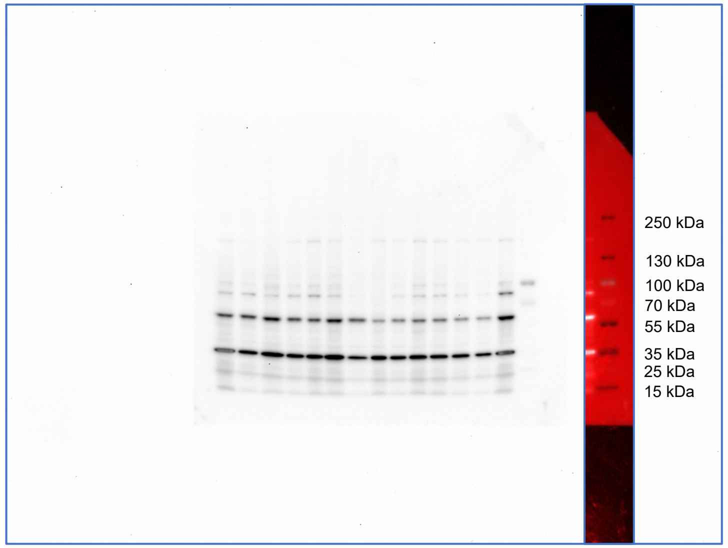

AKT

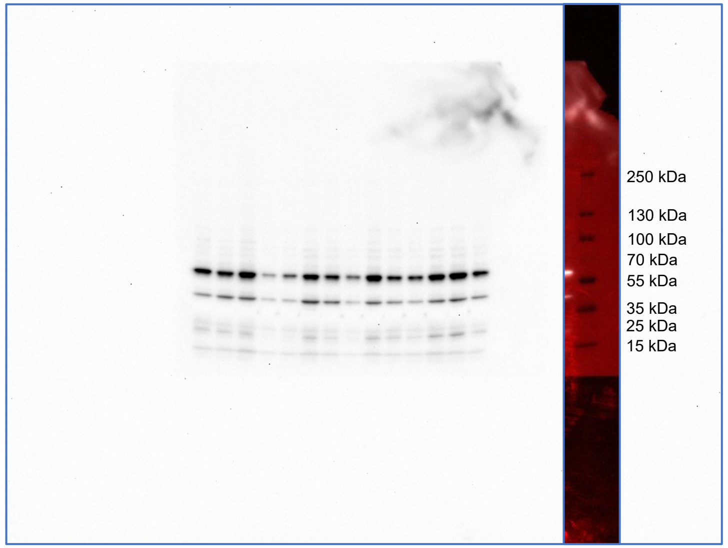

GAPDH

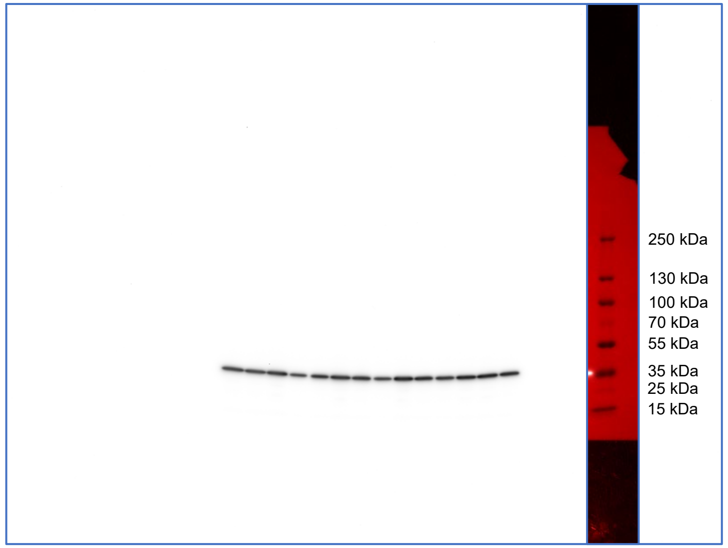

8S1A

pSmad2

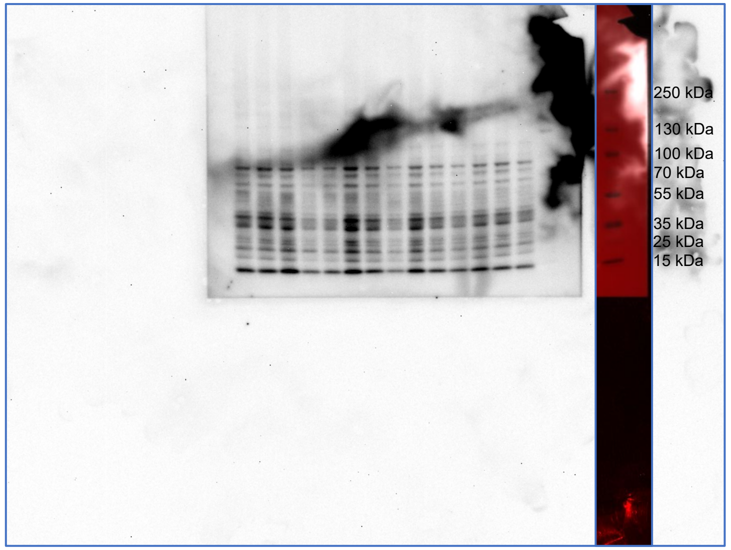

Smad2

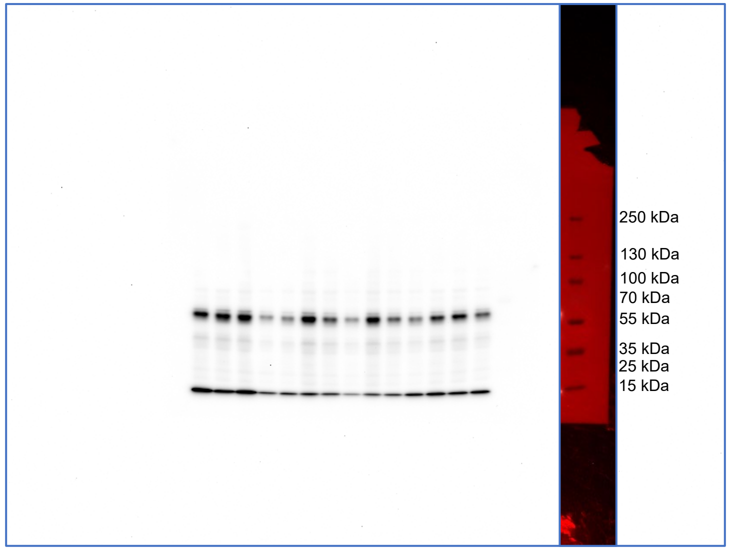

GAPDH

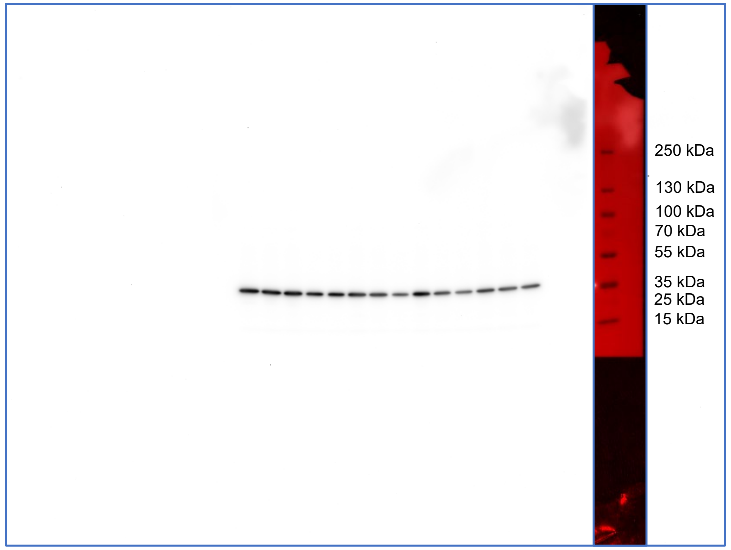

8S1A

pGSK3β

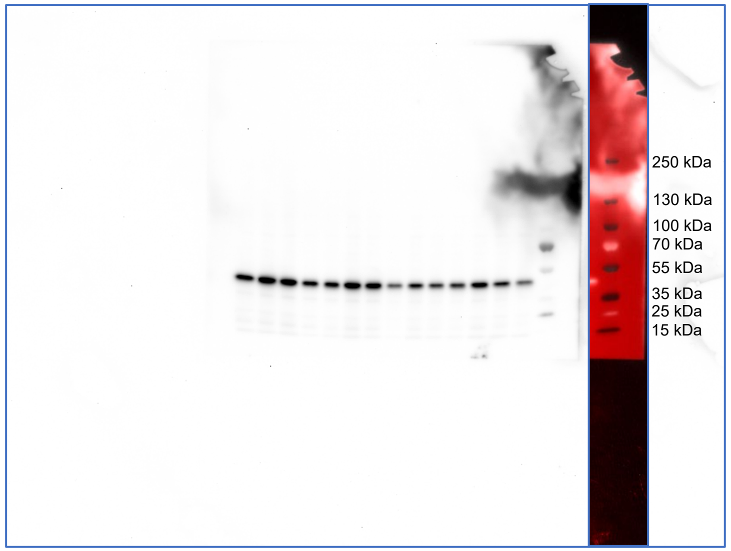

GSK3β

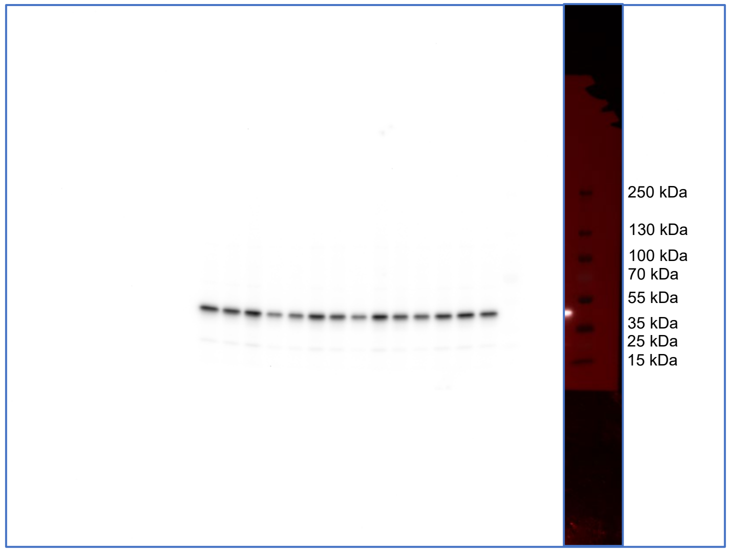

GAPDH

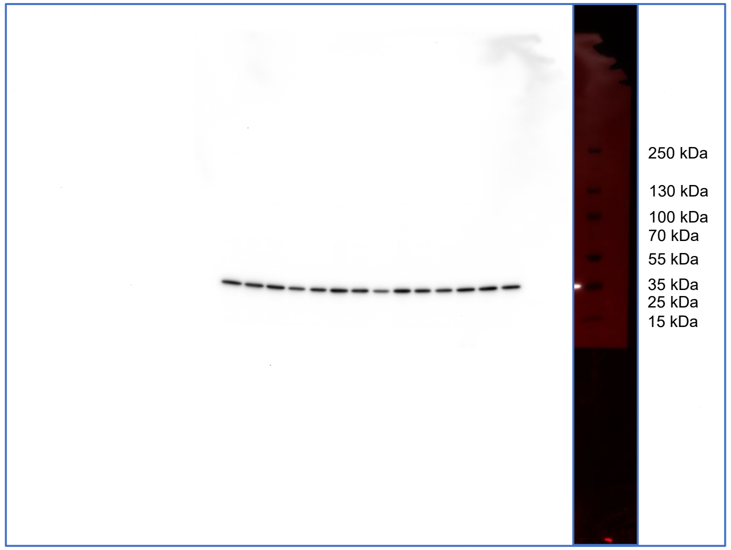

8S2A

βCatenin

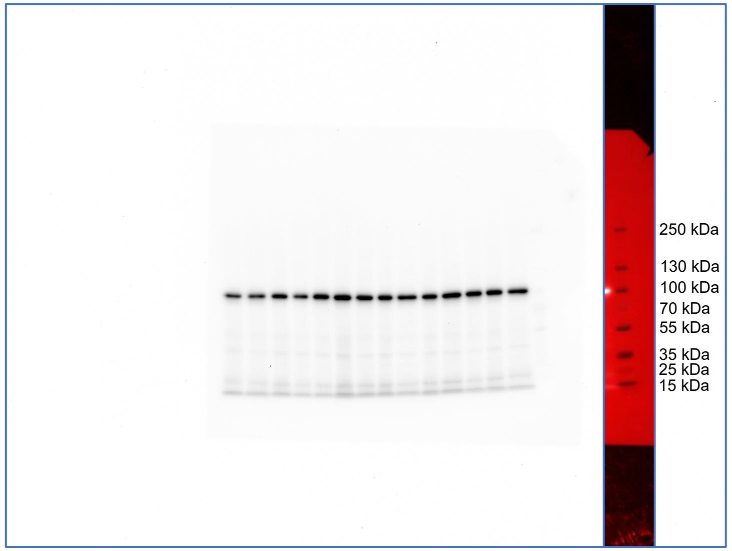

GAPDH

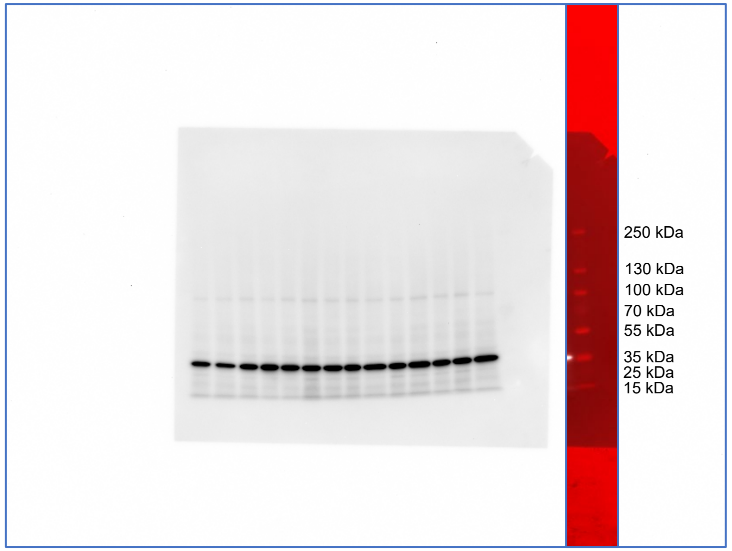

8S2A

pSmad1,5,8
(S463/S465)

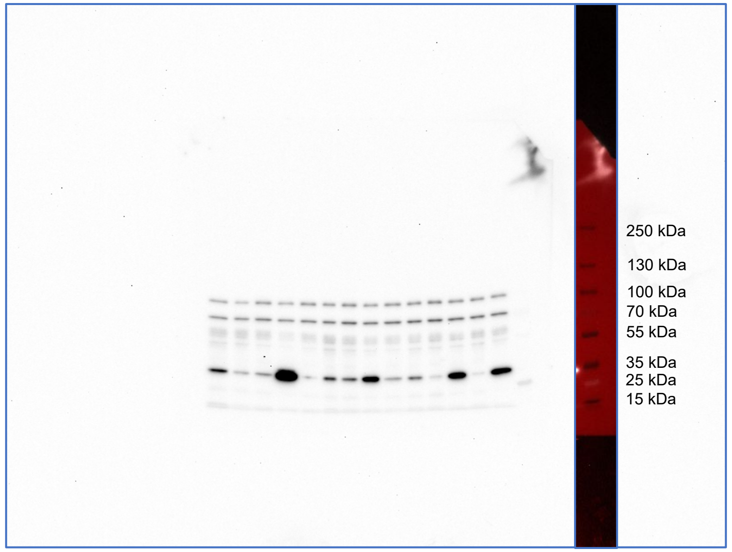

Smad1

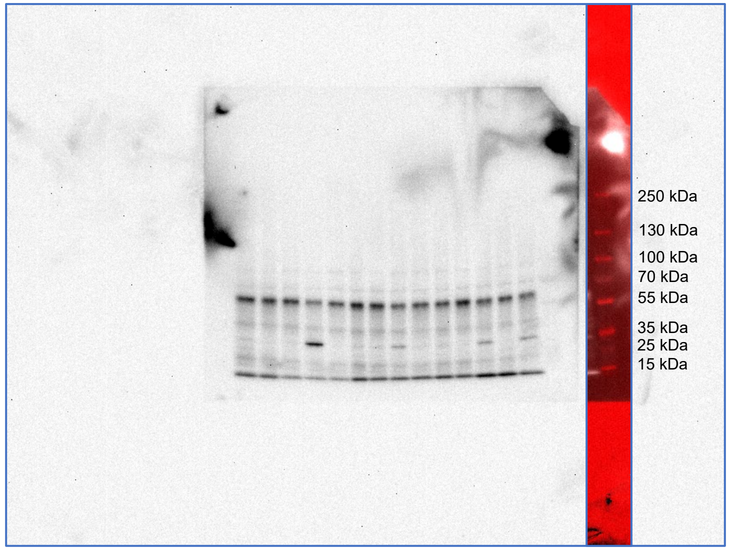

GAPDH

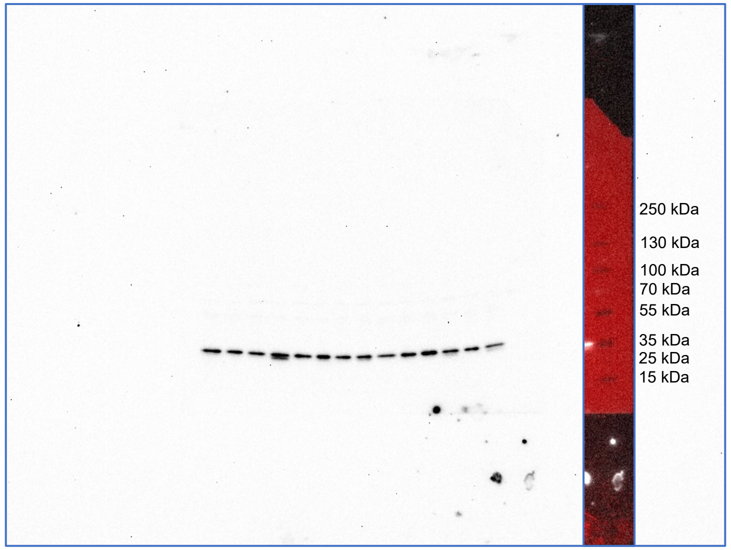

8S2A

pSmad2
(S465/S467)

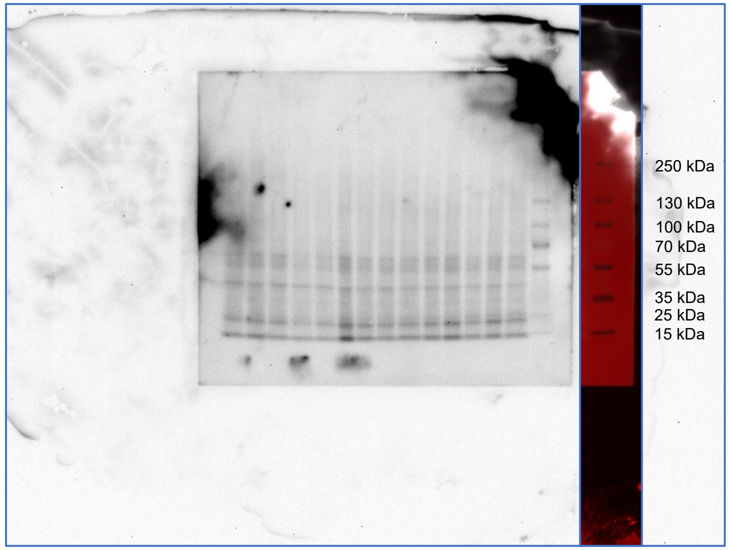

Smad2

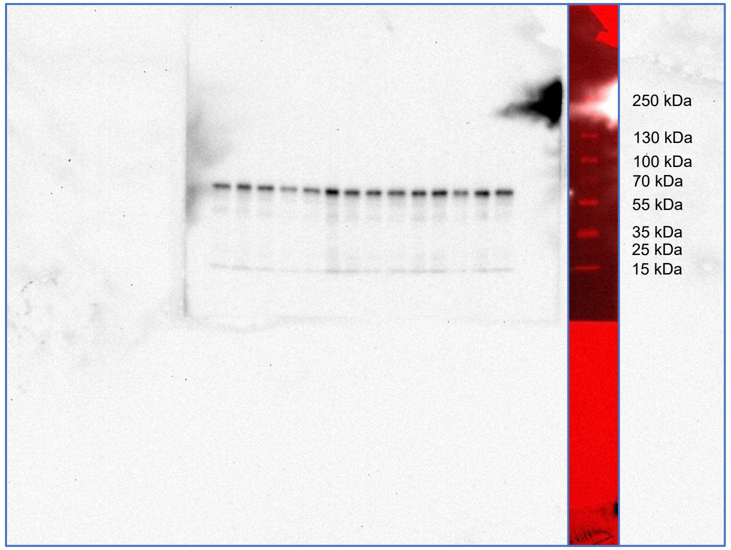

8S2A

pGSK3β

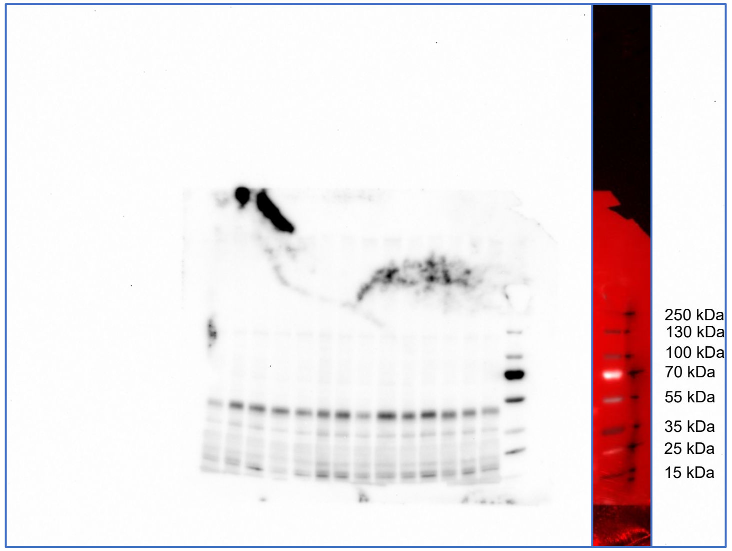

GSK3β

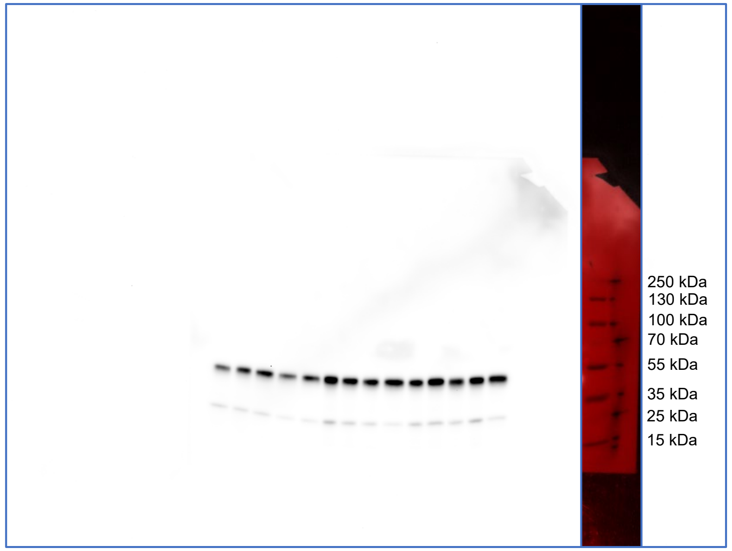

GAPDH

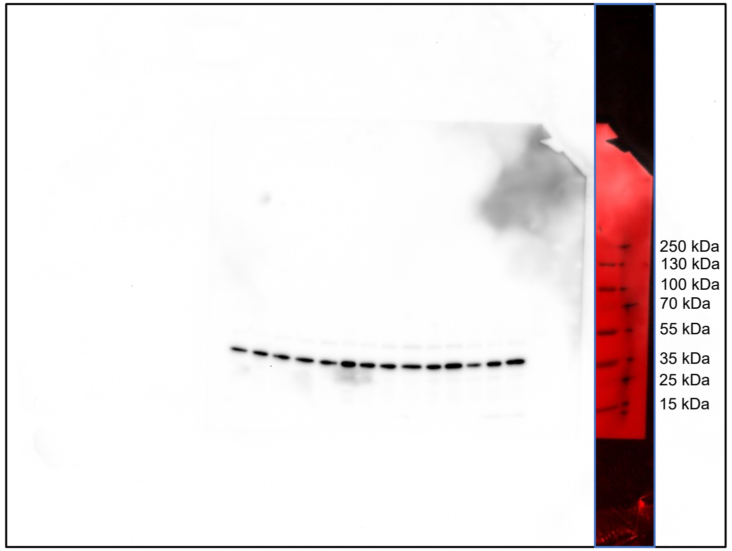

Supplement: Unedited blot and gel images [file jciinsight-9-174417-s031.pdf]
